# A Minor Subpopulation of Mycobacteria Inherently Produces High Levels of Reactive Oxygen Species That Generate Antibiotic Resisters at High Frequency From Itself and Enhance Resister Generation From Its Major Kin Subpopulation

**DOI:** 10.3389/fmicb.2019.01842

**Published:** 2019-08-13

**Authors:** Rashmi Ravindran Nair, Deepti Sharan, Parthasarathi Ajitkumar

**Affiliations:** Department of Microbiology and Cell Biology, Indian Institute of Science, Bengaluru, India

**Keywords:** mycobacterial subpopulations, ROS level heterogeneity, NADH oxidase, labile iron, antibiotic resisters

## Abstract

Antibiotic-exposed bacteria produce elevated levels of reactive oxygen species (ROS), to which either they succumb or get mutated genome-wide to generate antibiotic resisters. We recently showed that mycobacterial cultures contained two subpopulations, short-sized cells (SCs; ∼10%) and normal/long-sized cells (NCs; ∼90%). The SCs were significantly more antibiotic-susceptible than the NCs. It implied that the SCs might naturally be predisposed to generate significantly higher levels of ROS than the NCs. This in turn could make the SCs more susceptible to antibiotics or generate more resisters as compared to the NCs. Investigation into this possibility showed that the SCs in the actively growing mid-log phase culture naturally generated significantly high levels of superoxide, as compared to the equivalent NCs, due to the naturally high expression of a specific NADH oxidase in the SCs. This caused labile Fe^2+^ leaching from 4Fe-4S proteins and elevated H_2_O_2_ formation through superoxide dismutation. Thus, the SCs of both *Mycobacterium smegmatis* and *Mycobacterium tuberculosis* inherently contained significantly higher levels of H_2_O_2_ and labile Fe^2+^ than the NCs. This in turn produced significantly higher levels of hydroxyl radical through Fenton reaction, promoting enhanced antibiotic resister generation from the SCs than from the NCs. The SCs, when mixed back with the NCs, at their natural proportion in the actively growing mid-log phase culture, enhanced antibiotic resister generation from the NCs, to a level equivalent to that from the unfractionated whole culture. The enhanced antibiotic resister generation from the NCs in the reconstituted SCs-NCs natural mixture was found to be due to the high levels of H_2_O_2_ secreted by the SCs. Thus, the present work unveils and documents the metabolic designs of two mycobacterial subpopulations where one subpopulation produces high ROS levels, despite higher susceptibility, to generate significantly higher number of antibiotic resisters from itself and to enhance resister generation from its kin subpopulation. These findings show the existence of an inherent natural mechanism in both the non-pathogenic and pathogenic mycobacteria to generate antibiotic resisters. The presence of the SCs and the NCs in the pulmonary tuberculosis patients’ sputum, reported by us earlier, alludes to the clinical significance of the study.

## Introduction

Bacterial systems maintain heterogeneity in diverse physiological, morphological and metabolic aspects among individual subpopulations within the whole population and among individual cells within a subpopulation (Davidson and Surette, 2008). Such heterogeneity confers striking differences on the response of the subpopulations within the whole population and of individual cells within a subpopulation to diverse stress conditions bestowing survival benefit. With mycobacteria being no exception to this behavior, presence of stress-tolerant short-sized *Mycobacterium smegmatis* cells (Nyka, 1974; Smeulders et al., 1999; Thanky et al., 2007) ovoid, non-culturable, antibiotic/heat-resistant, hypometabolic *M. smegmatis* cells (Anuchin et al., 2009), L-form variants, coccoid forms, and granular forms of *Mycobacterium tuberculosis* (Markova et al., 2012) have been reported. Short ultrafine forms of drug-tolerant *M. tuberculosis* have been found in 82% of tuberculosis patients with open cavity (Khomenko, 1987). Phenotypic variants of *M. tuberculosis* have been found in macrophages and in infected animal models as well (Manina et al., 2015). Besides phenotypic heterogeneity driven stress tolerance, diverse stress agents, including antibiotics, induce high levels of oxidative stress in bacterial cells due to the generation of reactive oxygen species (ROS) that inflict genome-wide mutations from which target-specific mutants get selected (Ryan, 1959; Dwyer et al., 2009; Li G.Q. et al., 2015; Knöppel et al., 2017; Hoeksema et al., 2018; Jin et al., 2018). In this regard, antibiotics-exposed bacteria, including mycobacteria, have been found to generate high levels of ROS that in turn could induce genome-wide mutations, which enabled selection of resistant mutants against antibiotics (Mwangi et al., 2007; Kohanski et al., 2010; Grant et al., 2012; Sun et al., 2012; Piccaro et al., 2014).

In the background of these studies, we reported the presence of a minor subpopulation (∼10% of the population) of low buoyant density, short-sized cells (SCs) and a major subpopulation (∼90% of the population) of high buoyant density, normal/long-sized cells (NCs) in the actively growing mid-log phase (MLP) cultures of *M. tuberculosis* (*Mtb*), *M. smegmatis* (*Msm*), and *Mycobacterium xenopi* (Vijay et al., 2014a,b, 2017). The tubercle bacilli isolated from the freshly diagnosed pulmonary tuberculosis patients’ sputum also contained the SCs and the NCs (Vijay et al., 2014b). Both *Mtb* and *Msm* SCs were found to be significantly more susceptible to anti-tuberculosis drugs, rifampicin (RIF), and isoniazid (INH), and to H_2_O_2_ and acidified sodium nitrite (Vijay et al., 2017). Further, the RIF/INH-exposed SCs showed significantly elevated levels of ROS, superoxide and hydroxyl radical, unlike the NCs (Nair et al., 2019). These observations alluded to the possibility that the SCs might be inherently predisposed to produce higher levels of ROS than the NCs in the native antibiotics-unexposed condition. Further, the inherent generation of higher ROS levels raises the possibility of infliction of genome-wide mutations in these cells to get selected when confronted with antibiotics.

In view of this possibility, the present study was designed based on the hypothesis that the SCs isolated from the actively growing mid-log phase population might be having significantly higher levels of ROS, such as superoxide, hydrogen peroxide and hydroxyl radical, than the equivalent NCs. Therefore, the SCs in the mid-log phase population must be having significantly high levels of oxidative stress, as compared to the equivalent NCs, contributed by elevated levels of major ROS such as superoxide, hydrogen peroxide and hydroxyl radical. If this be the case, then these ROS should be detectable and measurable in the SCs isolated freshly from the mid-log phase population, in comparison to their levels in the equivalent NCs. Further, depending upon the levels of which of the ROS were high, the reasons for and the mechanism behind the inherent generation of high levels of specific types of ROS could be studied in the SCs, in comparison to that in the NCs. Therefore, we traced out the ROS-related molecular characteristics of the SCs and the NCs to find out the factors that naturally predisposed the SCs to generate higher levels of ROS, as compared to the NCs. Subsequently, we determined the physiological benefits and the costs of the natural differential ROS generation between the SCs and the NCs to themselves, to their mixture reconstituted at the same proportion as they existed in the MLP culture, and to the unfractionated whole MLP population.

We discovered that the freshly isolated subpopulation of the SCs inherently produced significantly high levels of superoxide, H_2_O_2_ and consequentially contained high levels of leached labile Fe^2+^, which resulted in high levels of hydroxyl radical generation (through Fenton reaction), as compared to the subpopulation of the NCs. This inherent and unique metabolic status of the SCs generates significantly higher number of genetic resisters from the SCs against antibiotics. Further, the H_2_O_2_ secreted into the medium by the SCs enhanced resister generation from the NCs to a level comparable to that from the unfractionated whole culture, when the SCs and the NCs were reconstituted at the same ratio as they existed together in the mid-log phase culture. Since the SCs and the NCs of *Mtb* cultures also showed identical metabolic status, like those of the *Msm* cultures, and sputum of pulmonary tuberculosis patients contained SCs and NCs of *Mtb*, in the same proportion as they existed *in vitro* culture of *Mtb* (Vijay et al., 2014b), we discussed the clinical implications of the findings with reference to the emergence of antibiotic resistance.

## Materials and Methods

### Bacterial Strains and Culture Conditions

*Mycobacterium smegmatis* mc^2^155 (*Msm*) (Snapper et al., 1990) cells were cultured in Middlebrook 7H9 broth with 0.05% v/v Tween 80, at 37°C, with shaking at 170 rpm, till the culture reached OD_600 nm_ 0.6 (mid-log phase, MLP) or plated on Middlebrook 7H10 or Mycobacteria 7H11 agar in order to determine colony forming units (cfu). *M. tuberculosis* H_37_R_v_ virulent strain (*Mtb*), obtained from Central JALMA Institute for Leprosy and Other Mycobacterial Diseases, Agra, India, was cultured in Middlebrook 7H9 broth with Albumin-Dextrose-Sodium chloride supplement and 0.05% Tween 80 at 37°C with shaking at 170 rpm, till OD_600 nm_ of the culture reached 0.60 (mid-log phase). *Escherichia coli* JM109 was used for cloning purposes. The bacterial strains used are listed in Supplementary Table S1. The detailed procedures of all the methods can be found in the section “Supplementary Materials and Methods” in the Supplementary Material.

### Percoll Density Gradient Centrifugation for the Fractionation of SCs and NCs

The preparative scale Percoll density gradient centrifugation for the enrichment of short cells (SCs) and NCs from *Msm* and *Mtb* cultures to prepare short cell enriched fraction (SCF) and normal/long cell enriched fraction (NCF) was performed, exactly as described (Vijay et al., 2017). The Percoll fractions, 64, 66, and 78% contained the SCF1, SCF2, and NCF cells, respectively, for *Msm*. Whereas, the 60 and 62% were combined to get *Mtb* SCF1, 64 and 66% were used as SCF2 and NCF, respectively, for *Mtb*, as described (Vijay et al., 2017). The details of the preparation and processing of *Msm* and *Mtb* SCF1, SCF2, and NCF cells are given in section “Supplementary Materials and Methods” in the Supplementary Material, as described (Vijay et al., 2017).

### Flow Cytometric Determination of the Redox Status of Unexposed SCF and NCF Cells Using Mrx1-roGFP2 Biosensor

Redox biosensor *Mrx1-rogfp2* was used to determine the redox status of *Msm* SCF and NCF cells. The stable *Msm* integrant cells, which carry single copy of pAKMN2/*hsp60-Mrx1-rogfp2* integrated in the genome at the mycobacteriophage L5 *att* site (Nair et al., 2019), were cultured and SCF and NCF cells were isolated using Percoll density gradient. The *Mrx1-rogfp2* integrated SCF and NCF samples were taken for flow cytometry analyses, with at least 10000 cells gated from each sample. Data was acquired using Becton Dickinson FACSVerse flow cytometer with 405 nm (V500) and 488 nm (FITC) solid state laser and 528/45 nm and 527/32 nm emission filter, respectively, at medium flow rate. A high ratio of V500:FITC fluorescence indicative of increase in V500 fluorescence and decrease in FITC fluorescence shows high oxidative status. For analysis purpose, at least 10000 cells were gated from each sample. The median fluorescence for V500 and FITC was set to 2-log_10_ fluorescence units for the wild-type *Msm* cells for each time point, which served as the autofluorescence control. First, the median fluorescence for both V-500 and FITC was obtained from the gated cells. Subsequently, the ratio of V500:FITC median fluorescence was calculated. In order to analyze the ratio of V500:FITC median fluorescence of different subpopulations from the gated cells, a scatter plot with four quadrants was generated. The median fluorescence of both V500 and FITC for the cells in each quadrant was obtained and for each of the quadrant, the ratio of V500:FITC median fluorescence was calculated. The biosensor response from the *Msm*/pAKMN2**-***hsp60-Mrx1-rogfp2*samples was calculated by dividing the median fluorescence obtained at 405 nm (V500) with that obtained at 488 nm (FITC) and was used for plotting the graph. Statistical significance of the values between the time points was calculated.

### Determination of Hydroxyl Radical Levels Using HPF-Stained Cells

Equal aliquots of *Msm* SCF and NCF cells were stained with 5 μM HPF (Setsukinai et al., 2003; Mukherjee et al., 2009), incubated for 30 min at 37°C under shaking conditions in the dark, and used for flow cytometry, with at least 10000 cells were gated from each sample. The samples were processed similarly in the presence of 5 μM thiourea at the concentration that was found to be non-lethal for 10^4^ cells/ml (Nair et al., 2019). Unstained cells, processed like the test samples, were used as autofluorescence control. Data were acquired using Becton Dickinson FACS Verse flow cytometer with a 488 nm solid state laser and a 527/32 nm emission filter (GFP) at medium flow rate and analyzed using FACSuite software. Statistical significance of the values between the time points was calculated.

### Determination of H_2_O_2_ Levels in Cell Lysate Using Amplex Red Assay

Equal aliquots of *Msm/Mtb* SCF and NCF cells were lysed by sonication, an aliquot was taken for cfu determination to check the lysis efficiency which was found to be 99.9% consistently. The lysed samples were then filtered through 3 kDa cut off spin-filter to remove high molecular weight proteins. Simultaneously, the standards for Amplex Red assay using H_2_O_2_ (Invitrogen; Mohanty et al., 1997; Zhou et al., 1997) were prepared from 0.1 to 10 μM. The concentration of H_2_O_2_ in the tube was freshly determined every time before making up the working solution of H_2_O_2_ before every experiment. The assay was performed, readings were taken and analyzed using i-control software as per manufacturer’s protocol, and statistical significance was calculated. In the case of DMTU or DPI exposure, the H_2_O_2_ concentration was determined in the same manner, except that the *Msm* cells were grown till MLP in the continuous presence of DMTU or DPI.

### Determination of Superoxide Dismutase (SOD) Activity in SCF and NCF Cell Lysates

The superoxide dismutase (SOD) activity assay was performed in the SCF and NCF cell lysates using SOD Assay kit (Sigma-Aldrich), as per manufacturer’s instructions. This kit based method utilizes a highly water-soluble tetrazolium salt WST-1 [2-(4-Iodophenyl)-3-(4-nitrophenyl)-5-(2,4-disulfophenyl)-2H-tetrazolium, monosodium salt] which, upon reduction with superoxide anion, generates a water-soluble dye formazan. Xanthine oxidase directly converts O_2_ to superoxide which will reduce WST-1 and produces the colored compound formazan. However, the presence of SOD will inhibit this reaction by utilizing the superoxide and converting it to H_2_O_2_ and O_2_. This will reduce the formation of formazan and hence less color will be developed. Therefore, the reduction in the formazan color formation is directly correlated with the SOD activity and is represented as percentage inhibition.

The cell pellets obtained were finally resuspended in 650 μl of buffer solution, an aliquot of 20 μl was taken for determining cfu. The lysis of cells was carried out by sonication at 30% amplitude, 1 sec pulse on, 1 sec pulse off for 2 min and repeated twice. The lysed samples were centrifuged at ∼6800 × *g* for 10 min at 4°C to remove debris and supernatant was collected in a fresh tube. From each of SCF and NCF samples 210 μl was taken into two wells of transparent 96 well plate and 10 μl of WST solution with 20 μl enzyme working solution was added. Blank1 was prepared by adding 190 μl of buffer solution, 20 μl of dd H_2_0, 10 μl of WST solution and 20 μl enzyme working solution. Blank2 was prepared with the SCF and NCF cell lysates where 20 μl of dilution buffer was added instead of enzyme working solution. Blank3 was prepared as Blank1 without enzyme working solution and 20 μl of dilution buffer was added. A reaction was set up to be used as a control, where 210 μl of SCF and NCF cell lysate was added to two different wells and 5 mM sodium azide (Roberts and Hirst, 1996) was added into both in order to inactivate the SOD enzyme. All the samples were mixed thoroughly using pipette and kept for incubation at 37°C for 20 min. Absorbance was read at 450 nm using Tecan plate reader. The activity was calculated as per the given formula: SOD activity (inhibition rate%) = {[(Ablank 1 – Ablank 3) – (Asample – Ablank 2)]/(Ablank 1 – Ablank 3)} × 100. The activity was normalized per cell for SCF and NCF samples as well as for the samples containing the SOD inhibitor sodium azide. Statistical significance was calculated using Students’ *t*-test.

### Imaging of *Msm*/pAKMN2-P_*MSMEG*_*6603*_-*ugfp*_*m*_^2+^

*Msm*/pAKMN2-P_*MSMEG*_*6603*_-*ugfp*_*m*_^2+^ cells were grown till MLP and 1 ml of the cells were centrifuged at ∼3900 × *g* for 10 min at room temperature. The cell pellet was resuspended in 200 μl of fresh Middlebrook 7H9 medium. Simultaneously, 1.9% low melting point agarose was used to make agarose pad, as described (Vijay et al., 2017). The cells were layered on the solidified agarose pad, covered with cover glass and incubated for 10 min at 37°C for the cells to adhere to the agarose pad. Following incubation, the *Msm*/pAKMN2-P_*MSMEG*_*6603*_-*ugfp*_*m*_^2+^ cells were observed under Zeiss AxioVision Imager M1 using DIC as well as fluorescence (GFP) channels. The fluorescence intensities of the SCs and NCs were quantitated using ImageJ software. The area of the cell was calculated using ImageJ software and the fluorescence intensities were normalized per unit area of the cell. Statistical significance was calculated using paired two-tailed *t*-test.

### Determination of NADH Oxidase Activity in SCF and NCF Cell Lysates

NADH oxidase is a membrane-bound enzyme which converts molecular oxygen to superoxide in the presence of NADH. The NADH in turn gets converted to NAD^+^ and H^+^. In order to measure the activity of NADH oxidase enzyme, the rate of NADH utilization was calculated by measuring the reduction in the absorbance of NADH over time. Since NADH is also consumed and produced by many other cellular components, to obtain specificity of the assay, a parallel reaction containing the inhibitor of NADH oxidase, diphenyleneiodonium (DPI), was set up. The difference in the absorbance of these two reactions will specifically show the NADH oxidase activity. The SCF and NCF cells were suspended in 200 μl of 50 mM Tris–HCl buffer (pH 8), and an aliquot of 20 μl was taken for determining cfu. The cells were lysed by sonication and 20 μl aliquot of the lysate supernatant was used for protein estimation with Bradford assay. The rest of the sample was used for NADH oxidase assay, as described in detail in section “Supplementary Materials and Methods” in the Supplementary Material. A decrease in the NADH fluorescence at 340 nm during an interval of 30 min was calculated to obtain the activity of NADH oxidase in the SCF and NCF cell lysates. Similar protocol was followed with the lysates of SCF and NCF cells fractionated from *Msm* cultures grown in the presence of 100 nM DPI (NADH oxidase inhibitor) as well. Data was plotted as NADH oxidase activity per cell for SCF and NCF samples. Statistical significance was calculated using Students’ *t*-test.

### Determination of Superoxide Levels in *Msm* SCF and NCF Cells Using Dihydroethidium (DHE) Assay

The detection and quantitation of superoxide levels were performed using dihydroethidium (DHE, Sigma) fluorescence assay, as described (Peshavariya et al., 2007; Nazarewicz et al., 2013; Yeware et al., 2017). The superoxide levels were quantitated in the *Msm* SCF and NCF cells in the presence of diethylenetriaminepentaacetic acid (DTPA, Sigma), which chelates away metal ions that would otherwise destroy superoxide (Fisher et al., 2004; Yeware et al., 2017). TEMPOL (Sigma), a SOD mimic (Yeware et al., 2017), was used as the control. Firstly, the blank values were subtracted from the sample readings. Subsequently, the values obtained in the TEMPOL exposed samples were subtracted from the unexposed samples to determine the relative 2-OH-ethidium levels, which was formed by the oxidation of DHE. This protocol was followed in order to obtain the fluorescence intensity of 2-OH-ethidium specifically from superoxide radical and not from other non-specific ROS sources. Statistical significance was calculated using Students’ *t*-test.

### Determination of Labile and Total Fe^2+^ Levels in *Msm/Mtb* SCF and NCF Cell Lysates

The lysates of *Msm/Mtb* SCF and NCF cells were used for the estimation of labile Fe^2+^ and total Fe^2+^ levels. The lysates were prepared in 100 mM sodium acetate buffer (pH 5.2) in order to maintain acidic environment to stabilize the labile iron in the ferrous form. While one half of the lysate was used for the estimation of labile Fe^2+^ levels, the other half was used to estimate total Fe^2+^ by heating the lysate for 90 min at 90°C, as described (modified protocol from Morones-Ramirez et al., 2013). The estimation was performed using FeRhoNox^TM^-1 (10 μM final concentration, Goryo Chemicals), as described (Hirayama et al., 2013; Tsugawa et al., 2015). In parallel, the ferrous ammonium sulfate, ranging from 0.25 to 16 μM in 100 mM sodium acetate (pH 5.2), were used as the standards. In parallel, another set of labile Fe^2+^ samples were exposed to 100 μM 2,2′-bipyridyl, a Fe^2+^ ion chelator (Farhana et al., 2008) in order to confirm the specificity of FeRhoNox^TM^-1 reaction with Fe^2+^. FeRhoNox^TM^-1 on interaction with Fe^2+^ generates the strongly fluorescing Rhodamine B. The readings of the samples were then taken at 540/575 nm in microplate reader (TECAN infinite 200 pro) and analyzed using i-control software. Statistical significance was calculated using paired two-tailed *t*-test. The lysates of SCF and NCF cells fractionated from *Msm* MLP cells, which were grown in the presence of 1 mM DMTU (H_2_O_2_ scavenger) or 100 nM DPI (NADH oxidase inhibitor) till MLP, were also assayed for labile Fe^2+^ levels using FeRhoNox^TM^-1 assay. Statistical significance was calculated using Students’ *t*-test.

### Imaging of FeRhoNox^TM^-1 Stained *Msm* MLP Cells

*Msm* MLP cells were stained with 10 μM FeRhoNox^TM^-1 (final concentration) and kept for incubation at 37°C for 1 hr under shaking condition. Subsequently, the cells were harvested and prepared for live cell imaging as described under “Imaging of *Msm*/*pAKMN2-Pmsmeg_6603*-*ugfp_*m*_^2+^*.” The stained *Msm* cells were observed under Zeiss AxioVision Imager M1 using DIC as well as fluorescence channel (rhodamine). The fluorescence intensities of the SCs and NCs were quantitated using ImageJ software. The area of the cell was calculated using ImageJ software and the fluorescence intensities were normalized per unit area of the cell. Statistical significance was calculated using paired two-tailed *t*-test.

### Determination of Aconitase Activity in SCF and NCF Cell Lysates

Aconitase drives an enzymatic reaction where conversion of isocitrate to aconitate takes place, which is a part of citric acid cycle. The activity of the enzyme can be calculated by measuring the absorbance of aconitate at 240 nm. The increase in the absorbance represents the activity of the enzyme and decrease in the absorbance will show the inactivity/inefficiency of the enzyme aconitase. The cell lysate showing higher rate of aconitate production reflects the higher activity of aconitase enzyme. The concentration of aconitate was calculated using its millimolar extinction coefficient 3.6 mM^–1^ cm^–1^ (Kennedy et al., 1983). Aconitase activity was determined in the lysates of *Msm* SCF and NCF cells, as described (Pechter et al., 2013). The cells were lysed with lysozyme (chicken egg white, Fluka) and lipase (*Candida cylindracea*, Sigma). *Sonication was not used for the cell lysis in order to avoid the inactivation of aconitase in the samples* (Pechter et al., 2013). An aliquot each of the cells was taken for determining cfu before lysozyme-lipase digestion. The assay was performed using the substrate, DL-isocitric acid trisodium salt hydrate. The absorbance of the samples was taken at 240 nm every 1 min for 5 min and the concentration of aconitate was calculated using its millimolar extinction coefficient 3.6 mM^–1^ cm^–1^ (Kennedy et al., 1983). Subsequently, the aconitase activity of the samples was represented as units per cell (Pechter et al., 2013). Further, for the negative control samples, 300 μM EDTA was added to the cell suspension (to inactivate aconitase) before the lysozyme-lipase digestion (Varghese et al., 2003). Likewise, for the positive control samples, the lysates prepared from the cells grown in the presence of 1 mM DMTU (H_2_O_2_ scavenger) or 100 nM DPI (NADH oxidase inhibitor) were used for the assay. Statistical significance was calculated using paired two-tailed *t*-test.

### Resister Generation Frequency of SCF and NCF Against Rifampicin and Moxifloxacin

An aliquot each of SCF and NCF cell suspensions were used to determine cfu. The rest of the cells (10^8^ cells/ml) were entirely plated on 125 μg/ml of rifampicin (3× MBC) or 0.5 μg/ml of moxifloxacin (3.75× MBC). The number of resistant mutants on the antibiotic containing plate was divided by the total number of bacterial cells, determined from the antibiotic-free plates, to obtain the resister generation frequency for each sample against the respective antibiotic. The resister generation frequency of the samples was also calculated in the same manner for the cells grown in the presence of 1 mM DMTU (H_2_O_2_ scavenger), 100 nM DPI (NADH oxidase inhibitor) and 0.5 mM thiourea (hydroxyl radical scavenger). Statistical significance was calculated using paired two-tailed *t*-test.

### Preparation of cfu-Based v/v/v Mixtures of *Msm* SCF1, SCF2, and NCF

Based on cfu analysis, a v/v/v ratio of 1:1:5 mixture of SCF1:SCF2:NCF cells was found to give their proportion similar to that exists in MLP, which was called Natural-Like Proportion (NLP). For the preparation of Un-natural Proportion 1, UNP1 mixture, the SCF1, SCF2, and NCF were mixed back at the 1:1:1 v/v/v proportion at which they do not exist. Similarly, the UNP2 (1:1:2 v/v/v) and UNP3 (2:2:1 v/v/v) mixtures were prepared. The Total Reconstituted Population (TRP) was prepared by mixing equal volumes (100 μl each) of the cells from all the nine (64, 66, 68, 70, 72, 74, 76, 78, and 80%) Percoll fractions. The cfu of each of the samples was determined by plating (*n* = 10; Supplementary Table S2). Equal cell density (10^3^ or 10^4^ cells/ml) of the respective *Msm* SCF1, SCF2, NCF, UNP2, UNP3 cells, and/or NLP, UNP1, TRP, and MLP cells were exposed to 25 μg/ml rifampicin for 4 h (Vijay et al., 2017) individually or combinedly (SCF1 and SCF2), as the case may be. The percentage survival of the different samples, in terms of cfu, against antibiotic was determined by plating the respective antibiotic-stressed cells and the unstressed cells on antibiotic-free plates.

### Resister Generation Frequency Determination of SCF, NCF, and NLP Against Rifampicin

An aliquot each of SCF and NCF cells and NLP mixture was taken for cfu determination. The remaining cells were entirely plated on 125 μg/ml of rifampicin. The resister generation frequency was calculated for each sample by dividing the number of resistant mutants on the antibiotic containing plate with the total number of bacterial cells, determined from antibiotic-free plates. In the case of DMTU exposure, the resister generation frequency of the SCF, NCF, and NLP samples prepared from the *Msm* cells grown in the presence of 1 mM DMTU till MLP were also determined in similar manner. The resister generation frequency of NLP cells, which were prepared by v/v/v mixing SCF cells obtained from DMTU (H_2_O_2_ scavenger)-exposed culture and NCF cells from unexposed culture, was also determined. Statistical significance was calculated using paired two-tailed *t*-test.

### Calculation of Expected Rifampicin Resisters From SCF and NCF Individually in NLP Mixture and From NLP Cells *per se*

Since the proportion of SCF and NCF cells in the NLP mixture is known, using the individual resister generation frequency of the SCF and NCF cells, the expected resister generation frequency of NLP was calculated, as per the details given in section “Supplementary Materials and Methods” in the Supplementary Material.

### Mutation Analyses in *Msm* SCF, NCF, and NLP Resisters Against Rifampicin and Moxifloxacin

The RRDR locus of the *rpoB* gene was amplified using the genomic DNA samples of rifampicin-resistant mutants from SCF, NCF and NLP, and specific primers (Supplementary Table S3). The QRDR locus of the *gyrA* gene was amplified using QRDR-specific primers (Supplementary Table S3) with the genomic DNA samples of MXF-resistant mutants as template from SCF and NCF. The nucleotide sequence of the PCR products was determined on both the strands. An authentic mutation was considered when a mutation appeared at the same nucleotide position in both the forward and reverse sequencing reactions of the mutants.

### Determination of H_2_O_2_ Levels Secreted From Cells Using Amplex Red Assay

An aliquot each of SCF and NCF cells was used for cfu determination. The remaining samples were pelleted down, resuspended in 1500 μl of 1× PBS and from this 50 μl of the cell suspension was taken for the assay of secreted H_2_O_2_, in the Amplex Red assay, using the Amplex Red Hydrogen Peroxide/Peroxidase assay kit (Invitrogen), as described (Mohanty et al., 1997; Zhou et al., 1997) and as per manufacturer’s protocol. Different concentration of freshly prepared H_2_O_2_ were used as standards. After incubation in the dark at room temperature for 30 min, the readings were taken at 530/590 nm in microplate reader (TECAN infinite 200 pro) and analyzed using i-control software. In the case of DMTU (H_2_O_2_ scavenger) exposure, the entire protocol was performed similarly, except that the SCF and NCF cells were prepared from the *Msm* cells grown in the presence of 1 mM DMTU till MLP. Statistical significance was calculated using paired two-tailed *t*-test.

### Total RNA Preparation From Unexposed *Msm* SCF and NCF Cells

Total RNA from the SCF and NCF cells were isolated using hot phenol method, as described (Wecker, 1959; Ausubel and Kingston, 1987) with slight modifications for mycobacterial cultures, which is described in section “Supplementary Materials and Methods” in the Supplementary Material. The final RNA pellet was then air-dried at room temperature for 15 min and dissolved in 20 μl of DEPC-treated water. The SCF1, SCF2 and NCF RNA samples were quantitated using NanoDrop^TM^ 1000 Spectrophotometer (Thermo Fisher Scientific) and stored at −70°C.

### Preparation of cDNA for Real-Time PCR

For the preparation of cDNA from each gene, 100 ng of total RNA was used from each of the three populations, SCF1, SCF2, and NCF. The cDNA synthesis reaction was carried out with the following constituents: 10 pmoles of gene-specific reverse primer (Sigma), 50 μM dNTP mix (Thermo Fisher Scientific), 20 units of RiboLock RNase inhibitor (Thermo Fisher Scientific), 200 units of RevertAid-Premium Reverse Transcriptase (RNaseH minus, thermostable, Thermo Fisher Scientific), and 1× RT buffer. The final volume of the reaction was made up to 20 μl by adding double-distilled autoclaved water. The cDNA synthesis was carried out using the following conditions: denaturation at 65°C for 5 min, followed by annealing and extension combinedly for 30 min at 56°C and inactivation of enzyme at 85°C for 10 min. The cDNA thus obtained was stored at −20°C till real time PCR was performed. cDNA for 16S rRNA from the same sample was used as the normalization control.

### Real-Time PCR

Real-Time PCR was performed using Real Time PCR EvaGreen Mastermix (G-Biosciences), as per manufacturer’s instructions, and the experiments were analyzed according to comparative ΔΔCt method (Wang et al., 2006) followed by normalization (according to Willems et al., 2008). In order to check the specificity of the primers (Supplementary Table S4) and formation of specific product, melt curves were prepared. The real-time PCR experiment was performed using the Comparative Ct (ΔΔCt) method with CFX96 Real-Time system from Biorad. The fold-change in the expression levels of mRNAs from *Msm* SCF1 and SCF2 cells with respect to that from the NCF cells was calculated. Statistical significance was calculated using Students’ *t*-test.

### Promoter Cloning of *MSMEG*_*6603* and Genome Integration to Drive Reporter Gene

The NADH oxidase gene (*MSMEG*_*6603*) sequence was analyzed and 275 bp region upstream of the gene was selected as the promoter sequence. This 275 bp sequence was amplified using High fidelity Phusion DNA polymerase (Thermo Fisher Scientific) from the *Msm* genome with Msm-6603-P1-f and Msm-6603-P1-r specific primers with *Xba*I and *Eco*RV restriction sites, respectively (Supplementary Table S5). The amplified sequence was first cloned in pBS(KS) and sequence verified. The promoter segment was then subcloned in pAKMN2-*ugfp_*m*_^2+^* vector as 5′ transcriptional fusion to the reporter gene *ugfp_*m*_^2+^* (Roy et al., 2012; Sebastian, 2016). *Msm* cells were electroporated with the recombinant pAKMN2-P_MSMEG___6603_-*ugfp_*m*_^2+^* vector. The clones were confirmed by PCR amplification with Taq DNA polymerase (Thermo Fisher Scientific) using mycgfp2-RT-f and mycgfp2-RT-r primers (Supplementary Table S4). The *Msm*/pAKMN2-P_MSMEG___6603_-*ugfp_*m*_^2+^* integrant cells were cultured to MLP multiple times serially in the absence of hygromycin and then re-cultured in the presence of hygromycin (50 μg/ml) to ensure the stability of the integrant.

### Genomic DNA Isolation From *Msm* SCF, NCF, and NLP Resisters

Genomic DNA was isolated from *Msm* SCF, NCF, and NLP resisters, as described (Ausubel and Kingston, 1987), but with modifications described in section “Supplementary Materials and Methods” in the Supplementary Material. The final genomic DNA pellet was dissolved in 20 μl Tris–HCl-EDTA buffer (10 mM Tris–HCl and 1 mM EDTA, pH 8.0) and stored at 4°C.

## Results

### Experimental Strategy

All the experiments were performed using the SCs-enriched fraction (SCF) and the NCs-enriched fraction (NCF) of the cells, which were isolated from the actively growing mid-log phase (MLP) cultures of *Msm* and *Mtb in vitro*. We had earlier shown that the SCF and the NCF cells of *Msm* or *Mtb*, upon incubation in fresh medium, would grow and divide within their respective division time of 3 h (*Msm*) or 24 h (*Mtb*) to give a complete normal mid-log phase like population (Vijay et al., 2014a,b, 2017). Therefore, the *Msm*/*Mtb* SCF and NCF cells, fractionated from the respective mid-log phase cultures, were immediately taken for analyses without any incubation in every experiment. Further, we had earlier shown that Percoll fractionation *per se* of the MLP culture into the SCF and NCF cells did not have any effect on the viability of the cells from these subpopulations (Vijay et al., 2017). The freshly fractionated SCF and NCF cells were analyzed to determine their redox status using dyes specific for superoxide, H_2_O_2_, and hydroxyl radical. The redox status of the cells was qualitatively and quantitatively determined by measuring the levels of fluorescence from the dye-stained cells. As the negative controls, the SCF and NCF cells, which were fractionated from the cultures grown in the continuous presence of non-toxic concentrations of specific inhibitors of the respective ROS, were also stained with the specific dyes and fluorescence measured. In parallel, the levels of labile Fe^2+^ and total iron, by converting into the Fe^2+^ form, were also quantitated using Fe^2+^-specific dye. Further, the molecular reasons for the differential levels of ROS and labile Fe^2+^ were identified by determining the activities of specific redox enzyme systems and by measuring the levels of expression of specific redox and antioxidant gene systems. The physiological benefit of the significant differential oxidative status between the SCF and the NCF cells was determined in terms of the significant difference in their antibiotic resister generation frequency. Based on all the findings, a model was proposed to depict the inherent significant difference in the metabolic design of the SCF and the NCF cells that conferred differential redox status and thereby differential resister generation frequency on them with obvious physiological benefits.

### *Msm* SCF Cells Have Significantly Higher Oxidative Status Than NCF Cells

**FIGURE 1:**
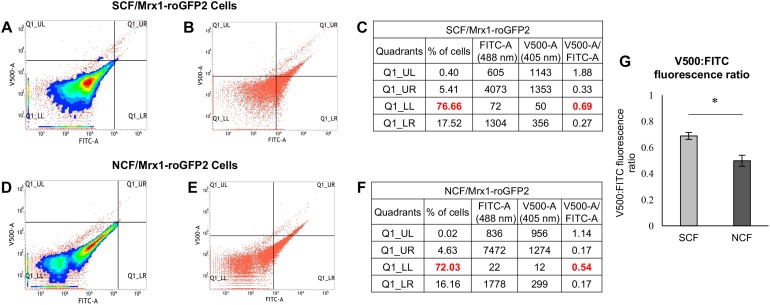
Determination of the oxidative status and ROS (hydroxyl radical and H_2_O_2_) levels in the unexposed freshly prepared SCF/Mrx1-roGFP2 and NCF/Mrx1-roGFP2 cells. Flow cytometry profile of roGFP2 fluorescence of: **(A)** SCF/Mrx1-roGFP2 cells, **(D)** NCF/Mrx1-roGFP2 cells. **(B,E)** Scatter plot of roGFP2 fluorescence of the respective cells from the data in **(A,D)**, respectively. **(C,F)** Percentages of the respective cells in each quadrant with their V500 and FITC median fluorescence values and their ratios. The major proportion of the cells amongst the four quadrants and their fluorescence ratio are given in bold red color. **(G)** Quantitation of V500:FITC fluorescence ratio in the SCF/Mrx1-roGFP2 and NCF/Mrx1-roGFP2 cells (*n* = 3). Statistical significance was calculated using paired *t*-test where ^*^Indicates *p* ≤ 0.05.

We determined the ratio of the V500:FITC median fluorescence of the SCF and the NCF subpopulations individually to determine the difference in their oxidative status. Further, to find out whether there is heterogeneity in the endogenous ROS levels within the individual subpopulations, we also determined the ratio of the V500:FITC median fluorescence of the minor subpopulations within each of the individual subpopulations through scatter plot analysis of the fluorescence. A high V500:FITC fluorescence ratio is indicative of an increase in V500 fluorescence and/or a decrease in FITC fluorescence showing high oxidative status. The SCF/Mrx1-roGFP2 cells, which were fractionated from *Msm* MLP cultures carrying genome-integrated redox biosensor Mrx1-roGFP2 (Bhaskar et al., 2014; Nair et al., 2019), showed significantly higher V500:FITC fluorescence ratio than the NCF/Mrx1-roGFP2 cells (Figures 1A–C and D–F, respectively, and G). Thus, the high V500:FITC fluorescence ratio of SCF/Mrx1-roGFP2 cells revealed the inherently high and statistically significant oxidative status of the SCF cells as compared to the NCF cells. Further, it was of interest to note that the SCF and the NCF subpopulations of cells were constituted by further minor subpopulations that differed in their oxidative status (Figures 1B,C and E,F, respectively). The differences in the ROS levels of these minor subpopulations indicated the high level of heterogeneity in the oxidative status of the SCF and the NCF cells. Nevertheless, the clear conclusion was that the endogenous oxidative status of the SCF cells was significantly higher than that of the NCF cells and that it was contributed by multiple minor subpopulations having different extents of ROS levels.

### Significantly High Levels of Hydroxyl Radical in *Msm* SCF Cells

**FIGURE 2:**
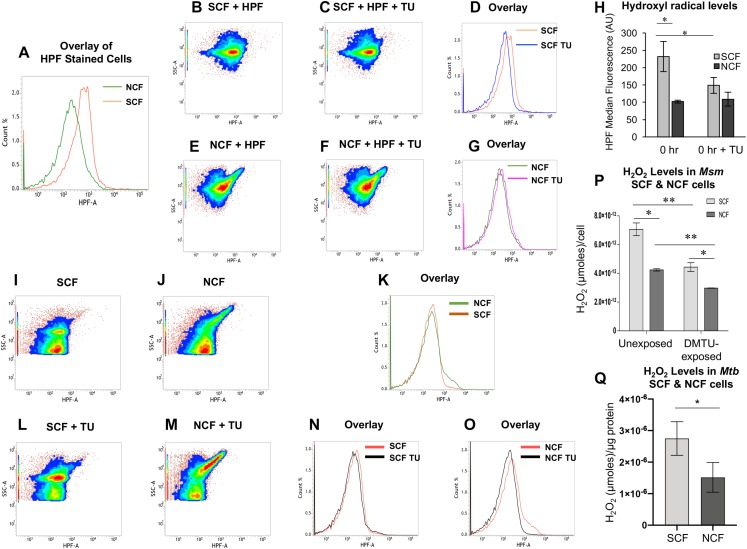
The HPF fluorescence flow cytometry profile of *Msm* SCF and NCF cells and the ROS levels in the *Msm* and *Mtb* SCF and NCF cells. **(A)** Histogram overlay of the median fluorescence of HPF-stained *Msm* SCF and NCF cells. **(B,E)** and **(C,F)** Density plots of HPF-stained: *Msm* SCF and NCF cells in the **(B,E)** absence and **(C,F)** presence of non-toxic concentration of thiourea (5 μM, Nair et al., 2019), respectively. **(D,G)** Histogram overlay of median fluorescence in the absence and presence of thiourea for the HPF-stained *Msm* **(D)** SCF and **(G)** NCF cells. **(H)** Quantitation of the median fluorescence, indicating hydroxyl radical levels, in the HPF-stained *Msm* SCF and NCF cells after normalization with their respective autofluorescence samples from **(I–O)** (*n* = 3). Statistical significance was calculated using paired *t*-test where ^*^Indicates *p* ≤ 0.05. **(I,J)** Flow cytometry profile of autofluorescence of unstained 10^4^ cells/ml of *Msm* **(I)** SCF, and **(J)** NCF cells. **(K)** Histogram overlay of the unstained SCF and NCF cells. **(L,M)** Autofluorescence of unstained control cells of: **(L)** SCF and **(M)** NCF in the presence of thiourea (TU). **(N,O)** Histogram overlay of unstained **(N)** SCF cells (from **I** and **L**) and **(O)** NCF cells (from **J** and **M**) in the absence and presence of TU, respectively. **(P)** Quantitation of the levels of H_2_O_2_ per cell in the unexposed and DMTU-exposed non-toxic concentration of DMTU (1 mM; Supplementary Figures S1A–C) *Msm* SCF and NCF cells using Amplex Red assay (*n* = 3). **(Q)** Quantitation of the levels of H_2_O_2_ per μg protein in the *Mtb* SCF and NCF cells using Amplex Red assay (*n* = 3). The values of statistical significance in **(P)** and **(Q)** were calculated using paired *t*-test where ^*^ and ^∗∗^ indicate *p* ≤ 0.05 and *p* ≤ 0.01, respectively.

Since hydroxyl radical is one of the major contributors to oxidative stress in the antibiotics-exposed bacterial systems (Kohanski et al., 2007; Grant et al., 2012; Piccaro et al., 2014; Sebastian et al., 2017), we wanted to find out whether the high levels of ROS in the native SCF cells was due to hydroxyl radical. For this purpose, the freshly isolated native SCF and NCF cells were stained with hydroxyl radical specific fluorochrome, 3′-(p-hydroxyphenyl) fluorescein (HPF, 5 μM; Setsukinai et al., 2003; Mukherjee et al., 2009). Significantly higher HPF fluorescence was found in the SCF cells than in the NCF cells (Figures 2A,B,E,H, and I–K for autofluorescence controls). Measurement of HPF fluorescence in the presence of non-toxic concentration of the hydroxyl radical specific quencher, thiourea (5 μM for 10^4^ cells/ml; Nair et al., 2019), showed significant reduction in the fluorescence (Figures 2C,D,F,G,H, and L–O for autofluorescence controls). It confirmed that the HPF fluorescence, which was detected and quantitated, was specifically due to the presence of hydroxyl radical. Thus, the native SCF cells inherently generated significantly higher levels of hydroxyl radical than the NCF cells. The inherently higher levels of hydroxyl radical in the SCF cells might have been one of the contributors to the naturally elevated levels of oxidative status of the SCF cells.

### *Msm* and *Mtb* SCF Cells Have Significantly High Levels of Intracellular H_2_O_2_

Since hydroxyl radical is majorly produced by Fenton reaction involving H_2_O_2_ and labile Fe^2+^ (Winterbourn, 1995), the higher hydroxyl radical levels in the SCF cells implied higher levels of H_2_O_2_ and labile Fe^2+^ in them, as compared to that in the NCF cells. Confirming this implication, the *Msm* SCF cell lysate contained significantly higher levels of H_2_O_2_ per cell than the *Msm* NCF cell lysate, as shown by the amplex red fluorochrome assay (Figure 2P). The lysates of the SCF and the NCF cells, which were fractionated from the *Msm* cells cultured upto the MLP in the presence of non-toxic concentrations of the H_2_O_2_ scavenger, 1,3-dimethyl-2-thiourea (DMTU; 1 mM for 10^8^ cells/ml) (Parker et al., 1985), showed significant reduction in the H_2_O_2_ levels per cell (Figure 2P and Supplementary Figures S1A–C). The experiment using DMTU-treated cells showed the specific synthesis of H_2_O_2_ by the cells and of the specificity of detection and quantitation of H_2_O_2_. These observations confirmed that SCF cells in the actively growing population, inherently produced significantly higher levels of H_2_O_2_, as compared to the NCF cells. This strongly supported the possibility that the higher hydroxyl radical levels in the SCF cells might have been due to the presence of higher levels of intracellular H_2_O_2_ in the cells as compared to those in the NCF cells.

Like the *Msm* SCF cells, the *Mtb* SCF cells also contained significantly higher levels of intracellular H_2_O_2_ per μg protein in the lysate as compared to the *Mtb* NCF cells (Figure 2Q). Thus, despite *Msm* being a saprophyte and *Mtb* being a virulent pathogen, the SCF cells of both the species in the actively growing cultures inherently generated significantly higher levels of H_2_O_2_ than the respective NCF cells. This indicated that the generation of significantly higher levels of H_2_O_2_ by the SCF cells is an inherent characteristic of the mycobacterium genus irrespective of the nature of the species and that the pathogenicity or virulence status of the bacterium does not have a role in it.

### Significantly High Levels of Superoxide in *Msm* SCF Cells

**FIGURE 3:**
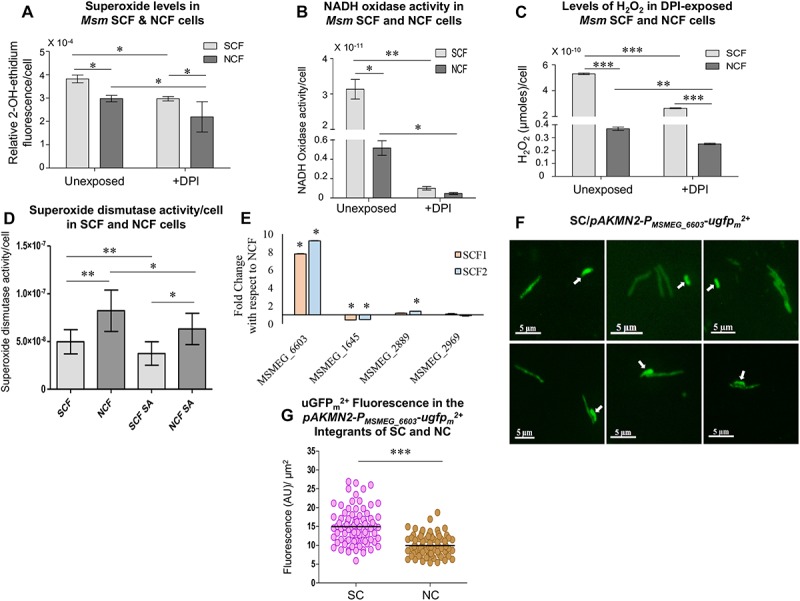
Superoxide and H_2_O_2_ levels in SCF and NCF cells and analysis of NADH oxidase expression and activity. **(A)** Relative levels of 2-OH-ethidium fluorescence in SCF and NCF cells from unexposed and DPI-exposed conditions (*n* = 3). **(B)** NADH oxidase activity in SCF and NCF cells from unexposed and DPI-exposed conditions (*n* = 3). **(C)** Quantitation of H_2_O_2_ levels in SCF and NCF cells from unexposed and DPI-exposed conditions, using Amplex Red assay (*n* = 3 technical triplicates). **(D)** Superoxide dismutase (SOD) activity in SCF and NCF cells in the absence and presence of SOD inhibitor sodium azide (*n* = 6). **(E)** Fold change in the expression of NADH oxidase genes of SCF cells in normalized with the expression in NCF cells by qPCR (*n* = 2). **(F)** Fluorescence microscopy images of *Msm*/pAKMN2-P_*MSMEG*_____6603_-u*gfp_*m*_^2+^* MLP cells. Arrows indicate SCs. **(G)** Single cell fluorescence intensity analysis of SCs and NCs in MLP [calculated from **(F)**, *n* = 85 cells]. Statistical significance was calculated using Students’ *t*-test for **(A)**, **(B)**, and **(E)** and paired *t*-test for **(C)**, **(D)**, and **(G)** where ^*^, ^∗∗^, and ^∗∗∗^ indicate *p* ≤ 0.05, *p* ≤ 0.01, and *p* ≤ 0.001, respectively.

Formation of H_2_O_2_ occurs mostly by the dismutation of superoxide anion (O_2_^∙⁣–^) (McCord and Fridovich, 1968, 1969; González-Flecha and Demple, 1995). Therefore, the significantly high levels of H_2_O_2_ in the SCF cells implied that the cells might be having high levels of superoxide as compared to the NCF cells. Consistent with the high levels of H_2_O_2_ in the SCF cells, the native SCF cells contained significantly high levels of superoxide per cell, as compared to the NCF cells, when measured using dihydroethidium fluorescence assay (Figure 3A; Peshavariya et al., 2007; Nazarewicz et al., 2013; Yeware et al., 2017). It is known that NADH oxidase activity majorly contributes to superoxide generation by the transfer of electron to molecular oxygen (Badwey and Karnovsky, 1979). Therefore, the SCF and the NCF cells fractionated from the *Msm* cells cultured in the presence of an NADH oxidase inhibitor should show reduced levels of superoxide. Hence we determined superoxide levels in the SCF and the NCF cells, which were fractionated from the *Msm* cells cultured in the continuous presence of non-toxic concentration of the cell-permeable NADH oxidase inhibitor, DPI chloride (DPI; 100 nM for 10^8^ cells/ml; Supplementary Figures S1D–F; Li and Trush, 1998; Yeware et al., 2017). The SCF cells, which were fractionated from the DPI-exposed *Msm* culture, showed significant reduction in the superoxide levels per cell, as compared to the SCF cells fractionated from the DPI-unexposed culture (Figure 3A). The NCF cells, which were fractionated from the DPI-exposed culture, also showed a significant reduction in the superoxide levels per cell, as compared to the NCF cells prepared from the unexposed culture (Figure 3A). These observations confirmed that the elevated levels of superoxide anion in the SCF cells were certainly one of the contributors to the significantly high levels of H_2_O_2_ in them.

### *Msm* SCF Cells Have Significantly High Levels of NADH Oxidase Activity

The significantly high levels of superoxide in the SCF cells and the reduction in the superoxide levels in the NADH oxidase inhibitor (DPI)-exposed SCF cells implied the possibility of high levels of NADH oxidase activity in them. Confirming this implication, the lysate prepared from the SCF cells, which were fractionated from the *Msm* MLP cultures, showed significantly elevated levels of NADH oxidase activity per cell, as compared to the equivalent NCF cell lysate (Figure 3B). On the contrary, the lysate prepared from the SCF cells, which were fractionated from the *Msm* cells cultured in the continuous presence of non-toxic concentration of DPI, showed drastic reduction in the NADH oxidase activity (Figure 3B). Equivalent NCF cell lysate also showed significant reduction in the NADH oxidase activity (Figure 3B). The DPI-dependent specific reduction in the NADH oxidase activity confirmed that the inherent elevated levels of superoxide anion in the SCF cells were due to the high levels of NADH oxidase activity in them.

Since this is the case with the high levels of superoxide generation and since H_2_O_2_ is formed majorly through the dismutation of superoxide anion (O_2_^∙⁣–^) (McCord and Fridovich, 1968, 1969; González-Flecha and Demple, 1995), DPI-mediated inhibition of NADH oxidase activity should bring down superoxide levels and consequentially H_2_O_2_ levels as well. Consistent with this expectation, the SCF cells fractionated from the *Msm* cells cultured in the continuous presence of non-toxic concentration of DPI showed significantly reduced levels of H_2_O_2_, as compared to the H_2_O_2_ levels in the SCF cells fractionated from the DPI-unexposed culture (Figure 3C). The DPI-exposed NCF cells also showed significant reduction in the H_2_O_2_ levels, as compared to the unexposed cells (Figure 3C). These experiments confirmed that the significantly high levels of H_2_O_2_ in the SCF cells were due to the elevated levels of NADH oxidase activity that produced significantly high levels of superoxide which in turn got converted to H_2_O_2_.

### Significantly High Levels of Superoxide Dismutase Activity in *Msm* SCF Cells

The significantly high levels of H_2_O_2_ in the SCF cells and their drastic reduction upon inhibition of NADH oxidase activity, and therefore inhibition of superoxide production, indicated the possibility of high levels of superoxide dismutase (SOD) activity in the cells. Confirming this possibility, both the SCF and the NCF cell lysates showed significantly high levels of SOD activity per cell (Figure 3D). The SOD activity was significantly reduced in the presence of 5 mM sodium azide, which is an inhibitor of the enzyme (Roberts and Hirst, 1996). It was of interest to note that the SCF cells showed significantly lesser SOD activity than the NCF cells (see section “Discussion”). All these observations revealed the inherent significantly highly active pathway in the SCF cells in which high levels of NADH oxidase activity produced significantly high levels of superoxide, which in turn were converted to high levels of H_2_O_2_ by the high levels of SOD activity.

### *Msm* SCF Cells Show Significantly High Levels of NADH Oxidase Expression

Inherently higher levels of the activity of NADH oxidase in the SCF cells could be due to the higher levels of the expression of NADH oxidase or enhanced activation of the enzyme in the SCF cells, as compared to that in the NCF cells. However, *Msm* contains four conspicuous NADH:flavin oxidoreductase/NADH oxidases: MSMEG_1645, MSMEG_2889, MSMEG_2969, and MSMEG_6603. Real time PCR analyses showed the expression levels of NADH:flavin oxidoreductase/*nadh* oxidase (MSMEG_6603) to be eight- to nine-fold higher in both the SCF1 and the SCF2 cells, as normalized to their levels in the NCF cells (Figure 3E). The other three NADH:flavin oxidoreductases, MSMEG_1645, MSMEG_2889, and MSMEG_2969, showed expression levels comparable to those of the NCF cells. Thus, the SCF cells showed exclusively high levels of expression of the specific NADH:flavin oxidoreductase/NADH oxidase, MSMEG_6603, among the four NADH:flavin oxidoreductases in *Msm* cells.

For confirming the high expression levels of NADH oxidase (MSMEG_6603), we generated an *Msm*/pAKMN2-P_MSMEG___6603_-u*gfp_*m*_^2+^* strain carrying transcriptional fusion of the specific *nadh* oxidase promoter to mycobacterial codon-optimized unstable *gfp_*m*_^2+^* (*ugfp_*m*_^2+^*) (as a single copy integrated at the L5 mycobacteriophage *att* site in the genome) (Roy et al., 2012; Sebastian, 2016). To determine the uGFP_*m*_^2+^ fluorescence in the pAKMN2-P_MSMEG___6603_-u*gfp_*m*_^2+^* integrants of the SCs and the NCs using fluorescence microscopy, all the individual cells, which were of length ≤ 2.6 μm, were taken as the SCs while the cells of length > 2.6 μm were taken as the NCs. This demarcation was as per our previous findings on the size distribution of subpopulations of cells in mycobacterial cultures (Vijay et al., 2017). Fluorescence imaging of the cells from the MLP culture of *Msm*/pAKMN2-P_MSMEG___6603_-u*gfp_*m*_^2+^* showed significantly high uGFP_*m*_^2+^ fluorescence in the SCs as compared to that in the NCs (Figures 3F,G; *n* = 85 cells). The extent of variation in the promoter activity was more in the SCs than in the NCs, probably indicating stochasticity in the levels of the promoter activity among individual SCs (Figure 3G).

Thus, the inherent remarkably high levels of expression of *nadh* oxidase (MSMEG_6603) in the SCF cells, unlike in the NCF cells, might have contributed to the significantly high superoxide (O_2_^∙⁣–^) levels in them. These experiments unraveled the existence of significant heterogeneity in the *nadh* oxidase expression between the SCs and the NCs subpopulations. The higher extent of variation in the *nadh* oxidase promoter activity in the SCF cells, as compared to that in the NCF cells, implied that the levels of O_2_^∙⁣–^ and consequentially of H_2_O_2_ might also be varying among the individual SCs in the subpopulation, albeit always significantly higher than that in the subpopulation of NCs.

### Contribution of Other Oxidoreductase and Antioxidant Genes to High Oxidative Stress

The observations made so far showed that the root cause for the high levels of oxidative status of the SCF cells was the high levels of O_2_^∙⁣–^ generation by the specific NADH oxidase, MSMEG_6603. However, the high levels of O_2_^∙⁣–^ levels could also be facilitated by: (i) higher expression/activity of respiratory/electron transport chain (ETC) genes (Forman and Kennedy, 1975); (ii) lower expression/activity of antioxidant genes (Van Raamsdonk and Hekimi, 2012); and (iii) autoxidation of non-respiratory flavoproteins (Korshunov and Imlay, 2010). Real-time PCR analyses of a set of such genes indicated the possibility of their contributions.

The expression levels of *nuoH* (NADH-quinone oxidoreductase, H subunit) were three-fold high in the SCF1 cells, but not in the SCF2 cells, while those of the other respiratory/ETC system components were comparable to that of the NCF cells (Supplementary Figures S2A,B). However, the expression levels of many of the antioxidant genes were only one- to two-fold higher in the SCF cells, with *katG* showing ∼two-fold higher levels in the SCF cells (Supplementary Figures S2C,D). Probably, the higher H_2_O_2_ levels in the SCF cells might have induced *katG* unlike in the NCF cells, although *katG* induction is not proportionate to H_2_O_2_ levels (Li X. et al., 2015). The expression levels of the three SOD genes were all one-fold higher in the SCF cells (Supplementary Figures S2C,D). Among the other antioxidant genes, the levels of expression of *gpx* was two-fold higher in the SCF cells (Supplementary Figures S2E,F). This might help in the detoxification of H_2_O_2_ by oxidizing it into water and oxygen (Lubos et al., 2011). The rationale behind the limited levels of expression of the antioxidant genes is presented under the Discussion.

### *Msm* and *Mtb* SCF Cells Have Significantly High Levels of Labile Fe^2+^

**FIGURE 4:**
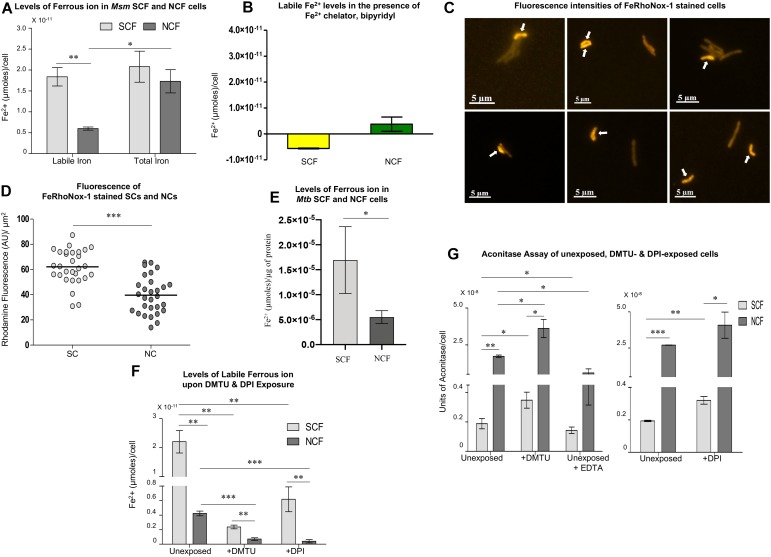
Analysis of iron levels in *Msm* and *Mtb* SCF and NCF cells. **(A)** Estimation of labile and total iron levels (μmoles/cell) in the SCF and NCF cells using FeRhoNox-1 assay (*n* = 3). **(B)** Levels of Fe^2+^ (μmoles/cell) in *Msm* SCF and NCF cell lysates in the presence of 100 μM bipyridyl (*n* = 3). **(C)** Fluorescence microscopy images of FeRhoNox-1 stained MLP cells. Arrows indicate SCs. **(D)** Single cell analysis of the fluorescence intensities of FeRhoNox-1 stained SCs and NCs [calculated from **(C)**, *n* = 29 cells]. **(E)** Estimation of labile iron levels in the *Mtb* SCF and NCF cells using FeRhoNox-1 assay (*n* = 3). **(F)** Estimation of labile iron levels in the unexposed, DMTU-exposed and DPI-exposed *Msm* SCF, and NCF cells using FeRhoNox-1 assay (*n* = 3). **(G)** Determination of aconitase activity in the unexposed, DMTU-exposed and DPI-exposed *Msm* SCF and NCF cells (*n* = 3). Statistical significance was calculated using paired *t*-test for **(A)**, **(D)**, and **(G)**, and Students’ *t*-test for **(E)**, and **(F)** where ^*^, ^∗∗^, ^∗∗∗^ indicate *p* ≤ 0.05, *p* ≤ 0.01, and *p* ≤ 0.001, respectively.

Besides H_2_O_2_, the other component required for the Fenton reaction to produce hydroxyl radical is labile Fe^2+^ ions (Winterbourn, 1995). The significantly high levels of hydroxyl radical in the SCF cells indicated the presence of high levels of labile Fe^2+^ ions (see Figures 2A–H). Therefore, we determined the labile Fe^2+^ levels in the SCF and NCF cells at pH 5.2 (to keep labile iron in the Fe^2+^ form) using Fe^2+^-specific fluorochrome, FeRhoNox^TM^-1 (10 μM final concentration) (Hirayama et al., 2013; Tsugawa et al., 2015). Significantly higher levels of FeRhoNox-1 fluorescence were found in the SCF cells than in the NCF cells, indicating significantly high levels of labile Fe^2+^ in the SCF cells (Figure 4A; *n* = 3). Whereas, the total iron content in the SCF and the NCF cells was comparable (Figure 4A; *n* = 3). Bipyridyl, an Fe^2+^ scavenger (100 μM; Farhana et al., 2008), significantly abolished FeRhoNox^TM^-1 fluorescence in the SCF cells thereby confirming the presence of free Fe^2+^ (Figure 4B; *n* = 3). The abolition of Fe^2+^-specific FeRhoNox^TM^-1 fluorescence by bipyridyl also confirmed the specificity of FeRhoNox^TM^-1 reaction with free Fe^2+^ ions. Imaging of the FeRhoNox^TM^-1-stained *Msm* MLP cells and quantitation of the fluorescence intensities of the FeRhoNox^TM^-1 stained individual SCs and NCs showed significantly high fluorescence in the SCs than in the NCs (Figures 4C,D; *n* = 29 cells). This indicated significantly higher levels of labile Fe^2+^ in the SCs than in the NCs (Figure 4D; *n* = 29 cells). Here also, all the individual cells that were of length ≤ 2.6 μm were taken as the SCs, while the cells of length > 2.6 μm were taken as the NCs, as reported by us earlier (Vijay et al., 2017). Thus, the SCF cells contained significantly higher levels of labile Fe^2+^, as compared to the NCF cells, indicating that there is heterogeneity in the labile Fe^2+^ levels in the mycobacterial subpopulations.

Further, like the *Msm* SCF cells, the *Mtb* SCF cells also contained significantly higher levels of labile Fe^2+^ per μg protein in the lysate as compared to the *Mtb* NCF cells (Figure 4E; *n* = 3). Thus, in *Msm* and *Mtb*, the levels of Fe^2+^ and H_2_O_2_ were significantly higher in the respective SCF cells as compared to that in the NCF cells despite the enormous difference in their habitats and physiological status. Thus, with respect to the generation of high levels of oxidative status, both the species have similar metabolic design.

### Inhibition of O_2_^∙⁣–^ and H_2_O_2_ Synthesis Decreases Labile Fe^2+^ Levels

Differences only in the labile Fe^2+^ levels, but not in the total iron content, between the SCF and the NCF cells implied the possibility of differential Fe^2+^ ion leaching in them. It is known that H_2_O_2_ and univalent oxidant radicals such as O_2_^∙⁣–^ cause Fe^2+^ leaching from iron-sulfur (4Fe-4S) cluster proteins (Flint et al., 1993; Keyer and Imlay, 1996; Imlay, 2006). Therefore, it was likely that the higher O_2_^∙⁣–^ and/or H_2_O_2_ levels in the SCF cells might have caused significantly higher leaching of Fe^2+^ from 4Fe-4S cluster proteins resulting in the higher labile Fe^2+^ levels. If this be the case, then inhibition of the synthesis of O_2_^∙⁣–^ (by way of inhibiting NADH oxidase) and/or H_2_O_2_ (by scavenging away H_2_O_2_) should considerably reduce labile Fe^2+^ leaching and hence the levels of labile Fe^2+^ in the SCF and the NCF cells. This possibility was tested by determining the levels of labile Fe^2+^ in the SCF and the NCF cells, which were fractionated from the *Msm* cells cultured in the continuous presence of non-toxic concentrations of DMTU (H_2_O_2_ scavenger; 1 mM for 10^8^ cells/ml; Supplementary Figures S1A–C; Parker et al., 1985) or DPI (NADH oxidase inhibitor; 100 nM for 10^8^ cells/ml; Supplementary Figures S1D–F; Li and Trush, 1998; Yeware et al., 2017). The levels in the DMTU/DPI-exposed SCF and NCF cells were compared with the labile Fe^2+^ levels in the SCF and NCF cells fractionated from the respective unexposed cultures. The DMTU/DPI-exposed SCF and NCF cells showed significant reduction in the labile Fe^2+^ levels when compared to the SCF and NCF cells from the respective unexposed cultures (Figure 4F). This experiment confirmed that the significantly higher levels of labile Fe^2+^ in the SCF cells were due to the higher levels of H_2_O_2_ and O_2_^∙⁣–^ in them, probably causing Fe^2+^ leaching from 4Fe-4S proteins. The increased levels of intracellular free iron could undergo Fenton reaction with higher levels of H_2_O_2_ and elevate the production of hydroxyl radical.

### Confirmation of Labile Fe^2+^ Leaching From 4Fe-4S Proteins Caused by O_2_^∙⁣–^ and H_2_O_2_

The Fe^2+^ leaching from 4Fe-4S cluster proteins in the SCF and NCF cells was confirmed by comparing the activity of a 4Fe-4S marker protein, aconitase (Vasquez-Vivar et al., 2000; Gardner, 2002; Imlay, 2006). It has been well documented that the superoxide radical anion reacts with aconitase, resulting in the release of labile Fe^2+^ from aconitase, which can be used as an assay for Fe^2+^ leaching from aconitase due to superoxide anion (Vasquez-Vivar et al., 2000; Gardner, 2002). Hence, we assayed for aconitase activity in the SCF and NCF cells, which were fractionated from the *Msm* cells cultured in the absence and continuous presence of DMTU (H_2_O_2_ scavenger; 1 mM for 10^8^ cells/ml; Supplementary Figures S1A–C; Parker et al., 1985) or DPI (NADH oxidase inhibitor; 100 nM for 10^8^ cells/ml; Supplementary Figures S1D–F; Li and Trush, 1998; Yeware et al., 2017). The SCF cells, which were fractionated from the *Msm* cells unexposed to DMTU or DPI, showed significantly low aconitase activity as compared to that in the equivalent NCF cells, probably due to the high levels of Fe^2+^ ion leaching from the protein (Figure 4G). On the contrary, the SCF cells, which were fractionated from the *Msm* cells cultured in the continuous presence of DMTU or DPI, showed significant increase in the aconitase activity probably due to the prevention of Fe^2+^ ion leaching from the protein owing to low levels of superoxide and/or H_2_O_2_ (Figure 4G). This confirmed the involvement of H_2_O_2_ and superoxide in the labile Fe^2+^ leaching from aconitase, and probably from other 4Fe-4S proteins as well. The Fe^2+^ chelator, EDTA, significantly reduced aconitase activity (control sample). The ROS levels in the NCF cells also caused labile iron leaching as evident from the increased aconitase activity in the NCF cells fractionated from the DMTU/DPI-exposed *Msm* cells (Figure 4G). The significantly lower aconitase activity in the unexposed SCF cells, unlike in the unexposed NCF cells, implied higher extent of Fe^2+^ leaching in the SCF cells due to higher O_2_^∙⁣–^ and H_2_O_2_ generation. Thus, the inherently higher levels of H_2_O_2_ and Fe^2+^ in the SCF cells, unlike in the NCF cells (see Figures 2P, 4A), correlated well with the inherently higher levels of hydroxyl radical in the SCF cells (see Figure 2H). Thus, the ultimate effect of the significantly higher levels of O_2_^∙⁣–^, which led to the production of elevated levels of H_2_O_2_ and increased levels of labile Fe^2+^ leaching, was the generation of significantly higher levels of hydroxyl radical in the SCF cells as compared to that in the NCF cells. This natural and inherent difference in the metabolic status of the SCF and the NCF cells of mycobacteria raises the question as to what is the physiological benefit of this striking metabolic difference that might have occurred evolutionarily between the SCF and the NCF subpopulations of *Msm* and *Mtb* species.

### The Benefit of the Differential Levels of Hydroxyl Radical Between the SCF and the NCF Cells

#### The SCF and the NCF Cells Incur Oxidative Stress Induced Mutations

Hydroxyl radical being a mutagen without nucleotide sequence specificity (Imlay et al., 1988; Sakai et al., 2006), we examined the benefits of its differential levels between the SCF and the NCF cells in terms of resister generation frequency against two anti-tuberculosis antibiotics, rifampicin and moxifloxacin, which have completely different targets of action. For this purpose, the freshly fractionated *Msm* SCF and NCF cells were entirely plated on Middlebrook 7H10 agar containing rifampicin (125 μg/ml; 3× MBC; Swaminath, 2017) or moxifloxacin (0.5 μg/ml; 5× MBC; Swaminath, 2017). The colonies from the rifampicin and moxifloxacin plates showed mutations in the rifampicin resistance determining region (RRDR) and quinolone resistance determining region (QRDR) for rifampicin and moxifloxacin resistance, respectively (Supplementary Figures S3, S4, respectively; Takiff et al., 1994; Yue et al., 2003; Chien et al., 2016). These mutations were identical to and at identical positions reported for the rifampicin-resistant and moxifloxacin-resistant clinical isolates of *M. tuberculosis* (Takiff et al., 1994; Yue et al., 2003; Chien et al., 2016). Further, the nucleotide changes, C→T, G→T, and A→G observed, were reminiscent of oxidative stress induced mutations, as reported (reviewed in Cadet and Wagner, 2013), which correlated well with the high levels of oxidative stress in the cells. Since both the SCF and the NCF cells incurred oxidative stress induced mutations despite the SCF cells having significantly higher oxidative status than the NCF cells, we examined whether their resister generation frequency was strikingly different due to the significant difference in the hydroxyl radical levels in them.

#### The SCF Cells Possessed Significantly Higher Resister Generation Frequency Than the NCF Cells

**FIGURE 5:**
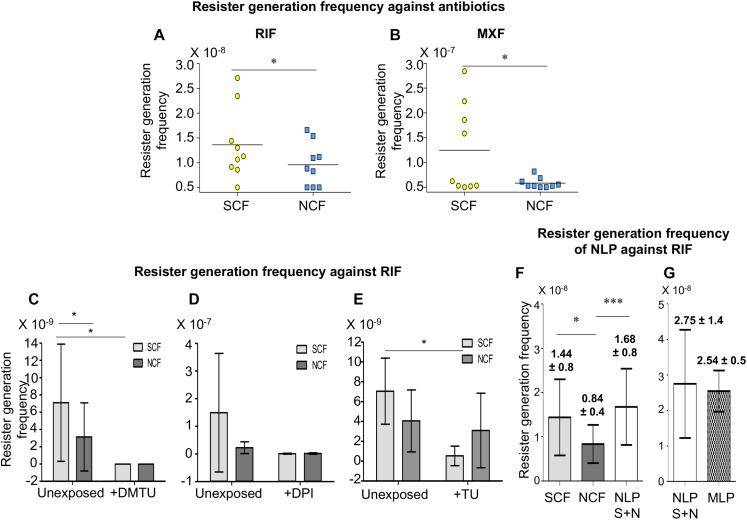
Resister generation frequencies of *Msm* SCF and NCF cells against antibiotics. **(A,B)** Resister generation frequencies of SCF and NCF cells when exposed to: **(A)** RIF and **(B)** MXF (*n* = 9 in each case). **(C–E)** Resister generation frequencies of SCF and NCF cells against RIF when exposed to: **(C)** DMTU (*n* = 7); **(D)** DPI (*n* = 3); **(E)** TU (*n* = 3). **(F)** Resister generation frequency of SCF, NCF, and NLP against RIF (*n* = 17). **(G)** Resister generation frequency of NLP and MLP against RIF (*n* = 3). S + N represents NLP constituted of unexposed SCF and NCF cells at 1:9 ratio. Statistical significance was calculated using paired *t*-test, where ^*^, ^∗∗∗^ indicate *p* ≤ 0.05, *p* ≤ 0.001, respectively.

Calculation of the resister generation frequency of the SCF and the NCF cells against rifampicin (RIF) and moxifloxacin (MXF) revealed that the resister generation frequency of the SCF cells was higher than that of the NCF cells by ∼two-fold against rifampicin and ∼five-fold against moxifloxacin (Figures 5A,B and Supplementary Figures S5A,B). The rifampicin resister generation frequencies of the SCF and the NCF cells, which were fractionated from the *Msm* cells cultured in the continuous presence of non-toxic concentrations of DMTU (H_2_O_2_ scavenger; 1 mM for 10^8^ cells/ml; Supplementary Figures S1A–C,I; Parker et al., 1985), or DPI (NADH oxidase inhibitor; 100 nM for 10^8^ cells/ml; Supplementary Figures S1D–F,I; Li and Trush, 1998; Yeware et al., 2017), or thiourea (TU; OH^∙⁣–^ scavenger; 0.5 mM for 10^8^ cells/ml; Supplementary Figures S1G,H,J; Tadolini and Cabrini, 1988), were found to be significantly reduced (Figures 5C–E, respectively and Supplementary Figures S5C–E, respectively). The survival of the cells in the presence of 1 mM DMTU or 100 nM of DPI or 0.5 mM TU, along with 125 μg/ml rifampicin, was found to be comparable to the survival of the cells in the presence of 125 μg/ml rifampicin alone (Supplementary Figures S1I,J). This indicated that the presence of the nontoxic concentrations of the respective inhibitors along with rifampicin did not cause any additional lethality, which could have otherwise been the cause for the decrease in the rifampicin resister frequency in the presence of DMTU/DPI/TU, along with rifampicin. Thus, the higher NADH oxidase expression in the SCF cells, resulting in the production of higher O_2_^∙⁣–^ levels, and consequentially higher levels of H_2_O_2_ and leached labile Fe^2+^ ions, led to elevated levels of hydroxyl radical generation, which in turn conferred the benefit of higher resister generation frequency on the SCF cells. On the contrary, the lower levels of NADH oxidase expression in the NCF cells consequentially resulted in the reduced resister generation frequency.

### The SCF:NCF Mixture Reconstituted at Their Natural Proportion Has Higher Resister Generation Frequency Than That of the SCF/NCF Cells

Further, we examined whether the significant differences in the individual resister generation frequencies of the SCF and the NCF cells would have any benefit on the whole population against the antibiotics when they were mixed together at the natural proportion at which they existed together in the MLP culture (Vijay et al., 2017). The mixture was made as per cfu/ml but mixed on the basis of v/v/v, which would give the expected cfu/ml (for details see section “CFU Determination and cfu-Based v/v/v Mixing of *Msm* SCF1, SCF2, and NCF to Obtain Different Proportionate Mixtures” in the Supplementary Material). This reconstituted mixture was termed Natural-Like Proportion (NLP). The NLP mixture was plated completely on multiple 3× MBC rifampicin plates. The SCF (SCF1 + SCF2) and the NCF cells, which were used to reconstitute the NLP mixture, were plated separately as individual samples also. The resister colonies from the SCF, NCF, and NLP samples showed mutations at the RRDR (Supplementary Figure S6). Again, these mutations were identical to and at identical positions reported for the rifampicin-resistant clinical isolates of *M. tuberculosis* (Yue et al., 2003). Further, the observed nucleotide changes, C→T and A→G, indicated oxidative stress induced mutations (reviewed in Cadet and Wagner, 2013).

As expected, the resister generation frequency of the SCF cells was most often significantly higher than that of the NCF cells (Figure 5F and Supplementary Figure S7A). However, it was of interest to note that the resister generation frequency of the NLP mixture was significantly higher than that of the NCF cells and most often comparable to or even higher than that of the SCF cells (Figure 5F and Supplementary Figure S7A). Further, the resister generation frequency of the NLP mixture was comparable to that of the MLP cells (Figure 5G and Supplementary Figure S7B). It may be noted that the resister generation frequencies of the NLP mixture and the MLP population were always higher than that of the NCF cells, and most often comparable to or higher than that of the SCF cells (Compare the values in the Supplementary Figure S7B with those in Supplementary Figure S7C). The comparability of the resister generation frequency of the NLP mixture with that of the MLP population validated the authenticity of the cfu-based reconstitution of the NLP mixture, where the SCF1, SCF2, and NCF cells were existing at proportions comparable to their natural proportion in the MLP population.

### Further Validation of the Accuracy of the Reconstitution of the NLP Mixture

The accuracy of the reconstitution of the NLP mixture was further validated in terms of percentage survival against rifampicin, by comparing the extent of survival of the NLP mixture with that of the MLP population and the TRP, which contained a proportionately equal mixture of the cells from all the Percoll fractions (64 to 80% at 2% increment). Proving the accuracy of NLP reconstitution, there was no significant difference between the extents of percentage survival of the NLP mixture, the MLP population and the TRP mixture against rifampicin (Supplementary Figure S8A). TRP being a total mixture, it was not possible to determine the resister generation frequency of the individual fractions, which were used to reconstitute TRP. Hence only the percentage survival of TRP, as the whole mixture, could be scored.

### The Physiological Significance and Robustness of the ∼1:9 Natural Ratio of the SCs:NCs in MLP

We had earlier shown that the SCs and the NCs exist at the natural ∼1:9 ratio in the MLP culture (Vijay et al., 2017). With the NLP and the TRP populations cells showing an extent of survival comparable to that of the MLP population against antibiotics, we wanted to find out the physiological significance of the existence of the SCs and the NCs at the ∼1:9 natural ratio in the MLP culture. For this purpose, we compared the extents of survival of the unnatural mixtures of the SCF and the NCF cells. The unnatural mixtures, which were called the Un-Natural Proportions (UNPs; UNP1, UNP2, and UNP3), were prepared by mixing back the freshly prepared SCF and the NCF cells at unnatural ratios that do not exist in the MLP culture (Supplementary Figure S8B). Unlike the NLP mixture, all the three UNP mixtures showed a gradual decrease in the survival against rifampicin, as compared to that of the NCF cells, with the proportionate increase in the SCF cells and a corresponding decrease in the proportion of the NCF cells (Supplementary Figures S8C–E). This was expected as we had earlier found that the SCF cells were more susceptible to antibiotics than the NCF cells (Vijay et al., 2017; Nair et al., 2019). Thus, the disturbance of the 1:9 natural ratio of the SCs:NCs cells in the MLP population affected the survivability of the population as a whole against rifampicin. This confirmed the physiological significance of the 1:9 natural ratio of the SCs:NCs in the MLP population. Further, the NLP mixture showed significantly higher survival than the NCF cells against a wide range of rifampicin concentrations and for different durations of exposure (Supplementary Figure S9). This revealed the robustness of the naturally evolved ratio of 1:9 in which the SCs and the NCs exist in the MLP cultures. It is pertinent here to recall the fact that the SCs and the NCs exist in the same 1:9 ratio in the sputum of pulmonary tuberculosis patients also (Vijay et al., 2014b), alluding to its clinical relevance, probably in the generation of drug-resistant strains.

### The Higher Number of Resisters in the NLP Mixture Arises From the NCF Cells

Since the NLP mixture, which contained the SCF and the NCF cells at the natural ratio of 1:9, showed higher resister generation frequency that was comparable to or more than that of the SCF cells, we wanted to find out whether the higher number of the resisters from the NLP mixture emerged from the SCF or the NCF component of the NLP mixture. For this purpose, we scored for hygromycin-resistant colonies from two different NLP mixtures reconstituted with SCF:NCF at 1:9 wherein the SCF and the NCF cells were differently tagged. For making the two differently tagged NLP mixtures, we first prepared SCF1, SCF2, and NCF cells from *Msm* wild type culture (WT) and from the culture of *Msm*/pAKMN2-*ugfp_*m*_^2+^*-*hyg*^*r*^ integrant cells, carrying the stable single copy of the genome-integrated pAKMN2-*ugfp_*m*_^2+^*-*hyg*^*r*^ plasmid, cultured in the absence of hygromycin (Roy et al., 2004, 2012; Sebastian, 2016). We mixed the SCF1-WT and the SCF2-WT cells with the NCF/pAKMN2-*ugfp_*m*_^2+^*-*hyg*^*r*^ cells to obtain NLP Mixture 1. The NLP Mixture 2 was reconstituted with the SCF1/pAKMN2-*ugfp_*m*_^2+^*-*hyg*^*r*^ and the SCF2/pAKMN2-*ugfp_*m*_^2+^*-*hyg*^*r*^ with the NCF-WT cells. These two NLP mixtures were entirely plated on multiple plates containing 3× MBC rifampicin. From all the master rifampicin plates of each of the NLP mixture, all the colonies were patch-plated into a fresh set of plates, one without hygromycin and the other with hygromycin.

**FIGURE 6:**
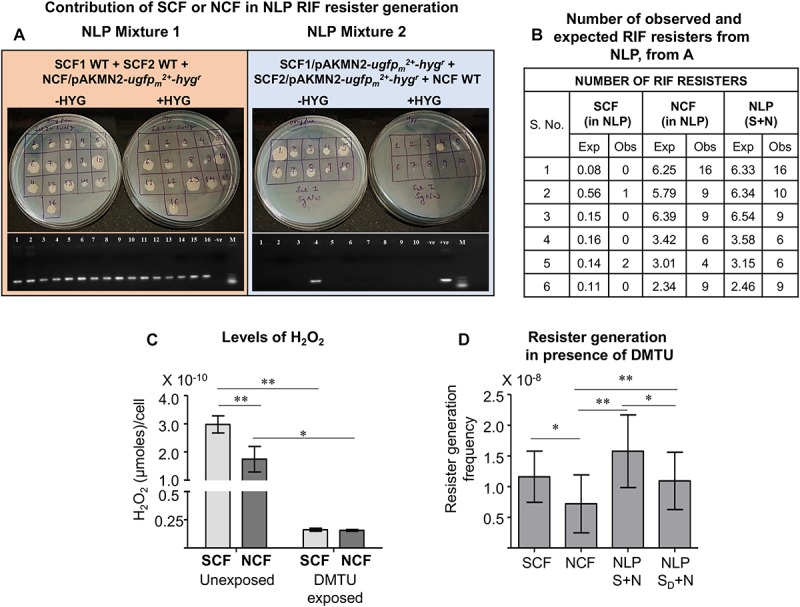
Contribution of the SCF and NCF cell to the RIF resister generation from NLP. **(A)** Upper panels: Colonies formed from the NLP cross-mixture 1 and 2 constituted with: (left panel) SCF1-WT, SCF2-WT, and NCF/pAKMN2-*ugfp_*m*_^2+^*-*hyg*^*r*^ integrant cells (NLP Cross-mixture 1); (right panel) SCF1/pAKMN2-*ugfp_*m*_^2+^*-*hyg*^*r*^, SCF2/pAKMN2-*ugfp_*m*_^2+^*-*hyg*^*r*^, and NCF-WT cells (NLP Cross-mixture 2). Lower panels in **(A)**: PCR amplification products of *ugfp_*m*_^2+^* from the genomic DNA of the RIF resisters from the NLP cross mixtures 1 and 2. **(B)** Table showing the number of observed and expected RIF resisters from the NLP cross-mixtures obtained from **(A)**. **(C)** Quantitation of H_2_O_2_ levels released by the SCF and NCF cells during unexposed and 1 mM DMTU-exposed conditions, measured using Amplex Red assay. Average of technical triplicates are represented in the graph. **(D)** Resister generation frequency of NCF in the presence of unexposed and DMTU-exposed (represented as subscript **D**) SCF cells. S_D_ + N indicates NLP mixture comprising of DMTU-exposed SCF cells and unexposed NCF cells at 1:9 ratio (*n* = 3). Statistical significance was calculated using paired *t*-test where ^*^, ^∗∗^ indicates *p* ≤ 0.05, *p* ≤ 0.01, respectively.

All the 16 rifampicin resister colonies obtained from the NLP Mixture 1 (SCF1-WT:SCF2-WT:NCF/pAKMN2-*ugfp_*m*_^2+^*-*hyg*^*r*^) grew on both the hygromycin-lacking and the hygromycin-containing plates (Figure 6A, the left panel showing plates). It indicated that all the 16 colonies had emerged from the NCF/pAKMN2-*ugfp_*m*_^2+^*-*hyg*^*r*^ cells only. On the contrary, out of the 10 colonies of the NLP Mixture 2 (SCF1/pAKMN2-*ugfp_*m*_^2+^*-*hyg*^*r*^:SCF2/pAKMN2-*ugfp_*m*_^2+^*-*hyg*^*r*^:NCF-WT), which grew on the hygromycin-lacking plate, only one colony grew on the hygromycin-containing plate (Figure 6A, the right panel showing plates). It indicated that out of the 10 colonies, which grew on hygromycin-lacking plate, nine colonies had emerged from the NCF-WT cells and one from the SCF (SCF1/pAKMN2-*ugfp_*m*_^2+^*-*hyg*^*r*^ or SCF2/pAKMN2-*ugfp_*m*_^2+^*-*hyg*^*r*^) cells. This observation was further validated by scoring for the presence of *ugfp_*m*_^2+^* in the genome of the colonies, which grew on hygromycin-lacking plate, using genomic DNA PCR (Figure 6A, the lower left and the right subpanels showing PCR bands). The number of rifampicin resisters, both expected (through calculation) and the observed (from the experiment), were quantitated from the SCF and the NCF cells in the NLP mixture and from the NLP mixture *per se*. It was observed that the major beneficiary of the higher resister generation frequency against rifampicin in the NLP mixture was the NCF cells, and the SCF1/SCF2 cells to a minor extent (Figure 6B).

### The SCF Cells Secrete H_2_O_2_ to Enhance Resister Generation From the NCF Cells in the NLP Mixture

The gain of higher resister generation by the NCF cells in the presence of the SCF cells in the NLP mixture showed that the SCF cells might have enhanced resister generation from the NCF cells, when they were together in the mixture. This was most likely possible through intercellular communication by means of some molecule. Among the different types of ROS (H_2_O_2_, O_2_^∙⁣–^, and OH^∙⁣–^), only H_2_O_2_ has high half-life (Dickinson and Chang, 2011) and can diffuse in and out of bacterial cells (Seaver and Imlay, 2001). Thus, the H_2_O_2_ secreted by the SCF cells might permeate into the NCF cells, undergo Fenton reaction with the available labile Fe^2+^ to generate OH^∙⁣–^, thereby inflict mutations and enhance resister generation. Supporting this possibility, the supernatant of the SCF cells was found to contain significantly high levels of H_2_O_2_ as compared to the supernatant of the NCF cells (Figure 6C). This amounted to 1.79 ± 0.182 × 10^8^ molecules of H_2_O_2_ secreted per SCF cell and 1.05 ± 0.27 × 10^8^ molecules of H_2_O_2_ secreted per NCF cell, which was ∼two-fold less than that secreted by the SCF cells. On the contrary, the supernatant of the *Msm* SCF and NCF cells, fractionated from the MLP culture grown in the continuous presence of non-toxic concentration of DMTU (H_2_O_2_ scavenger; 1 mM for 10^8^ cells/ml; Supplementary Figures S1A–C), showed significant reduction in the H_2_O_2_ levels (Figure 6C). The DMTU exposure reduced the amount of H_2_O_2_ secreted per SCF cell about twenty-fold, from 1.79 ± 0.182 × 10^8^ molecules to 9.72 ± 0.79 × 10^6^ molecules of H_2_O_2_. Similarly, in the case of the NCF cells also, the release of H_2_O_2_ was reduced about ten-fold, from 1.05 ± 0.27 × 10^8^ molecules to 9.38 ± 0.48 × 10^6^ molecules. Consequentially, the DMTU-exposed SCF cells, when mixed with the unexposed NCF cells at 1:9 ratio, significantly reduced rifampicin resister generation frequency of the NLP cross-mixture, as compared to the rifampicin resister generation frequency of the control NLP mixture containing the unexposed SCF and NCF cells at 1: 9 ratio (Figure 6D). It implied that the high H_2_O_2_ levels secreted by the SCF cells might have conferred the benefit of higher resister generation on NCF cells. Since the SCF cells constituted only a minor proportion of the NLP mixture (∼1/10th of the population), the number of resisters emerging from the SCF cells in the NLP mixture would be very low. The emergence of only one hygromycin-resistant colony out of 10 colonies (10%) of the NLP mixture 2 (SCF1/pAKMN2-*ugfp_*m*_^2+^*-*hyg*^*r*^:SCF2/pAKMN2-*ugfp_*m*_^2+^*-*hyg*^*r*^:NCF-WT) supports this fact.

### The Metabolic Design Behind the Inherently High ROS Generation in the SCs and Its Beneficial Effect on the NCs

The root cause for the high levels of ROS in the SCs is the high levels of expression of a specific NADH oxidase (MSMEG_6603) and the consequential higher levels of the active enzyme resulting in the production of significantly high levels of superoxide. This NADH oxidase is a flavoenzyme and flavoenzymes have been found to play role in the generation of hydrogen peroxide in *E. coli* (Korshunov and Imlay, 2010). While there was eight- nine-fold higher expression of the NADH oxidase in the SCF cells, they showed only one-fold higher levels of expression of the three SOD genes. Nevertheless, this level of SOD activity might be adequate to enable the SCF cells to convert the required quantity of superoxide into H_2_O_2_, which might be enough to engage the available labile Fe^2+^ in Fenton reaction to generate hydroxyl radical for genome-wide mutagenesis for resister generation, when confronted with antibiotics. This limited conversion of superoxide into H_2_O_2_, in turn, would ensure that there would still be adequate levels of superoxide available to effect leaching of labile Fe^2+^ from 4Fe-4S proteins. The levels of H_2_O_2_ synthesized would also might be enough for secretion into the medium to promote resister generation from the NCF cells. It may be noted here that the number of molecules of intracellular H_2_O_2_ present in the *Msm* SCF cells were found to be almost double the number of H_2_O_2_ molecules in the NCF cells (Table 1). The higher levels of H_2_O_2_ correlated with the higher levels of superoxide in the SCF cells. Similarly, the number of H_2_O_2_ molecules secreted by the SCF cells is ∼two-fold higher than that secreted by the NCF cells. The higher levels of H_2_O_2_ secreted by the SCF cells might be enabling the enhancement of the resister generation from the NCF cells in the NLP mixture and MLP, as found in our study.

**TABLE 1:** The number of H_2_O_2_ and Fe^2+^ molecules in the *Msm* and *Mtb* SCF and NCF cells.

	***Msm***	***Msm***	***Mtb***	***Mtb***
	**Per SCF cell**	**Per NCF cell**	**SCF (in μmoles) (per ug protein)**	**NCF (in μmoles) (per ug protein)**

H_2_O_2_ (intracellular)	4.25 ± 0.27 × 10^6^	2.55 ± 0.048 × 10^6^	2.75 ± 0.44 × 10^–5^	1.52 ± 0.38 × 10^–5^
H_2_O_2_ (extracellular)	1.79 ± 0.18 × 10^8^	1.05 ± 0.27 × 10^8^	ND	ND
Labile Fe^2+^	1.11 ± 0.13 × 10^7^	0.36 ± 0.023 × 10^7^	1.69 ± 0.55 × 10^–5^	5.50 ± 1.06 × 10^–6^
Total iron (in Fe^2+^ form)	1.25 ± 0.22 × 10^7^	1.04 ± 0.17 × 10^7^	ND	ND

*The *Msm* and *Mtb* H_2_O_2_ values were calculated from the Figures 2P,Q, respectively. Similarly, the *Msm* and *Mtb* Fe^2+^ values were calculated from the Figures 4A,E, respectively. ND, not determined.*

When it comes to the levels of labile Fe^2+^ ions, the number of labile Fe^2+^ ions in the *Msm* SCF cells was found to be about threefold higher than the number of Fe^2+^ ions in the NCF cells, with the total amount of iron (measured in the Fe^2+^ form) remaining comparable (Table 1). This indicated significantly higher levels of Fe^2+^ leaching in the SCF cells as compared to the NCF cells. Further, the stoichiometry of the number of molecules of intracellular H_2_O_2_ and Fe^2+^ in the SCF cells worked out to be 1:2.6 (1.11 ± 0.13 × 10^7^ divided by 4.25 ± 0.27 × 10^6^). On the contrary, the stoichiometry of intracellular H_2_O_2_ and Fe^2+^ in the NCF cells worked out to be 1:1.4 (0.36 ± 0.023 × 10^7^ divided by 2.55 ± 0.048 × 10^6^). Thus, the SCF cells possess about 3-fold higher number of Fe^2+^ molecules than the H_2_O_2_ molecules, as compared to their ∼1:1 stoichiometry in the NCF cells (Table 1). In. *M. tuberculosis* also, while the intracellular H_2_O_2_ levels in the SCF cells were twofold higher than that in the NCF cells, the labile Fe^2+^ levels were threefold higher in the SCF cells. Thus, in terms of the levels of H_2_O_2_ and labile Fe^2+^ ions, the SCF cells were certainly geared up for the generation of elevated levels of hydroxyl radical.

**FIGURE 7:**
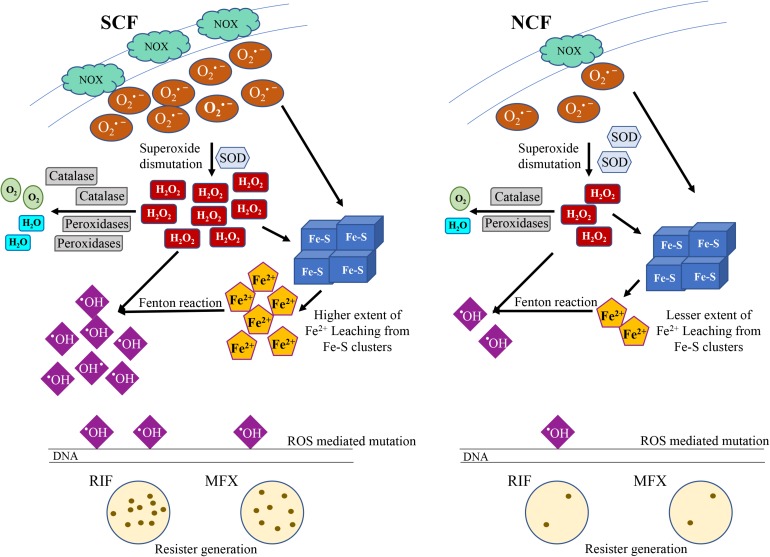
Functional model depicting the metabolic status of SCF and NCF cells that promote higher resister generation frequency and enhanced resister generation from the population. Higher expression of NADH oxidase resulting in high superoxide levels, which gets dismutated to generate H_2_O_2_. The superoxide and H_2_O_2_ levels result in the higher extent of Fe^2+^ leaching, which along with H_2_O_2_ undergo Fenton reaction to generate hydroxyl radical. Higher ROS levels eventually inflict higher rate of mutation and thereby increase resister generation from SCF cells. The NCF cells display lesser extent of such processes and thereby lesser resister generation.

The two-fold higher levels of *katG* expression in the SCF cells might ensure that the H_2_O_2_ levels, which are formed by the dismutation of superoxide by the SOD, do not reach deleterious levels. The two-fold expression levels of glutathione peroxidase (*gpx*) also might be contributing to reduce the harmful effects of H_2_O_2_ by converting it to water. The low levels of expression of *katG* and *gpx* in the SCF cells may be counterbalancing activities to maintain the oxidative stress under control to pre-empt harmful effects of the same. All these possible conclusions arising from the observations made in the present study indicate that the SCF cells might be maintaining a controlled atmosphere of higher level of oxidative stress, sufficient enough not only to generate mutations for survival on exposure to antibiotics, but also not to get subjected to the toxic effects of the oxidative stress at the same time. These metabolic designs in the SCF and the NCF cells are depicted in Figure 7.

Thus, the present study reveals that the SCF subpopulation inherently expresses NADH oxidase at significantly higher levels, which triggers the sequential cascade of generation of higher levels of O_2_^∙⁣–^, H_2_O_2_ and leached labile Fe^2+^, resulting in the generation of elevated levels of OH^∙⁣–^. This unique inherent feature of the SCF cells predisposed them to generate resisters at significantly higher frequency as compared to the NCF cells, despite their low proportion in the NLP/MLP/TRP. Although the NCF cells inherently have significantly lower resister generation frequency, the H_2_O_2_ secreted by the SCF cells benefited the whole population, in terms of higher frequency of resister emergence from the NCF cells in the MLP/NLP mixture.

## Discussion

### The Inherently High Oxidative Status of a Mycobacterial Subpopulation

We had earlier reported the presence of two subpopulations of the SCs and the NCs in the *in vitro* cultures of diverse species of mycobacteria, *M. smegmatis*, *M. tuberculosis* and *M. xenopi* (Vijay et al., 2014a,b, 2017) and of *M. tuberculosis* in the sputum of pulmonary tuberculosis patients (Vijay et al., 2014b). The present study reveals for the first time that these subpopulations individually have significantly different resister generation frequencies, and the subpopulation of SCs significantly enhances the resister generation frequency of the NCs, when they are together in the culture. However, it was demonstrated recently by us that the SCs inherently showed significantly higher susceptibility to oxidative stress induced by the exposure to anti-tuberculosis antibiotics than the NCs (Nair et al., 2019). Therefore, it was of interest to note that the SCs subpopulation confers the beneficiary act on the NCs despite its higher susceptibility to the oxidative stress generated against antibiotics. Thus, the trade-off for the SCs for the higher resister generation potential gained by the higher levels of ROS generation is the higher susceptibility. Nevertheless, since a whole population can be generated from a single cell, generation of even a single mutant against stress can restore the population in the face of even the most severe stress agents such as high bactericidal concentrations of antibiotics. In addition to this clear advantage to this subpopulation, the ability of this subpopulation to enhance the resister generation frequency of its kin subpopulation, the NCs, doubly ensures that the population does not perish even in the face of severest of stress conditions, probably even irrespective of the nature of the stress agent.

### The Act of the SCs Enhancing Resister Generation and Survival of the NCs May Not Be Altruistic

The behavior of the SCs, suffering from higher oxidative stress susceptibility, but enhancing the survival of its kin subpopulation, the NCs, in the SCs-NCs mixture against oxidative stress seemingly looks like an altruistic act. Here, the NCF cells, which form the major proportion of the population and the survival of which against oxidative stress was enhanced by the SCF cells (kin selection), form the beneficiary of the act of the SCF cells. Secondly, the benefit received by the NCF cells results in the protection of the major proportion of the population against oxidative stress. Thirdly, the SCF cells, which form only a subset of the population, sacrifice their viability during the act for the protection of their major kin subpopulation, the NCF cells. On account of all these features, the kin selection involved in the enhanced survival of the NCF cells by the SCF cells obeys Hamilton’s rule, which states that altruistic behavior is favored if *rb-c* > 0, where *r* is the genetic relatedness of the recipient to the donor, *b* is the benefit gained by the recipient, and *c* is the cost incurred by the donor (Hamilton, 1963, 1964). Here, *r* is the genetic relatedness of the NCF cells (recipient) to the SCF cells (donor) as evident from the regeneration of MLP population upon inoculation of SCF cells into fresh medium indicating full genome content as in the NCF cells, as earlier shown by us (Vijay et al., 2014a,b, 2017); *b* is the enhanced protection of the NCF cells (benefit gained by the recipient); and *c* is the higher susceptibility of the SCF cells (the cost incurred by the donor). For these reasons, the act of SCF cells conferring enhanced survival on the NCF cells conforms to the extension of kin selection theory, which states that the success of the altruistic act is high when there is genetic relatedness between the co-operators. However, despite all the conformity to an act of altruism, the act of SCF cells may not be a classical altruistic act since the SCF cells, despite higher susceptibility to stress and enhancing the survivability of its kin, the NCF cells, also gain the benefit of higher resister generation frequency and hence do not completely perish during the process under the stress condition. This is a gain on the SCF cells that may not categorize the phenomenon as a classical case of altruism.

### Expression of Specific Gene Systems Enabling Antibiotic Resister Generation in Bacteria

There are several examples of bacterial systems where expression of specific gene systems enables them to gain resistance to antibiotics. For instance, bacterial tolerance to fluoroquinolones was found to be enhanced by the elevated expression of the multidrug efflux pump, AcrAB-TolC, in response to the addition of an oxidative stress generator (Wu et al., 2012). Besides the induction of oxidative stress regulators and multidrug efflux pumps, ROS has also been reported to increase the tolerance levels in *E. coli* cells by decreasing the membrane potential and metabolism of the bacterial cells (Wang et al., 2017). Since a tolerant organism has better chances of survival under stress, it will benefit with a greater opportunity to acquire mutations (Levin-Reisman et al., 2017). Hetero-resistant methicillin-resistant *Staphylococcus aureus* (MRSA) strains have been reported to be comprised of two subpopulations, a minor subpopulation which exhibited resistance against higher drug levels and a major subpopulation, resistant to lower doses of the drug (Rosato et al., 2014). Further, the study has shown that the cells from the major subpopulation gain resistance to higher drug concentrations by the TCA-cycle mediated ROS generation which ensued in the SOS response leading to enhanced mutagenesis. Similarly, exposure of bacteria to antibiotics has been reported to incur oxidative damage, which when coupled with intracellular overload of iron, results in increased mutagenesis (Méhi et al., 2014). Interestingly, differential expression of exogenously introduced AcrAB-TolC, the main multidrug efflux pump in *E. coli*, correlated with the occurrence of spontaneous mutations in a subset of cells (El Meouche and Dunlop, 2018). However, though mutagenesis is known to be associated with a fitness cost which usually depends on the environment of the cells as well as the magnitude of the stress imposed on them (Melnyk et al., 2015), the cells eventually benefit by the evolution of antibiotic resistance.

The subpopulation of SCF cells stands as an excellent example to this phenomenon. The SCF cells, which suffers from fitness cost due to elevated levels of oxidative stress, ultimately stands to gain higher resister generation advantage that may help in the evolution of antibiotic resistance. The inherent and endogenous generation of high levels of ROS by the SCF cells, unlike in many cases known to be induced by external stress agents, stands as an exciting and unique aspect of mycobacterial physiology that may play a decisive role in the generation of drug-resistant strains in tuberculosis patients.

The high levels of H_2_O_2_ and Fe^2+^ in the *Msm* and *Mtb* SCF cells, and the consequential production of elevated levels of hydroxyl radical, as compared to their respective NCF cells, indicated that the SCF cells and the NCF cells of mycobacterial species, irrespective of the pathogenic status, have evolved in similar way such that the SCF cells have significantly higher oxidative status than the NCF cells. However, *M. tuberculosis* lacks the orthologs of all the four NADH oxidases that we analyzed in *M. smegmatis*. This alludes to the possibility that *M. tuberculosis* might be using NADH oxidases that may be very different from those in *M. smegmatis*. These possibilities need further detailed investigation to identify the genes involved in the generation of higher levels of H_2_O_2_ and Fe^2+^ in the *Mtb* cells.

### The Advantage of Inherent Generation of ROS by Mycobacterial Subpopulation

Environmental stress conditions can inflict mutations randomly, even in the absence of antibiotics, as a coincidental process, as there is no selection pressure (Knöppel et al., 2017). Such mutations, even if they occur on antibiotic resistance genes would give no specific survival advantage to the bacteria under environmental stress in the absence of antibiotics. However, such mutations, if they occur on antibiotic resistance genes, can confer resistance to antibiotics whenever the bacteria confront antibiotics. Thus, generation of a pool of mutants against diverse environmental stress conditions enabling selection when confronted with antibiotics has been demonstrated (Knöppel et al., 2017). Secondly, exposure of *E. coli* cells to sub-lethal concentration of one antibiotic has been found to lead to the development of resistance to other antibiotics due to random genome-wide mutations (Knöppel et al., 2017). In comparison to these examples on the possibility of gaining resistance to antibiotics, the specific advantage of mycobacterial population lies in the inherent ability of one of its subpopulations (the SCF cells) to generate endogenously, without any external stimuli or agents, elevated levels of ROS that confer resistance against antibiotics. It will be of interest to find out as to what factor in the subpopulation of SCF cells inherently triggers high levels of expression of the specific NADH oxidase gene. It is known that ROS production in bacterial systems can cause spontaneous DNA replication errors in proliferating cells, enabling emergence of random genetic mutations (Woo et al., 2018). If endogenously generated mutations, which can be induced by oxidative stress generated under diverse environmental stress conditions, occur on antibiotic target genes, they have been found to get selected when confronted with antibiotics. Here, mycobacteria score the specific advantage of being independent of external factors for the generation of oxidative stress, as it is produced endogenously and inherently by a subpopulation giving advantage to the population in resister generation and survival.

### Gain of Antibiotic Resistance in the Population Through External Molecules

Bacterial populations are known to gain enhanced tolerance against various antimicrobial agents through different strategies. Recent reports have proposed a strategy by which the highly resistant subpopulation of *Burkholderia cenocepacia* confers high level of resistance to the lesser resistant subpopulation against the antimicrobial peptide polymyxin B through the increased synthesis of a polyamine, putrescine, and a secreted protein, YceI (El-Halfawy and Valvano, 2013). Binding of polymyxin B to the surface of the bacterial cell was competed out by putrescine while YceI aided in sequestering polymyxin B and thereby protecting the cells from the toxic effect of the antimicrobial peptide (El-Halfawy and Valvano, 2013). Toxin used in the contact-dependent growth inhibition (CDI) has been reported to increase the persistence of bacterial cells through induction of ppGpp synthesis (Ghosh et al., 2018). This resulted in the Lon mediated degradation of the immunity protein thereby generating more of free toxin resulting in growth arrest. Yet another study has shown indole signaling in the bacterial population to enhance the antibiotic tolerance of a subpopulation via induction of the OxyR regulon (Vega et al., 2012). In addition to this mechanism, the bacterial cells were also found to develop persistence through the OxyR induction by H_2_O_2_ (Vega et al., 2012). In our study, the H_2_O_2_ secreted by the subpopulation of SCF cells as the possible agent that confers enhanced survival/resister generation frequency on the kin subpopulation of NCF cells emerges as another example of an external molecule playing a role in the enhanced protection of the population.

### Asymmetric Cell Division Contributing to Sister-Daughter Cells of Different Fates

Several biological systems generate sister-daughter cells which experience different fates post cell-division facilitating their development and propagation. Cell division in *Caulobacter crescentus* results in the generation of two daughter cells with different fates: the stalked cell and the swarmer cell for the propagation of their progeny (reviewed in Tsokos and Laub, 2012). Metabolic asymmetry in the daughter cells of alpha-proteobacteria, *Volvox carteri*, Drosophila, *Caenorhabditis elegans*, and in mouse has been reported to result in the different fates of their sister-daughter cells (Inaba and Yamashita, 2012). In addition to the development and propagation, the different fates of the sister-daughter cells also enable the population to circumvent various stress conditions. In this regard, irreversibly oxidized proteins (IOPs) were found to be partitioned asymmetrically between the sister-daughter cells in mycobacteria (Vaubourgeix et al., 2015). The progeny cells harboring less burden of IOPs exhibited better growth and survival against antibiotic stress in comparison to their siblings with higher IOP burden (Vaubourgeix et al., 2015). Further, a subpopulation of the fungal cells, *Cryptococcus gattii*, was found to attain tubular mitochondrial morphology when encountered with the host ROS (Voelz et al., 2014). This subpopulation was observed to promote the growth of the neighboring cells with non-tubular mitochondrial morphology and thereby help in the establishment of the pathogen in the host (Voelz et al., 2014). In the case of mycobacteria, using live cell imaging of mycobacterial cell division, we had earlier shown that the SCs are majorly generated through asymmetric cell division (Vijay et al., 2014a,b, 2017). Live cell imaging had also shown that symmetric division of the SCs also generated sister-daughter SCs (Vijay et al., 2017). Every asymmetric division produces one SC and one NC as sister-daughter cells (Vijay et al., 2017). Thus, asymmetric cell division forms the feeder line for the SCs. With the asymmetric cell division, the sister-daughter cells, the SCs and the NCs, become metabolically different in terms of their oxidative status. Since one of the major causes for the generation of oxidative stress is the differential expression of a specific *nadh* oxidase, it will be interesting to determine temporally when the differential expression of the gene occurs during asymmetric cell division. Such differential expression of gene between the SCs and the NCs arising from asymmetric cell division may not be confined to *nadh* oxidase alone, as revealed by the qPCR data. This can generate drastic metabolic differences between the SCs and the NCs.

### Clinical Relevance of the Findings

The present work clearly documents the existence of a natural minor subpopulation, in both the saprophytic and the pathogenic mycobacterial species, which has the inherent ability to generate antibiotic resisters at high frequency and significantly promote resister generation from its major kin subpopulation. Thus, the natural existence of this minor subpopulation and its inherent ability to generate antibiotic resisters from itself and to promote resister generation from its major kin subpopulation at high frequency may be one of the in-built causes for the emergence of antibiotic resistance in mycobacteria. The presence of such a subpopulation in the *M. tuberculosis* in tuberculosis patients will be an alarming scenario that could favor the emergence of drug resistance. The resister generation potential of this subpopulation was sourced to the high levels of expression and activity of MSMEG_6603, an NADH oxidase. However, *M. tuberculosis* does not possess the ortholog of *MSMEG_6603*, but has the ortholog of *MSMEG_1645* (another NADH oxidase with lower levels of expression in *Msm*). But the presence of significantly higher levels of H_2_O_2_ and Fe^2+^ in the *M. tuberculosis* H_37_R_v_ (virulent) SCF cells, as compared to the NCF cells (see Figures 2Q, 4E), clearly showed that *M. tuberculosis* H_37_R_v_ (virulent) strain also has the same inherent characteristic as *M. smegmatis* in generating differentially higher levels of ROS between its SCs and NCs. Thus, in *M. tuberculosis*, either the higher expression of the ortholog of *MSMEG_1645* or any of the oxidases in electron transfer that gets expressed at differentially higher levels in the *Mtb* SCs, might be involved in the ROS production. This needs to be verified on the SCs and the NCs of the *M. tuberculosis* H_37_R_v_ (virulent) strain and of the clinical samples of *M. tuberculosis*. Nevertheless, pending this verification, the presence of the SCs and the NCs at the same 1:9 ratio in the sputum of pulmonary tuberculosis patients (Vijay et al., 2014b) adds strength to the possibility that the SCs in the *M. tuberculosis* population, at least in the extracellular milieu, in tuberculosis patients might also have significantly higher levels of H_2_O_2_ and Fe^2+^ levels than the respective NCs. From these standpoints, and pending investigation on the nature and the proportion of SCs/NCs inside infected macrophages, the present study alludes to the strong possibility of the generation of antibiotic resisters from the SCs and promotion of antibiotic resister generation from the NCs in the whole population at least in the extracellular bacilli in tuberculosis patients.

## Data Availability

The raw data supporting the conclusions of this manuscript will be made available by the authors, without undue reservation, to any qualified researcher.

## Author Contributions

PA, RN, and DS conceived and designed the experiments. RN and DS performed the experiments. PA, RN, and DS analyzed the data. PA contributed reagents, materials, and analysis tools. PA, RN, and DS wrote the manuscript. All authors have read and approved the final manuscript.

## Conflict of Interest Statement

The authors declare that the research was conducted in the absence of any commercial or financial relationships that could be construed as a potential conflict of interest.
