# Supplementary material for: A Minor Subpopulation of Mycobacteria Inherently Produces High Levels of Reactive Oxygen Species That Generate Antibiotic Resisters at High Frequency From Itself and Enhance Resister Generation From Its Major Kin Subpopulation
Source: Front Microbiol. 2019 Aug 13;10:1842. doi: 10.3389/fmicb.2019.01842 (PMC6700507; doi:10.3389/fmicb.2019.01842)
Supplement: Supplementary file 1 [file Data_Sheet_1.PDF]

Supplementary Materials

Supplementary Materials and Methods

Percoll density gradient centrifugation for the fractionation of SCs and NCs

The preparative scale Percoll density gradient centrifugation for the enrichment of **s**hort **c**ells (**SC**s) and **n**ormal/long-sized **c**ells (**NC**s) from *Msm* and *Mtb* cultures to prepare **s**hort **c**ell enriched **f**raction (**SCF**) and **n**ormal/long **c**ell enriched **f**raction (**NCF**) were performed by scaling up the method as described (Vijay et al., 2017). The preparative scale Percoll density gradient centrifugation of *Msm* MLP cells was performed by using discontinuous gradients from 64% to 80% of Percoll, with an increment of 2% in the gradient. Whereas the preparative scale Percoll density gradient centrifugation of *Mtb* MLP cells was performed by using discontinuous gradients from 60% to 76% of Percoll, with an increment of 2% in the gradient (Vijay et al., 2017). In brief, the Stock Isotonic Percoll (SIP) was prepared in 9:1 ratio of Percoll (Sigma) and 1.5 M NaCl (Sigma) respectively. When required, the specific concentration of different inhibitors, [dimethyl thiourea (DMTU), diphenyleneiodonium chloride (DPI), thiourea (TU)], was added to SIP. The different percentage density of Percoll (64, 66, 68, 70, 72, 74, 76, 78 and 80% for *Msm* cells and 60, 62, 64, 66, 68, 70, 72, 74 and 76% for *Mtb* cells) were prepared by diluting the SIP with 0.15 M NaCl and making a total of 9 percoll fractions with different density. Subsequently, the gradient was made by carefully layering one ml at a time from each of the 4 ml Percoll fraction into 50 ml Beckman Coulter polycarbonate capped bottles (Beckman Coulter, catalogue no. 357002), from the highest percentage to the lowest percentage Percoll fraction.

Msm/Mtb cells from one/two 200 ml MLP culture in 1 litre flasks were pelleted down at ~7000 × g (Kubota centrifuge, with GSA cups) for 15 min and washed twice with 20 ml of sterile 0.5% Tween 80. Four ml of 0.5% sterile Tween 80 was added to the cell pellet, resuspended completely and syringed 15 times using 2 ml syringe to remove clumps, if any, and layered immediately onto the top of the Percoll gradient. These samples were subjected to centrifugation in JS 13.1 rotor in Beckman Coulter Avanti J-E centrifuge at ~350 × g for 1 hr at 20°C. Following the centrifugation, the cells in the 64, 66 and 78% fractions of *Msm* sample and 60, 62, 64, and 66% fractions of *Mtb* sample were pipetted out individually into corex tubes, diluted each of the fractions with 5 volumes of sterile 1x PBS and pelleted down. The pellet thus obtained from the SCF1 (64% Percoll fraction), SCF2 (66% Percoll fraction) and NCF (78% Percoll fraction) samples were resuspended in 500 µl of 1x PBS. Similarly, while the 60% and 62% were combined to get *Mtb* SCF1, 64% and 66% were used as SCF2 and NCF, respectively, as described (Vijay et al., 2017). The cell pellet in SCF1 and SCF2 were either made up individually or together (as SCF) to a final volume of 1 ml with 1x PBS. The cell pellet obtained from NCF was initially resuspended in 500 µl of 1x PBS and was visually made to the same cell density as that of the SCF1 and SCF2 individually by taking ~30 µl of NCF cells (present in 500 µl of 1x PBS) and making up the volume to 1 ml using 1x PBS, as described (Nair et al., 2019). To obtain higher cell number of SCs, SCF1 and SCF2 were pooled together (SCF) and NCF cell density was equated to that of SCF by taking ~60 µl of NCF cells (present in 500 µl of 1x PBS) into a fresh Eppendorf tube and the volume was made up to 1 ml using 1x PBS. Finally, both SCF and NCF cells were pelleted down at 3900 × g, 200 µl of 1x PBS was added to the SCF sample and 1 ml of 1x PBS was added to the NCF sample. An aliquot of 200 µl from this diluted NCF sample would be equivalent to the cell number of the respective SCF samples (either individually or together). The cell densities of SCF1, SCF2, SCF (SCF1 and SCF2 combined), and of NCF samples were verified by plating and comparing the cfu values obtained from 20 independent samples by independent people to ensure consistency and reproducibility. Following every Percoll gradient fractionation of the cells, the

purity checking of the SCF and NCF samples along with their respective enrichments were verified. While the purity checking was verified using acid fast staining, enrichments were ensured by scoring for the proportion of SCs and NCs in the different Percoll fractions using Carl Zeiss AXIO Imager M1 microscope and AxioVision 4 software, as described (Vijay et al., 2017).

Flow cytometric determination of the redox status of unexposed SCF and NCF cells using Mrx1-roGFP2 biosensor

Redox biosensor *Mrx1-rogfp2* downstream of *hsp60* promoter was subcloned from pMV762-Mrx1-roGFP2 (Bhaskar et al., 2014; kind gift from Amit Singh, Indian Institute of Science) into pAKMN2 (*hyg*^{r}) (Roy et al., 2012). The recombinant pAKMN2/*hsp60-Mrx1-rogfp2* construct was electroporated into electrocompetent *Msm* cells to obtain single copy of pAKMN2/*hsp60-Mrx1-rogfp2* integrated in the genome at the mycobacteriophage L5 *att* site (Nair et al., 2019). The integrant was cultured to MLP multiple times serially in the absence of hygromycin and then re-cultured in the presence of hygromycin to ensure stability of the integrant. The stable integrant was cultured to MLP following which the SCF1, SCF2 and NCF cells were fractionated from 200 ml culture using Percoll density gradient centrifugation, as described (Vijay et al., 2017). After equating the cell density of NCF with SCF, the samples were diluted 5 times in Middlebrook 7H9 media (see under "**Percoll density gradient centrifugation for the fractionation of SCs and NCs**") and 200 µl from these samples were added into 25 ml Middlebrook 7H9 media to obtain a cell density of 10^4 cells/ml. From these *Mrx1-rogfp2* integrated SCF and NCF samples, 500 µl was taken for flow cytometry analyses. Data was acquired using Becton Dickinson FACSVerse flow cytometer with 405 nm (V500) and 488 nm (FITC) solid state laser and 528/45 nm and 527/32 emission filter respectively, at medium flow rate. A high ratio of V500:FITC fluorescence indicative of increase in V500 fluorescence and decrease in FITC fluorescence shows high oxidative status. For analysis purpose, at least 10000 cells were gated from each sample. The median fluorescence for V500 and FITC was set to 2-$\log_{10}$ fluorescence units for the wild-type *Msm* cells for each time point, which served as the autofluorescence control. The voltage settings for the photomultiplier tube (PMT) were: 208 (FSC), 333 (SSC). Instrument calibration was performed with FACSuite cytometer set up and tracking (CS&T, Becton Dickinson) beads. FACSuite software was used for flow cytometric data processing and analysis. The biosensor response from the *Msm*/pAKMN2-*hsp60-Mrx1-rogfp2*samples was calculated by dividing the median fluorescence obtained at 405 nm (V500) with that obtained at 488 nm (FITC) and was used for plotting the graph. First, the median fluorescence for both V-500 and FITC was obtained from the gated cells. Subsequently, the ratio of V500:FITC median fluorescence was calculated. In order to analyse the ratio of V500:FITC median fluorescence of different subpopulations from the gated cells, a scatter plot with four quadrants was generated. The median fluorescence of both V500 and FITC for the cells in each quadrant was obtained and for each of the quadrant, the ratio of V500:FITC median fluorescence was calculated. Statistical significance between the time points was calculated using paired two-tailed *t*-test of graph pad prism version 5.0 (Motulsky, 2007).

Determination of hydroxyl radical levels using HPF-stained cells

Msm SCF and NCF cells were fractionated from 200 ml MLP culture using Percoll density gradient centrifugation and added into 25 ml Middlebrook 7H9 medium kept in 100 ml flask, to obtain a cell density of ~10^4 cells/ml, as mentioned under '**Flow cytometric determination of the redox status of unexposed SCF and NCF cells using Mrx1-roGFP2 biosensor**'. An aliquot of 500 µl cells was taken from these flasks immediately after addition of the cells, stained with 5 µM HPF (Setsukinai et al., 2003; Mukherjee et al., 2009) and incubated for 30

min at 37ºC under shaking conditions in the dark. Unstained cells were used as autofluorescence control and were also processed similar to the test samples. Data were acquired from the samples using Becton Dickinson FACS Verse flow cytometer with a 488 nm solid state laser and a 527/32 nm emission filter (GFP) at medium flow rate. The samples were processed similarly in the presence of 5 µM thiourea at the concentration that was found to be non-lethal for 10^4 cells/ml (Nair et al., 2019). For the analysis purpose, at least 10000 cells were gated from each sample. The photomultiplier tube (PMT) voltage settings were: 208 (FSC), 333 (SSC). FACSuite cytometer set up and tracking (CS&T, Becton Dickinson) beads were used for instrument calibration. Flow cytometry data were processed and analysed using FACSuite software. Statistical significance between the time points was calculated using paired two-tailed *t*-test.

Determination of H_2O_2 levels in cell lysate using Amplex Red assay

Msm SCF and NCF cells were fractionated from MLP culture using preparative scale Percoll density gradient centrifugation. The SCF and NCF cells were made to equal density as mentioned under "**Percoll density gradient centrifugation for the fractionation of SCs and NCs**." The cells in SCF and NCF were finally resuspended in 200 µl of Middlebrook 7H9 medium and from this resuspension 20 µl was used for cfu determination. Subsequently, the samples were pelleted down, resuspended in 500 µl of 1x PBS with the cell density of 10^7 cells/ml and subjected to sonication (at 30% amplitude, 1 sec pulse on and 1 sec pulse off, for a total duration of 2 min). Following sonication, an aliquot was taken for cfu determination to check the lysis efficiency which was found to be 99.9% consistently. The lysed samples were then filtered through 3 kDa cut off spin-filter (Amicon centrifugal filters) at ~8800 × g for 15 min, 4ºC in order to remove high molecular weight proteins. Simultaneously, the standards for Amplex Red assay using H_2O_2 (Invitrogen; Zhou et al., 1997; Mohanty et al., 1997) were prepared from 0.1 to 10 µM. H_2O_2 (20 mM) provided in the Amplex Red Hydrogen Peroxide/Peroxidase assay kit (Invitrogen) was serially diluted to 200 µM and 20 µM in 1x Reaction Buffer. The concentration of H_2O_2 in the tube was freshly determined every time before making up the working solution of H_2O_2 before every experiment. The H_2O_2 concentrations used for standards ranged from 0.1, 0.5, 1, 2, 4, 6, 8, 10 µM and were prepared in a total volume of 1 ml reaction buffer or 1x PBS. Following the filtration of the lysed samples, 50 µl of the filtrate was added into three different wells of a black multi-well plate (SPL Life Sciences), which served as the technical triplicates of the samples. Here, the 50 µl samples contained 10^6 cells/ml. Equal volumes of the H_2O_2 standards (50 µl each) were also added to the black multi-well plate. Subsequently, 50 µl of Amplex Red-HRP working solution (final concentration: 100 µM Amplex Red and 0.2 U/ml HRP, provided by Invitrogen) was added as per manufacturer's protocol into both the standards and the samples, kept for 30 min in the dark at room temperature. After the incubation, readings were taken at 530/590 nm in microplate reader (TECAN infinite 200 pro) and analysed using i-control software. Statistical significance was calculated using paired two-tailed *t*-test. In the case of DMTU or DPI exposure, the protocol for determining H_2O_2 concentration was carried out in the same manner, except that the *Msm* cells were grown till MLP in the continuous presence of DMTU or DPI.

Similarly, *Mtb* SCF and NCF cells were fractionated from 200 ml MLP culture and the cells were lysed by bead beating as mentioned (Kaser et al., 2008). The cells were resuspended in 500 µl 100 mM sodium acetate buffer and 200 ul of 0.1 mm Zirconia/Silica beads (BioSpec Products) were added. Subsequently, this mixture was subjected to bead beating in a mini bead beater (BioSpec Products) for 30 sec. This process was repeated twice, followed by centrifugation at ~ 3900 × g. Supernatant was collected and the remaining protocol for Amplex Red assay was followed in the same way as performed for *Msm* SCF and NCF cells.

Determination of superoxide levels in *Msm* SCF and NCF cells using Dihydroethidium (DHE) assay

The final pellets of *Msm* SCF and NCF cells obtained from 200 ml *Msm* MLP cultures were resuspended in 1550 µl of 1x PBS each. In order to determine the cfu of the samples, a 50 µl aliquot was taken from the cell suspensions. Further the samples were divided into two parts: 500 µl of the sample was taken for H_2O_2 determination using Amplex Red assay (as mentioned under "**Determination of H_2O_2 levels in cell lysate using Amplex Red assay**") and the remaining 1000 µl of the sample was taken for superoxide level detection and quantitation using dihydroethidium (DHE, Sigma) fluorescence assay (Yeware et al., 2017; Peshavariya et al., 2007; Nazarewicz et al., 2013). The detection of superoxide levels in the samples was carried out in the following manner: initially, the cells in 1000 µl were pelleted down at ~3900 × g at RT for 10 min. The cell pellet was further resuspended in 360 µl of Middlebrook 7H9 medium containing 100 µM diethylenetriaminepentaacetic acid (DTPA, Sigma) (Yeware et al., 2017; Fisher et al., 2004) and 50 µM DHE. This sample was then split into two parts: into one part, 10 µM TEMPOL (Sigma), a superoxide dismutase mimic (Yeware et al., 2017), was added while the other part was kept as such. The samples were then incubated at 37°C for 90 min under shaking condition. Along with the samples, Middlebrook 7H9 medium containing 100 µM DTPA and 50 µM DHE as well as Middlebrook 7H9 medium containing 100 µM DTPA, 50 µM DHE and 10 µM TEMPOL were also incubated which served as blanks. Following the incubation, the samples were taken into a 96-well black plate (SPL Lifesciences) and readings were taken from the top at 480/530 nm (Peshavariya et al., 2007; Nazarewicz et al., 2013) in microplate reader (TECAN infinite 200 pro) and analysed using i-control software. Firstly, the blank values were subtracted from the sample readings. Subsequently, the values obtained in the TEMPOL exposed samples were subtracted from the unexposed samples to determine the relative 2-OH-ethidium levels. This protocol was followed in order to obtain the fluorescence intensity of 2-OH-ethidium specifically from superoxide radical and not from other non-specific ROS sources. Statistical significance was calculated using Students' *t*-test.

Determination of nontoxic concentrations of DMTU, DPI and TU

In order to determine the concentrations of dimethyl thiourea (DMTU, H_2O_2 scavenger; Parker et al., 1985), diphenyleneiodonium chloride (DPI, NADH oxidase inhibitor; Li and Trush, 1998; Yeware et al., 2017) and Thiourea (TU, hydroxyl radical scavenger; Tadolini and Cabrini, 1988) which were non-lethal to the *Msm* cells, a wide range of concentrations of these inhibitors was used. For selecting the non-lethal concentration of DMTU, *Msm* cells (1%) were initially inoculated into different flasks containing fresh Middlebrook 7H9 medium. DMTU was added in the concentrations ranging from 0.25 mM -160 mM to different flasks except one, which served as the control. The cfu of the samples at 0 hr was then determined by plating the cells on Mycobacteria 7H11 agar. All the samples were then incubated for 12 hrs at 37°C. Following the incubation, the cfu of the samples were again determined to find out the non-lethal concentration of DMTU. The concentration of DMTU which showed survival comparable to the unexposed cells was then selected. In the case of DPI and TU exposure, the entire protocol was followed in the same manner, except that the concentrations of DPI ranged from 62.5 nM to 10 µM and that of TU ranged from 0.1 mM to 80 mM.

Exposure of *Msm* cells to DMTU, DPI and thiourea

Msm cells were grown in Middlebrook 7H9 broth in the continuous presence of different concentrations of either dimethylthiourea (DMTU, Sigma), diphenyleneiodonium chloride (DPI, Sigma) or thiourea (TU, Sigma) at 37°C, with shaking at 170 rpm, till the culture reached MLP. The highest concentration of DMTU, DPI or TU which was not lethal for the cells was selected. Further, the cells were grown in the continuous presence of the respective nontoxic

concentration of DMTU, DPI or TU, till the culture reached MLP, and was subsequently exposed to 125 μg/ml rifampicin for 24 hrs. The concentration of DMTU, DPI and TU that was found to be non-lethal to the cells in the presence of 125 μg/ml rifampicin was further used for the plating experiments. Thus, the cells were grown in the continuous presence of 1 mM DMTU, 100 nM DPI or 0.5 mM TU till the cultures reached MLP, following which the SCF and NCF cells were fractionated as mentioned under "**Percoll density gradient centrifugation for the fractionation of SCs and NCs**" and were used as per required protocol of respective experiment.

Determination of cfu

The *Msm* SCF and NCF samples were serially diluted as per their cell density and subsequently plated on Middlebrook 7H10 or Mycobacteria 7H11 agar plates to obtain cfu in countable ranges. The plates were then incubated at 37°C in bacteriological incubator. This protocol was followed for all the experiments in order to obtain cfu of the samples to calculate the data per cell.

Determination of NADH oxidase activity in SCF and NCF cell lysates

NADH oxidase is a membrane-bound enzyme which converts molecular oxygen to superoxide in the presence of NADH. The NADH in turn gets converted to NAD^+ and H^+. In order to measure the activity of NADH oxidase enzyme, the rate of NADH utilisation was calculated by measuring the reduction in the absorbance of NADH over time. Since NADH is also consumed and produced by many other cellular components, to obtain specificity of the assay, a parallel reaction containing the inhibitor of NADH oxidase, diphenyleneiodonium (DPI), was set up. The difference in the absorbance of these two reactions will specifically show the NADH oxidase activity. The SCF and NCF cells were obtained from 400 ml *Msm* MLP culture using a single Percoll density gradient centrifugation. Four such Percoll density gradient centrifugations were performed simultaneously, following which the SCF and NCF cells obtained were pooled together and made to equal cell density as described under "**Percoll density gradient centrifugation for the fractionation of SCs and NCs**." The cell pellets obtained from SCF and NCF were finally resuspended in 200 μl of 50 mM Tris-HCl buffer (pH 8), and an aliquot of 20 μl was taken for determining cfu. The lysis of cells was carried out by sonication at 30% amplitude, 1 sec pulse on, 1 sec pulse off for 2 min and repeated twice. Following sonication, an aliquot was taken for cfu determination to check the lysis efficiency which was found to be 99.9% consistently. The samples were then pelleted down at ~6800 × g for 10 min at 4°C to remove debris. A fresh tube was taken to collect the supernatant, from which 20 μl aliquot was used for protein estimation with Bradford assay. The rest of the sample was divided into four equal parts, where 40 μl of the sample was transferred into each Eppendorf tube. Into all the samples, 25 μl of 1 mM dithiothreitol (final concentration 100 μM) and 25 μl of 7.5 mM FAD (final concentration 750 μM) were added. For the autofluorescence control, the volume was made up to 250 μl by adding 160 μl of 50 mM Tris-HCl buffer (pH 8). To obtain the autofluorescence value for the negative control, 6.25 μl of 400 μM DPI (final concentration 10 μM; Yeware et al., 2017) and 153.75 μl 50 mM Tris-HCl buffer (pH 8) were added to make up the final volume to 250 μl. To the test sample, 135 μl of 50 mM Tris-HCl buffer (pH 8) was added to make up the volume and, just before taking the reading, 25 μl of 5 mM NADH (final concentration 500 μM) (substrate) was added. To the last tube, which also served as a negative control for the experiment, 128.75 μl of 50 mM Tris-HCl buffer (pH 8) was added along with 6.25 μl of 400 μM DPI (NADH oxidase inhibitor) and, before taking the reading, 25 μl of 5 mM NADH (final concentration 500 μM) (substrate) was added. Subsequently, the samples were transferred to transparent multi-well plate (Nunc), mixed well and readings were taken at 340 nm. First, the autofluorescence values were subtracted from the

test samples and negative control. Subsequently, the values of the DPI containing samples were subtracted from the samples which did not contain DPI to obtain the NADH conversion mediated specifically by NADH oxidase. A decrease in the NADH fluorescence at 340 nm during an interval of 30 min was calculated to obtain the activity of NADH oxidase in the SCF and NCF cell lysates. The concentration of NADH was calculated using the 6.22 $mM^{-1}cm^{-1}$ as the millimolar extinction coefficient of β-NADH at 340 nm (Reusch and Burger, 1974). Similar protocol was followed with the 100 nM DPI-exposed cultures as well. Data was plotted as NADH oxidase activity per cell for SCF and NCF samples. Statistical significance was calculated using Students' *t*-test.

Determination of superoxide dismutase activity in SCF and NCF cell lysates

The superoxide dismutase activity assay was performed in the SCF and NCF cell lysates using SOD Assay kit (Sigma-Aldrich), as per manufacturer's instructions. This kit-based method utilises a highly water-soluble tetrazolium salt WST-1 (2-(4-Iodophenyl)-3-(4-nitrophenyl)-5-(2,4-disulfophenyl)-2H-tetrazolium, monosodium salt) which, upon reduction with superoxide anion, generates a water-soluble dye formazan. Xanthine oxidase directly converts O_2 to superoxide which will reduce WST-1 and produces the coloured compound formazan. However, the presence of superoxide dismutase (SOD) will inhibit this reaction by utilising the superoxide and converting it to H_2O_2 and O_2. This will reduce the formation of formazan and hence less colour will be developed. Therefore, the reduction in the formazan colour formation is directly correlated with the SOD activity and is represented as percentage inhibition.

The cell pellets obtained were finally resuspended in 650 µl of buffer solution, an aliquot of 20 µl was taken for determining cfu. The lysis of cells was carried out by sonication at 30% amplitude, 1 sec pulse on, 1 sec pulse off for 2 min and repeated twice. The lysed samples were centrifuged at ~6800 × g for 10 min at 4°C to remove debris and supernatant was collected in a fresh tube. From each of SCF and NCF samples 210 µl was taken into two wells of transparent 96 well plate and 10 µl of WST solution with 20 µl enzyme working solution was added. Blank1 was prepared by adding 190 µl of buffer solution, 20 µl of dd H_20, 10 µl of WST solution and 20 µl enzyme working solution. Blank2 was prepared with the SCF and NCF cell lysates where 20 µl of dilution buffer was added instead of enzyme working solution. Blank3 was prepared as Blank1 without enzyme working solution and 20 µl of dilution buffer was added. A reaction was set up to be used as a control, where 210 µl of SCF and NCF cell lysate was added to two different wells and 5 mM sodium azide (Roberts and Hirst, 1996) was added into both in order to inactivate the superoxide dismutase enzyme. All the samples were mixed thoroughly using pipette and kept for incubation at 37 °C for 20 min. Absorbance was read at 450 nm using Tecan plate reader. The activity was calculated as per the given formula: SOD activity (inhibition rate %) = {[(Ablank 1 - Ablank 3) – (Asample - Ablank 2)]/ (Ablank 1 - Ablank 3)} x 100. The activity was normalized per cell for SCF and NCF samples as well as for the samples containing the SOD inhibitor sodium azide. Statistical significance was calculated using Students' *t*-test.

Total RNA preparation from unexposed *Msm* SCF and NCF cells

SCF and NCF cells were fractionated from ~5000 ml of *Msm* MLP culture using preparative scale Percoll density gradient centrifugation (from 400 ml culture each time) and following which the cell pellet was stored at -70°C till further use. Hot phenol method (Wecker, 1959; Ausubel & Kingston, 1987) with slight modifications for mycobacterial cultures was used for total RNA isolation from SCs and NCs. *Msm* SCF1, SCF2 and NCF cell pellets obtained from individual Percoll density gradient were taken from -70°C and pooled with the remaining samples to obtain larger cell pellet. The cells from SCF1, SCF2 and NCF pellets were then lysed using micro pestle for ~30 min with intermittent snap-freezing in liquid nitrogen and

thawing in the Eppendorf tube. In parallel, 1.2 ml lysis buffer, containing the final concentration of 100 mM sodium acetate, pH 5.2, 10 mM EDTA, 5 mM VRC, 1% SDS, with the remaining volume made up with DEPC-treated water, was prepared. Following the 30 min cell lysis with pestle, 1 ml of the lysis buffer was added to the samples to achieve complete lysis of the cells. Following the addition of the lysis buffer, the samples were mixed gently by inversion and subsequently divided into two equal halves of 500 µl each, into fresh tubes. Equal volume (i.e., 500 µl) of hot phenol (60ºC) was added into each sample, and the tubes were kept back at 60°C heating block for 10 min with intermittent mixing at every 2-min interval. After 10 min of heating at 60ºC, the samples were kept on ice for 5 min, followed by centrifugation at ~6800 × g for 10 min at 4ºC. Following centrifugation, the aqueous phase from the samples was collected into a fresh tube and again equal volume of hot phenol was added into, mixed by inversion and centrifuged again. This step was again repeated, and the final aqueous phase was collected into fresh tubes. Further, to the final aqueous phase collected in fresh tubes, equal volume of ice-cold phenol:chloroform mixture (1:1) was added and mixed by inverting the tubes gently 3-4 times. Subsequently, this mixture was subjected to centrifugation at ~6800 × g for 10 min at 4ºC. The aqueous phase was collected from the sample, followed by addition of equal volume of ice-cold phenol:chloroform mixture (1:1). The contents of the sample were mixed 3 to 4 times gently by inversion of the tube and subjected for centrifugation at ~6800 × g for 10 min at 4ºC. The final aqueous phase was then collected into a fresh tube and sodium acetate (0.3 M final concentration, pH 5.2) was added into it and mixed till a homogenous mixture was obtained. To this mixture, 2.5 volume of ice-cold 95% ethanol (Merck) was added, mixed by inversion and then kept for precipitation of total RNA at -70ºC overnight.

Following precipitation, the RNA was pelleted by centrifugation at ~6800 × g for 20 min at 4ºC. After the centrifugation, the supernatant was discarded, and the pellet was resuspended in 200 µl of 80% ethanol (Merck). The samples were then subjected to centrifugation at ~6800 × g for 10 min at 4ºC. The supernatant was again discarded, and the 80% ethanol wash was repeated once more. The pellet thus obtained was air-dried for 15 min at room temperature and dissolved in 50 µl of DEPC-treated water. The RNA sample was quantitated using **NanoDrop™ 1000** Spectrophotometer (Thermo Fisher Scientific) and stored at -70ºC. Following quantitation, the total RNA sample was run on formaldehyde agarose gel to check the RNA integrity of the preparation. After confirming the integrity, the RNA samples were treated with RNase-free DNase I (Thermo Fisher Scientific) to remove the DNA contamination in the RNA samples, as per the manufacturer's instructions. Aliquots of the RNA samples (~10 µg of RNA) were treated with 10 units of RNase-free DNase I, mixed by tapping and incubated at 37 ºC for 40 min.

Subsequently, the tubes were transferred to ice, and 200 µl of DEPC-treated water was added to the DNase I treated samples. To these samples, equal volumes of phenol (100 mM sodium acetate saturated phenol, pH 5.2): chloroform mixture (1:1) was added and mixed well by inverting the tubes. The samples were then subjected to centrifugation at ~6800 × g for 10 min at 4°C, the aqueous phase was collected into fresh tube to which sodium acetate (0.3 M final concentration, pH 5.2) was added and mixed well by tapping gently. To this mixture, 2.5 volumes of ice-cold 95% ethanol (Merck) was added and mixed well by inversion. The RNA samples were then kept for precipitation at -70ºC overnight. Following precipitation, the RNA samples from SCF1, SCF2 and NCF cells were subjected to centrifugation at ~6800 × g for 20 min at 4°C, after which the supernatant was discarded. Subsequently, the pellet was resuspended in 200 µl of 80% ethanol solution and again centrifuged at ~6800 × g for 10 min at 4°C. After the centrifugation, the supernatant was discarded, and the 80% ethanol wash of the RNA pellet was repeated once again. The final RNA pellet was then air-dried at room temperature for 15 min and dissolved in 20 µl of DEPC-treated water. The SCF1, SCF2 and

NCF RNA samples were quantitated using NanoDrop™ 1000 Spectrophotometer (Thermo Fisher Scientific) and stored at -70ºC until further use.

Preparation of cDNA for real time PCR

For the preparation of cDNA from each gene, 100 ng of total RNA was used from each of the three populations, SCF1, SCF2 and NCF. The cDNA synthesis reaction was carried out with the following constituents: 10 pmoles of gene-specific reverse primer (Sigma), 50 µM dNTP mix (Thermo Fisher Scientific), 20 units of RiboLock RNase inhibitor (Thermo Fisher Scientific), 200 units of RevertAid-Premium Reverse Transcriptase (RNaseH minus, thermostable, Thermo Fisher Scientific), and 1x RT buffer. The final volume of the reaction was made up to 20 µl by adding double-distilled autoclaved water. The cDNA synthesis was carried out using the following conditions: denaturation at 65ºC for 5 min, followed by annealing and extension combinedly for 30 min at 56ºC and inactivation of enzyme at 85ºC for 10 min. The cDNA thus obtained was stored at -20ºC till real time PCR was performed. cDNA for 16S rRNA from the same sample was used as the normalisation control.

Real time PCR

Real Time PCR was performed using Real Time PCR EvaGreen Mastermix (G-Biosciences) and the experiments were analysed according to comparative ΔΔCt method (Wang et al., 2006) followed by normalization (according to Willems et al., 2008). The real time PCR reaction was performed by addition of 10 µl of EvaGreen qPCR Mastermix-ROX (G-Biosciences), 10 pmoles each of gene-specific forward and reverse primers (**Supplementary Table S4**), 2 µl of cDNA and the remaining volume was made up to 20 µl using double-distilled autoclaved water. Technical triplicates were set up for each of the respective genes and real time PCR was performed in biological duplicates. For normalisation of each real-time experiment, 16S rRNA was used in each plate. The real time PCR reaction conditions were set up in the following manner: initial denaturation at 95ºC for 5 min, amplification of 40 cycles with three stage amplification comprising of 95ºC for 10 sec, annealing for 20 sec at 55ºC and amplification at 72ºC for 20 sec. In order to check the specificity of the primers and formation of specific product, melt curve was also prepared. The real time PCR experiment was performed using the Comparative Ct (ΔΔCt) method with CFX96 Real Time system from BioRad. The fold-change in the expression levels of mRNAs from *Msm* SCF1 and SCF2 cells with respect to that from the NCF cells was calculated. Statistical significance was calculated using Students' *t*-test.

MSMEG_6603 promoter cloning and genome integration to drive reporter gene

The NADH oxidase gene (MSMSEG_6603) sequence was analysed and 275 bp region upstream of the gene was selected as the promoter sequence. This 275 bp sequence was amplified using High fidelity Phusion DNA polymerase (Thermo Fisher Scientific) from the *Msm* genome with Msm-6603-P1-f and Msm-6603-P1-r specific primers with XbaI and EcoRV restriction sites, respectively (**Supplementary Table S5**) under the following conditions: initial denaturation at 98°C for 3 min; for each cycle of 35 cycles denaturation at 98°C for 30 sec; annealing at 62°C for 20 sec; extension at 72°C for 15 sec; and final extension at 72°C for 10 min (Sebastian, 2016). The amplified sequence was double-digested, gel purified and cloned in pBS (KS) using XbaI and EcoRV sites for sequence verification. Sequencing was carried out by Chromous Biotech, Bangalore, India, using universal forward and reverse primers. The recombinant vector was amplified in *E. coli* JM109 cells, plasmid was prepared and double-digested with XbaI and EcoRV for subcloning in pAKMN2-$ugfp_m^{2+}$ vector 5' to the reporter gene $ugfp_m^{2+}$ (Roy et al., 2012; Sebastian, 2016). The subcloned vector was purified using plasmid purification kit (GeneJet plasmid miniprep kit) and electroporated into *Msm* cells at voltage 1.5 kV, capacitance 25 µF, resistance 200 Ω using Gene PulserXcell™

electroporation system, Bio-Rad laboratories, Bio-Rad, CA, USA. The clones were confirmed by PCR amplification with Taq DNA polymerase (Thermo Fisher Scientific) using mycgfp2-RT-f and mycgfp2-RT-r primers (**Supplementary Table 4**) under the following conditions: initial denaturation at 95°C for 3 min; for each cycle of 35 cycles denaturation at 95°C for 30 sec; annealing at 64°C for 30 sec; extension at 72°C for 15 sec; and final extension at 72°C for 7 min. The pAKMN2-P_{MSMEG_6603}-$ugfp_m^{2+}$ integrant was cultured to MLP multiple times serially in the absence of hygromycin and then re-cultured in the presence of hygromycin to ensure stability of the integrant. For the purpose of culturing, the positive recombinant clone was grown in the presence of 50 µg/ml hygromycin.

Imaging of *Msm*/*pAKMN2*-P_{MSMEG_6603}-$ugfp_m^{2+}$

Msm/*pAKMN2*-P_{MSMEG_6603}-$ugfp_m^{2+}$ cells were grown till MLP and 1 ml of the cells were centrifuged at ~3900 × g for 10 min at room temperature. The pellet obtained was then resuspended in 200 µl of fresh Middlebrook 7H9 medium. Simultaneously, 1.9% low melting point agarose was used to make the agarose pad as described (Vijay et al., 2017). The solidified agarose pad was then used to layer the cells, covered with cover glass and kept for 10 min incubation at 37°C to adhere the cell on the agarose pad. Following incubation, the *Msm*/*pAKMN2*-P_{MSMEG_6603}-$ugfp_m^{2+}$ cells were taken under Zeiss AxioVision Imager M1and observed using DIC as well as fluorescence channel (GFP). The fluorescence intensities of the SCs and NCs were quantitated using ImageJ software. The area of the cell was calculated using ImageJ software and the fluorescence intensities were normalised per unit area of the cell. Statistical significance was calculated using paired two-tailed *t*-test.

Determination of Fe^{2+} levels in *Msm* SCF and NCF cell lysate using FeRhoNox™-1

The SCF and NCF cells obtained from 400 ml *Msm* MLP cultures were made to same cell density as described under "**Percoll density gradient centrifugation for the fractionation of SCs and NCs**." The cell pellets of SCF and NCF were resuspended in 200 µl of 1x PBS and 20 µl was taken to determine cfu. The remaining samples were pelleted down at ~3900 × g for 10 min at room temperature following which each sample was resuspended in 600 µl of 100 mM sodium acetate buffer (pH 5.2), in order to maintain acidic environment (to stabilise the labile iron in the ferrous form). Here, the cell density of the 600 µl samples was in the range of 10^7 cells/ml. The cell lysis was carried out by sonication at 30% amplitude, 1 sec pulse on and 1 sec pulse off for 2 min. Following cell lysis, the lysate was divided into two equal halves to estimate the levels of labile Fe^{2+} and total Fe^{2+}. The labile Fe^{2+} levels were estimated by passing the lysate through 3 kDa cut off spin-filter (Amicon centrifugal filters) and centrifugation at ~8800 × g for 15 min at 4ºC, to remove proteins with bound iron (Xiao and Kisaalita, 1997). The other half was used to estimate total Fe^{2+} by heating the lysate for 90 min at 90°C (modified protocol from Morones-Ramirez et al., 2013) and then centrifugation at ~3900 × g for 10 min at 4ºC to remove debris. Finally, into the labile and total Fe^{2+} samples, FeRhoNox™-1 (10 µM final concentration, Goryo Chemicals; Hirayama et al., 2013; Tsugawa et al., 2015) (from 1 mM stock solution in DMSO) was added. In parallel, the ferrous ammonium sulphate standards, ranging from 0.25 to 16 µM, were prepared in a final volume of 200 µl using 100 mM sodium acetate (pH 5.2). FeRhoNox™-1 (10 µM final concentration) was added into the samples and the standards. Subsequently, the samples and the standards were kept for 1 hr incubation at 37°C. In parallel, another set of labile Fe^{2+} samples were exposed to 100 µM 2,2'-bipyridyl, a Fe^{2+} ion chelator (Farhana et al., 2008) in order to confirm the specificity of FeRhoNox™-1 reaction with Fe^{2+}. Finally, the samples and standards were transferred into a transparent multi-well plate (Nest). FeRhoNox™-1 on interaction with Fe^{2+} generates the strongly fluorescing Rhodamine B. The readings of the samples were then taken at 540/575 nm

in microplate reader (TECAN infinite 200 pro) and analysed using i-control software. Statistical significance was calculated using paired two-tailed *t*-test.

The *Msm* MLP cells which were grown in the presence of 1 mM DMTU or 100 nM DPI were also subjected to Percoll density gradient centrifugation. Subsequently, the SCF and NCF cells were assayed for their labile Fe^{2+} levels using FeRhoNox™-1 assay. Statistical significance was calculated using Students' *t*-test.

Similarly, the *Mtb* SCF and NCF cells were fractionated from MLP cultures and lysed by bead beating as mentioned under "**Determination of H_2O_2 levels in cell lysate using Amplex Red assay".** Estimation of labile Fe^{2+} levels in the SCF and NCF cells was carried out using FeRhoNox™-1 assay and the data was plotted as labile Fe^{2+} levels per cell for the SCF and NCF cells in the same way as performed for *Msm* cells. Statistical significance was calculated using Students' *t*-test.

Imaging of FeRhoNox™-1 stained *Msm* MLP cells

Msm MLP cells were stained with 10 µM FeRhoNox™-1 (final concentration) and kept for incubation at 37°C for 1 hr under shaking condition. Subsequently, the cells were centrifuged at ~3900 × g for 10 min at room temperature. The pellet obtained was then washed with fresh Middlebrook 7H9 medium, centrifuged again at 3900 × g, and resuspended in 200 µl of fresh medium. In parallel, 1.9% low melting point agarose was used to make the agarose pad, as described (Vijay et al., 2017). The stained cells were layered on the solidified agarose pad, covered with cover glass and incubated at 37°C for 10 min to adhere the cells onto the agarose pad. Following incubation, the stained *Msm* cells were taken under Zeiss AxioVision Imager M1 and observed using DIC as well as fluorescence channel (rhodamine). The fluorescence intensities of the SCs and NCs were quantitated using ImageJ software. The area of the cell was calculated using ImageJ software and the fluorescence intensities were normalised per unit area of the cell. Statistical significance was calculated using paired two-tailed *t*-test.

Determination of Aconitase activity in SCF and NCF cell lysates

Aconitase drives an enzymatic reaction where conversion of isocitrate to aconitate takes place, which is a part of citric acid cycle. The activity of the enzyme can be calculated by measuring the absorbance of aconitate at 240 nm. The increase in the absorbance represents the activity of the enzyme and decrease in the absorbance will show the inactivity/inefficiency of the enzyme aconitase. The cell lysate showing higher rate of aconitate production reflects the higher activity of aconitase enzyme. The concentration of aconitate was calculated using its millimolar extinction coefficient 3.6 mM^{-1} cm^{-1} (Kennedy et al., 1983). The SCF and NCF cells were obtained from 400 ml *Msm* MLP culture using a single Percoll density gradient centrifugation. Two such Percoll density gradient centrifugations were performed in parallel, following which the SCF and NCF cells obtained were pooled together and made to equal cell density as described under "**Percoll density gradient centrifugation for the fractionation of SCs and NCs**." The SCF and NCF cell pellets were finally resuspended in 360 µl of 50 mM Tris-HCl buffer (pH 8) containing a final concentration of 3 mg/ml lysozyme (chicken egg white, Fluka) and 1 mg/ml lipase (*Candida cylindracea*, Sigma). ***Sonication was not used for the cell lysis in order to avoid the inactivation of aconitase in the samples*** (Pechter et al., 2013). An aliquot of 20 µl was taken for determining cfu before lysozyme-lipase digestion. The samples were then incubated at 37°C for 1 hr and following the lysozyme-lipase digestion, an aliquot of 20 µl was taken for cfu determination to check lysis efficiency. Following the digestion, the samples were pelleted down at ~6800 × g for 10 min at 4°C and the lysate was transferred into a fresh tube. The volume of the samples was made upto 800 µl using 50 mM Tris-HCl buffer (pH 8) after the addition of the substrate, DL-isocitric acid trisodium salt hydrate (20 mM final concentration). The absorbance of the samples was taken at 240 nm every

1 min for 5 min and the concentration of aconitate was calculated using its millimolar extinction coefficient 3.6 mM^{-1} cm^{-1} (Kennedy et al., 1983). Subsequently, the aconitase activity of the samples was represented as units per cell (Pechter et al., 2013). Further, for the samples which served as the negative control, the entire protocol was followed in the same manner, except that 300 µM EDTA (Varghese et al., 2003) was added to the cell suspension (to inactivate aconitase) before the lysozyme-lipase digestion. Likewise, for the positive control samples, the cells were grown in the presence of 1 mM DMTU or 100 nM DPI after which the entire protocol was followed in the same manner. Statistical significance was calculated using paired two-tailed *t*-test.

Resister generation frequency determination of SCF and NCF against rifampicin and moxifloxacin

The final pellet of SCF and NCF cells (made to equal density as described under "**Percoll density gradient centrifugation for the fractionation of SCs and NCs**") from 400 ml *Msm* MLP culture following Percoll density gradient centrifugation was resuspended in 200 µl of Middlebrook 7H9 media and 20 µl was taken from the cell suspension to determine the cfu. The rest of the cells (10^8 cells/ml) were entirely plated on 125 µg/ml of rifampicin or 0.5 µg/ml of moxifloxacin. The number of resistant mutants on the antibiotic containing plate was divided by the total number of bacterial cells, determined from the antibiotic-free agar plates, to obtain the resister generation frequency for each sample against the respective antibiotic. The resister generation frequency of the samples was also calculated in the same manner for the cells grown in the presence of 1 mM DMTU, 100 nM DPI and 0.5 mM thiourea. Statistical significance was calculated using paired two-tailed *t*-test.

The differential antibiotic susceptibility of SCs and NCs in our earlier studies was observed at a cell density of 10^3 cells/ml (Vijay et al., 2017). However, in the present study, we tried to understand the inherent propensity of SCs and NCs to generate resisters. Therefore, the study was conducted at a higher cell density of 10^8 cells/ml where the presence of pre-existing resister is also highly probable. This cell density was chosen with an understanding that the cells would have been predisposed to the mutations due to the differential ROS generation during their growth in MLP.

Culture conditions for rifampicin or moxifloxacin resisters in liquid media

The resistant colonies were obtained from the 125 µg/ml rifampicin plates or 0.5 µg/ml moxifloxacin plates and inoculated individually into fresh Middlebrook 7H9 broth. These cells were grown till MLP at 37°C with shaking at 170 rpm.

CFU determination and cfu-based v/v/v mixing of *Msm* SCF1, SCF2 and NCF to obtain different proportionate mixtures

The *Msm* SCF1, SCF2 and NCF cells obtained from Percoll density gradient centrifugation were made to visually similar cell density as described under "**Percoll density gradient centrifugation for the fractionation of SCs and NCs**". Based on cfu analysis, a v/v/v ratio of 1:1:5 mixture of SCF1:SCF2:NCF cells was found to give their proportion similar to that exists in MLP, which was called **N**atural-**L**ike **P**roportion (**NLP**). For the preparation of NLP mixture (10^4 cells/ml), 20 µl each of SCF1 and SCF2 (out of the 500 µl) individually obtained from the preparative scale Percoll gradient centrifugation was initially diluted 50 times with Middlebrook 7H9 broth. In order to obtain 1:1:5 v/v/v mixing to make NLP, 100 µl of SCF1 and 100 µl of SCF2 (which were already diluted 50 times with Middlebrook 7H9 broth as mentioned above) were mixed with 500 µl of NCF (the density of which was visually made up to that of the undiluted SCF1 or SCF2, as both were of comparable density, as mentioned earlier).

For the preparation of **U**n-**n**atural **P**roportion 1, UNP1 mixture, where the SCF1, SCF2 and NCF were mixed back at the 1:1:1 v/v/v proportion at which they do not exist, the 50 times diluted SCF1 and SCF2 were initially prepared. To obtain 1:1:1 v/v/v mixing to make UNP1, 100 µl of SCF1 and 100 µl of SCF2 was mixed with 100 µl of NCF (the density of which was visually made up to that of the undiluted SCF1 or SCF2, as both were of comparable density, as mentioned earlier). For the preparation of UNP2 (1:1:2 v/v/v) and UNP3 (2:2:1 v/v/v) mixtures, 20 µl of NCF (the density of which was visually made up to that of undiluted SCF) was diluted 50 times with Middlebrook 7H9 broth. Subsequently, UNP2 mixture was prepared by mixing 100 µl of SCF1 and 100 µl of SCF2 (which were already diluted 50 times with Middlebrook 7H9 broth) with 200 µl of the 50 times diluted NCF. The UNP3 mixture was prepared by mixing 200 µl of SCF1 and 200 µl of SCF2 (which were already diluted 50 times with Middlebrook 7H9 broth) with 100 µl of the 50 times diluted NCF. The **T**otal **R**econstituted **P**opulation (TRP) was prepared by mixing equal volumes (100 µl each) of the cells from all the nine (64, 66, 68, 70, 72, 74, 76, 78, and 80%) Percoll fractions. To obtain a cell density of 10^3 cells/ml, 200 µl was taken from UNP2 and UNP3 each and added into 25 ml Middlebrook 7H9 broth kept in 100 ml flask. To obtain a cell density of 10^4 cells/ml, 100 µl was taken from UNP1, NLP and TRP each and added into 25 ml Middlebrook 7H9 broth kept in 100 ml flask. The cfu of each of the samples was determined by plating (n = 10; **SupplementaryTable 2**).

Equal cell density (10^3 or 10^4 cells/ml) of the respective *Msm* SCF1, SCF2, NCF, UNP2, UNP3 cells, and/or NLP, UNP1, TRP, and MLP cells were exposed to 25 µg/ml rifampicin for 4 hrs (Vijay et al., 2017) individually or combinedly (SCF1 and SCF2), as the case may be. The percentage survival of the different samples, in terms of cfu, against antibiotic was determined by plating the respective antibiotic-stressed cells and the unstressed cells on antibiotic-free plates.

Determination of the percentage survival of the cells against antibiotic

The percentage survival of the cells in the different samples against antibiotics stress was determined by plating the cells on antibiotic-free plates before the exposure to stress (i.e. at 0 hr) and after the exposure to stress (for specific duration) followed by incubating the plates at 37°C in bacteriological incubator. Each of the experiments was repeated using independent biological replicates. Statistical significance was calculated using paired two-tailed *t*-test.

Resister generation frequency determination of SCF, NCF and NLP against rifampicin

Following Percoll density gradient centrifugation, the final pellet of SCF and NCF cells from 400 ml *Msm* MLP culture was resuspended in 200 µl of Middlebrook 7H9 media and 20 µl was taken for cfu determination from the cell suspension. The remaining cells were entirely plated on 125 µg/ml of rifampicin. The resister generation frequency was calculated for each sample by dividing the number of resistant mutants on the antibiotic containing plate with the total number of bacterial cells, determined from the antibiotic-free agar plates. Similarly, the NLP cells were also prepared from the same SCF and NCF samples as mentioned under "**CFU determination and cfu-based v/v/v mixing of *Msm* SCF1, SCF2 and NCF to obtain different proportionate mixtures**". After taking an aliquot for determining cfu (20 µl), the remaining cells were entirely plated on 125 µg/ml (3x MBC) of rifampicin. The resister generation frequency of MLP cells was also determined in the similar manner. In the case of DMTU exposure, the resister generation frequency of the samples was also calculated in the same manner, except that the *Msm* cells were grown in the presence of 1 mM DMTU till MLP and further subjected to Percoll density gradient centrifugation. The NLP cells were then prepared using SCF cells obtained from DMTU exposed culture and NCF cells from unexposed culture which were mixed as mentioned under "**CFU determination and cfu-based v/v/v**

mixing of *Msm* SCF1, SCF2 and NCF to obtain different proportionate mixtures." Statistical significance was calculated using paired two-tailed *t*-test.

Calculation of expected rifampicin resisters from SCF and NCF individually in NLP mixture and from NLP cells *per se*

Since the proportion of SCF and NCF cells in the NLP mixture is known, using the individual resister generation frequency of the SCF and NCF cells, the expected resister generation frequency of NLP was calculated. For example: in Set 1 of the cross-mixture experiment, the NLP was constituted of SCF WT and NCF/pAKMN2-$ugfp_m^{2+}$-hyg^r. The cfu of the NLP mixture was determined and found to be 5.25 x 10^8 cells. Since the NLP mixture was constituted of known proportions of SCF and NCF, the contribution of these cells in the NLP mixture in terms of cfu was found out. Thus, the NLP mixture (5.25 x 10^8 cells) comprised of 9.37 x 10^6 cells of SCF and 5.16 x 10^8 cells of NCF. The resister generation frequency of the SCF and NCF cells was determined individually and was found to be 8 x 10^{-9} and 1.21 x 10^{-8}, respectively. Thus, the expected number of SCF resisters in the NLP would be 0.075 ([9.37 x 10^6 cells] x [8 x 10^{-9}]) and that of NCF resisters in the NLP would be 6.25 ([5.16 x 10^8 cells] x [1.21 x 10^{-8}]). Therefore, the expected number of resisters in NLP would be 0.075 + 6.25 = 6.325. The observed number of resisters from the samples was obtained from the experiment and was then compared with the expected number of resisters.

Genomic DNA isolation from *Msm* SCF, NCF and NLP resisters

Msm SCF, NCF and NLP resisters were cultured, as mentioned under Materials and methods "**Culture conditions for rifampicin or moxifloxacin resisters in liquid media**", in parallel with the *Msm* wild-type (WT) cells to prepare genomic DNA. Initially, cells were harvested from 20 ml culture by centrifugation at 5400 × g, for 10 min, at room temperature. The cell pellet was then resuspended in 1 ml of Tris-HCl-EDTA buffer (10 mM Tris-HCl and 1 mM EDTA, pH 8.0), containing lysozyme (chicken egg white, Fluka) and lipase (*Candida cylindracea*, Sigma) at a final concentration of 3 mg/ml and 1 mg/ml, respectively followed by incubation at 37°C for 3 hr. The cell lysis was further carried out by adding 2% SDS (final concentration) into the digested resuspension, incubated at 55°C for 15 min and centrifuged at 8000 × g for 10 min at 4°C. The supernatant was collected into a fresh tube and equal volume of 100 mM Tris-HCl (pH 7.8)-saturated phenol:chloroform was added. The samples were mixed well by gently inverting the tubes and centrifuged at 8000 × g, for 10 min at 4°C. After centrifugation, the aqueous phase was collected into a fresh Eppendorf tube and equal volume of 100 mM Tris-HCl (pH 7.8)-saturated phenol was added. The samples were mixed well by gently inverting the tubes and centrifuged at 8000 × g for 10 min at 4°C. This step was repeated once more, and the aqueous phase was collected into a fresh tube. To this aqueous phase, an equal volume of 100 mM Tris-HCl (pH 7.8)-saturated phenol:chloroform (1:1) was added and then centrifuged at 8000 × g, for 10 min at 4°C. Following the centrifugation, the aqueous layer was transferred into a fresh tube and an equal volume of chloroform was added. The sample was again centrifuged at 8000 × g, for 10 min at 4°C and the aqueous layer was transferred into a fresh tube. The precipitation of genomic DNA from the aqueous phase was carried out with 0.3 M sodium acetate (final concentration, pH 7.4), mixed well and subsequently 2.5 volumes of ice-cold ethanol (Merck) was added. For complete precipitation, the samples were stored at 4°C overnight. Following incubation, the precipitate was pelleted down at 8000 × g, for 10 min at 4°C, resuspended in 70% ethanol (Merck) and centrifuged at 8000 × g, for 10 min at 4°C. The genomic DNA pellet was air-dried at room temperature for 5 min and dissolved in 50 µl of Tris-HCl-EDTA buffer (10 mM Tris-HCl and 1 mM EDTA, pH 8.0). In order to remove RNA contamination from the final pellet, DNase-free RNase A (1 µl of 10 mg/ml stock solution, bovine pancreatic RNase DNase-free, Sigma) was added to the genomic DNA and

incubated for 30 min at 55°C. Following the incubation, the genomic DNA was re-extracted using the above-mentioned protocol. The final genomic DNA pellet was dissolved in 20 μl Tris-HCl-EDTA buffer (10 mM Tris-HCl and 1 mM EDTA, pH 8.0) and stored at 4°C.

Analysis of mutations in the *Msm* SCF, NCF and NLP resisters against rifampicin and moxifloxacin

The RRDR locus of the *rpoB* gene was amplified using the genomic DNA samples of rifampicin-resistant mutants from SCF, NCF and NLP. The amplification was carried out with RRDR-specific primers: Msm-rpoB-RRDR-f and Msm-rpoB-RRDR-r (**Supplementary Table 3**). Likewise, the QRDR locus of the *gyrA* gene was amplified using QRDR-specific primers: Msm-gyrA-QRDR-f and Msm-gyrA-QRDR-r (**Supplementary Table 3**) with the genomic DNA samples of MXF-resistant mutants as template from SCF and NCF. High fidelity Phusion DNA polymerase (Thermo Fisher Scientific) was used for the PCR amplifications carried out in the following manner: initial denaturation at 98°C for 3 min; for each cycle of 35 cycles denaturation at 98°C for 30 sec; annealing at 67°C for 20 sec (for RRDR primers) and at 64°C for 20 sec (for QRDR primers); extension at 72°C for 15 sec (for RRDR primers), 72°C for 20 sec (for QRDR primers); and final extension at 72°C for 10 min. GeneJet Gel Extraction Kit (Thermo Fischer Scientific) was used to purify PCR products of RRDR locus (413 bp) and QRDR locus (500 bp) followed by sequence verification. The sequencing reactions of the rifampicin and moxifloxacin resistant mutants were performed by Medauxin, Bangalore, or Chromous Biotech, Bangalore, India, using both forward and reverse primers specific for RRDR and QRDR, respectively. An authentic mutation was considered when a mutation appeared at the same nucleotide position in both the forward and reverse sequencing reactions of the mutants. The positions of the nucleotide change and the corresponding amino acid change were analysed.

Determination of H_2O_2 levels secreted from cells using Amplex Red assay

Preparative scale Percoll density gradient centrifugation was used to fractionate *Msm* SCF and NCF cells from MLP culture and the cells from both the samples were made to equal density as mentioned under "**Percoll density gradient centrifugation for the fractionation of SCs and NCs**". The cells in the SCF and NCF were finally resuspended in 200 μl of Middlebrook 7H9 medium and from this resuspension 20 μl was used for cfu determination. The remaining samples were pelleted down, resuspended in 1500 μl of 1x PBS and from this 50 μl of the cell suspension was added into three different wells of a black multi-well plate (SPL Life Sciences), which served as the technical triplicates of the samples.

In parallel, the standards for amplex red assay using H_2O_2 (Zhou et al., 1997; Mohanty et al., 1997) were prepared from 0.1 to 10 μM. Using the 1x reaction buffer (diluted from 5x reaction buffer, Invitrogen), H_2O_2 (20 mM) provided in the Amplex Red Hydrogen Peroxide/Peroxidase assay kit (Invitrogen) was serially diluted to 200 μM and 20 μM. The total volume of the H_2O_2 standards, which ranged from 0.1, 0.5, 1, 2, 4, 6, 8, 10 μM, was made up to 1 ml using 1x reaction buffer or 1x PBS. The H_2O_2 standards (50 μl each) were also added into the black multi-well plate. Subsequently to both the samples and H_2O_2 standards, 50 μl of amplex red-HRP working solution (final concentration: 100 μM amplex red and 0.2 U/ml HRP, provided by Invitrogen) was added as per manufacturer's protocol followed by incubation in the dark at room temperature for 30 min. The readings were then taken at 530/590 nm in microplate reader (TECAN infinite 200 pro) and analysed using i-control software. In the case of DMTU exposure, the entire protocol was performed similarly, except that the *Msm* cells were initially grown till MLP in the presence of 1 mM DMTU followed by their fractionation using Percoll density gradient centrifugation. Statistical significance was calculated using paired two-tailed *t*-test.

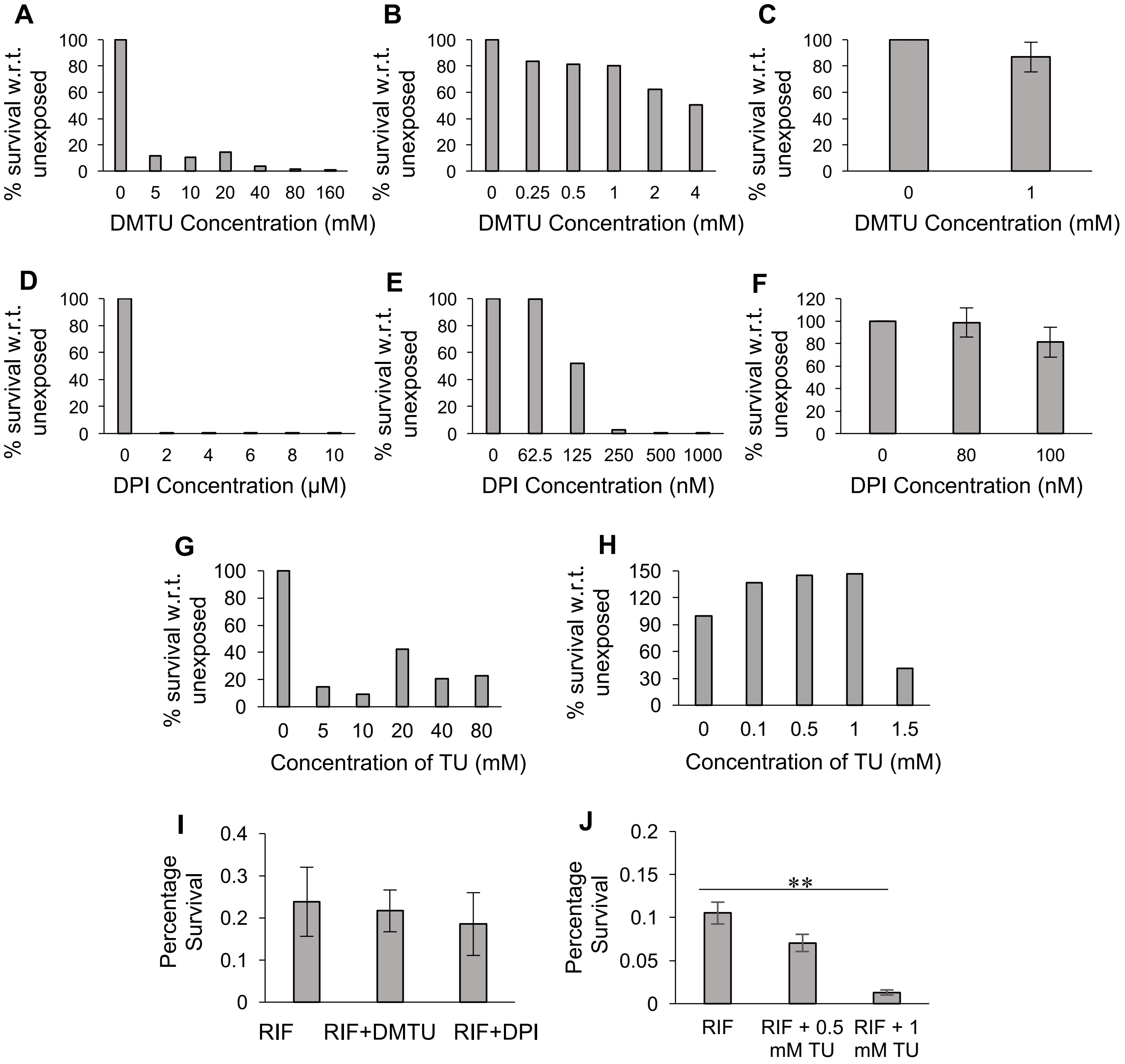

Supplementary Figure S1. Percentage survival of *Msm* MLP cells when exposed to different concentrations of dimethylthiourea (DMTU), diphenyleneiodonium (DPI), and thiourea (TU) for 12 hrs. Percentage survival of the exposed cells, with respect to the unexposed cells, upon exposure to: dimethylthiourea (DMTU), **(A)** 5 - 160 mM, **(B)** 0.25 - 4 mM and **(C)** 1 mM for 12 hrs. n = 1 for **(A)** & **(B)**, n = 3 for **(C)**; diphenyleneiodonium (DPI), **(D)** 2 - 10 µM, **(E)** 62.5 - 1000 nM, **(F)** 80 and 100 nM for 12 hrs, n = 3; thiourea, **(G)** 5 - 80 mM and **(H)** 0.1 - 1.5 mM for 12 hrs; **(I, J)** Percentage survival of *Msm* MLP cells when exposed to 125 µg/ml rifampicin (RIF) in the presence of **(I)** 1 mM DMTU and 100 nM DPI and **(J)** 0.5 mM and 1 mM thiourea for 24 hrs, n = 3. Statistical significance was calculated using paired *t*-test where ** indicates $p \leq 0.01$. **Presence of the nontoxic concentrations of the respective inhibitors along with RIF did not cause additional lethality**.

A

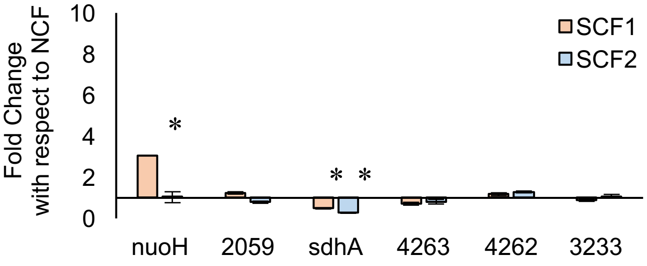

B

Respiratory and ETC components

nuoH	NADH-quinone oxidoreductase, H subunit
MSMEG_2059	NADH-quinone oxidoreductase chain e
sdhA	succinate dehydrogenase, flavoprotein subunit
MSMEG_4263	ubiquinol-cytochrome c reductase cytochrome b subunit
MSMEG_4262	ubiquinol-cytochrome c reductase iron-sulfur subunit
MSMEG_3233	cytochrome D ubiquinol oxidase subunit 1

C

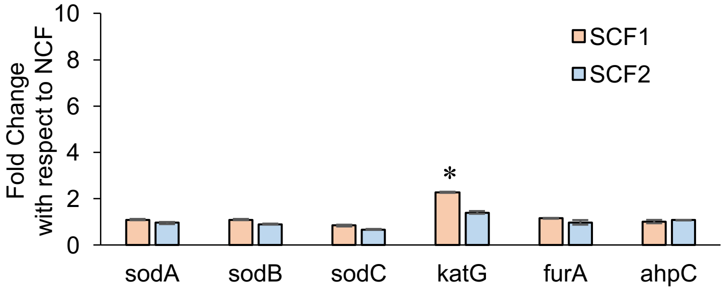

D

Antioxidants

sodA	[Mn] superoxide dismutase
sodB	[Mn] superoxide dismutase
sodC	copper/zinc superoxide dismutase
katG	catalase/peroxidase HPI
furA	transcription regulator FurA
ahpC	antioxidant, AhpC/TSA family protein

E

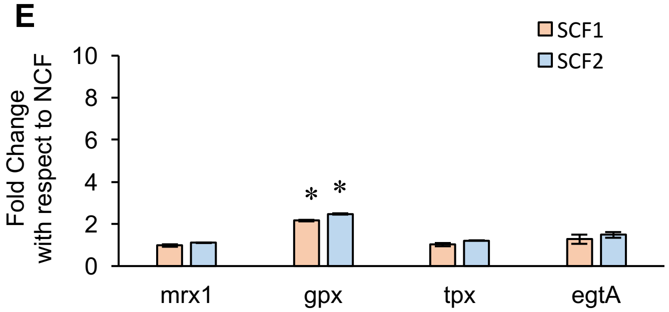

F

Antioxidants

MSMEG_1947	Glutaredoxin-like protein (mrx1)
MSMEG_3733	Glutathione peroxidase (gpx)
MSMEG_3479	thiol peroxidase, tpx
MSMEG_6250	Ergothioneine biosynthetic process, EgtA

Supplementary Figure S2. The qPCR data for the respiratory & ETC component genes and antioxidant genes of *Msm* cells. The qPCR data and the list of the genes analysed for: (A, B) respiratory & ETC component genes; (C, D) antioxidant genes; and (E, F) another set of antioxidant genes.

A

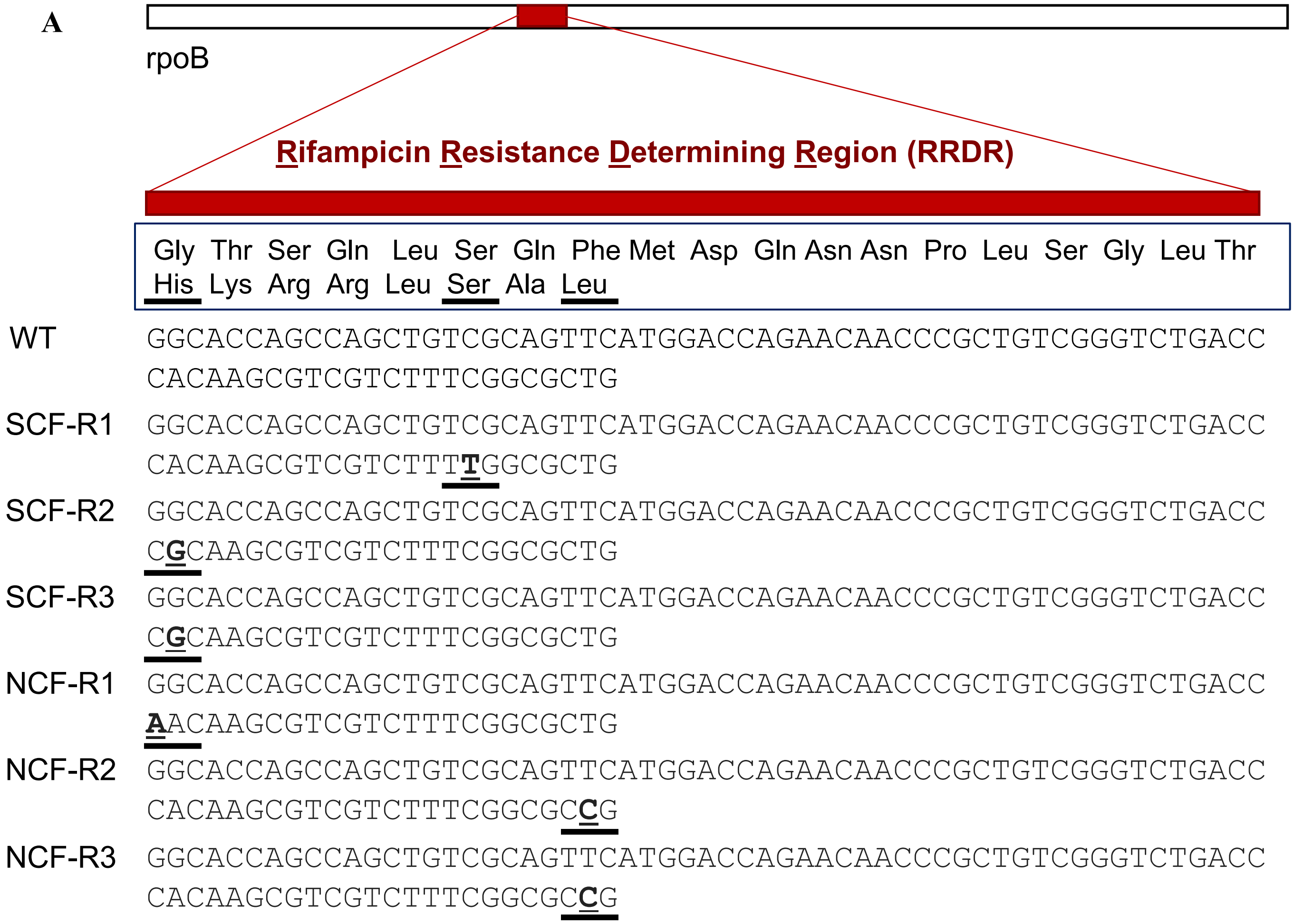

B

RRDR mutation analysis					
**Resister name**	**Mutation (codon)**	**Nucleotide (Position)**	**Amino acid change**	**Amino acid position**	**Type of mutation**
**SCF-R1**	**TCG-TTG**	**1340**	**S-L**	**447**	**C-T**
**SCF-R2 & R3**	**CAC-CGC**	**1325**	**H-R**	**442**	**A-G**
**NCF-R1**	**CAC-AAC**	**1324**	**H-N**	**442**	**C-A**
**NCF-R2 & R3**	**CTG-CCG**	**1346**	**L-P**	**449**	**T-C**

Supplementary Figure S3. RRDR mutation analysis in *Msm* SCF and NCF RIF resisters. (A) Representative images of *rpoB* gene harboring RRDR along with wild type amino acid as well as nucleotide sequence, and nucleotide sequence of SCF and NCF resisters against 125 µg/ml (3x MBC) RIF. **(B)** Table showing the positions of mutations found in the RRDR of SCF and NCF RIF resisters. Single letter underlined denotes the mutation in the sequence and triple letter underlined shows the mutated codon.

A

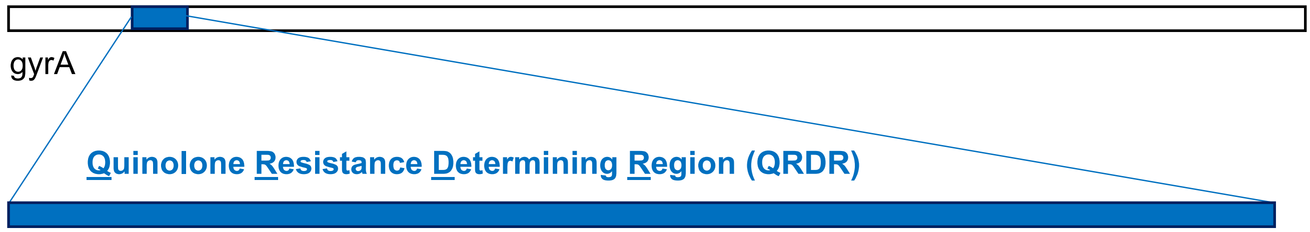

Ala Arg Ser Val Ala Glu Thr Met Gly Asn Tyr His Pro His Gly Asp Ala Ser Ile Tyr Asp
Thr Leu Val Arg Met Ala Gln Pro Trp Ser Leu Arg Tyr Pro Leu Val Asp Gly Gln

WT GCGCGCTCCGTTGCCGAGACGATGGGTAACTACCATCCGCACGGCGACGCCTCGATCTACGAC
ACCCTGGTCCGCATGGCCCAGCCGTGGTCGTTGCGCTACCCGCTGGTGGACGGCCAG

SCF-M1 GCGCGCTCCGTTGCCGAGACGATGGGTAACTACCATCCGCACGGCGACGCCTCGATCTAC**T**AC
ACCCTGGTCCGCATGGCCCAGCCGTGGTCGTTGCGCTACCCGCTGGTGGACGGCCAG

NCF-M1 GCGCGCTCCGTTGCCGAGACGATGGGTAACTACCATCCGCACGGCGACGCCTCGATCTACG**G**C
ACCCTGGTCCGCATGGCCCAGCCGTGGTCGTTGCGCTACCCGCTGGTGGACGGCCAG

B

QRDR mutation analysis					
**Resister name**	**Mutation (codon)**	**Nucleotide (Position)**	**Amino acid change**	**Amino acid position**	**Type of mutation**
**SCF-M1**	**GAC-TAC**	**283**	**D-Y**	**95**	**G-T**
**NCF-M1**	**GAC-GGC**	**284**	**D-G**	**95**	**A-G**

Supplementary Figure S4. QRDR mutation analysis in *Msm* SCF and NCF MXF resisters. (A) Representative images of *gyrA* gene harboring QRDR along with wild type amino acid as well as nucleotide sequence, and nucleotide sequence of SCF and NCF resisters against 0.5 µg/ml MXF. **(B)** Table showing the positions of mutations found in QRDR of SCF and NCF MXF resisters. Single letter underlined denotes the mutation in the sequence and triple letter underlined shows the mutated codon.

A

Resister generation frequency against RIF		
S. No.	SCF	NCF
1	9.41×10^{-9}	6.15×10^{-9}
2	1.84×10^{-8}	1.04×10^{-8}
3	0	5.95×10^{-9}
4	5.65×10^{-9}	3.83×10^{-9}
5	3.58×10^{-9}	0
6	4.12×10^{-9}	0
7	7.99×10^{-9}	3.26×10^{-9}
8	6.29×10^{-9}	0
9	2.21×10^{-8}	1.15×10^{-8}

B

Resister generation frequency against MXF		
S. No.	SCF	NCF
1	0	0
2	3.00×10^{-9}	2.85×10^{-9}
3	3.27×10^{-9}	3.04×10^{-9}
4	1.79×10^{-9}	0
5	1.22×10^{-8}	5.42×10^{-9}
6	1.08×10^{-7}	1.13×10^{-8}
7	1.73×10^{-7}	3.17×10^{-8}
8	2.34×10^{-7}	1.83×10^{-8}
9	1.36×10^{-7}	2.92×10^{-9}

C

Resister generation frequency against RIF + DMTU				
S. No.	SCF		NCF	
	UNEXPOSED	1 mM DMTU EXPOSED	UNEXPOSED	1 mM DMTU EXPOSED
1	0	0	3.29×10^{-9}	0
2	5.65×10^{-9}	0	3.83×10^{-9}	0
3	3.58×10^{-9}	0	0	0
4	4.12×10^{-9}	0	0	0
5	7.99×10^{-9}	0	3.26×10^{-9}	0
6	6.29×10^{-9}	0	0	0
7	2.21×10^{-8}	0	1.15×10^{-8}	0

D

Resister generation frequency against RIF + DPI				
S. No.	SCF		NCF	
	UNEXPOSED	100 nM DPI EXPOSED	UNEXPOSED	100 nM DPI EXPOSED
1	1.45×10^{-8}	0	1.36×10^{-8}	0
2	4.26×10^{-7}	3.13×10^{-9}	4.98×10^{-8}	5.05×10^{-9}
3	7.29×10^{-9}	0	4.73×10^{-9}	0

E

Resister generation frequency against RIF + TU				
S. No.	SCF		NCF	
	UNEXPOSED	0.5 mM TU EXPOSED	UNEXPOSED	0.5 mM TU EXPOSED
1	6.53×10^{-9}	2.13×10^{-9}	6.17×10^{-9}	3.83×10^{-9}
2	1.17×10^{-8}	0	0.74×10^{-8}	0
3	2.92×10^{-9}	0	2.64×10^{-9}	0

Supplementary Figure S5. Resister generation frequency of SCF and NCF cells. Resister generation frequencies of SCF and NCF cells when exposed to: **(A)** 125 μg/ml RIF, **(B)** 0.5 μg/ml MXF, **(C)** RIF and 1 mM DMTU, **(D)** RIF and 100 nM DPI, and **(E)** RIF and 0.5 mM TU. The data is from the SCF and NCF cells from: nine biological replicates for **(A & B),** seven biological replicates for **(C)** and biological triplicates for **(D)** and **(E).**

A

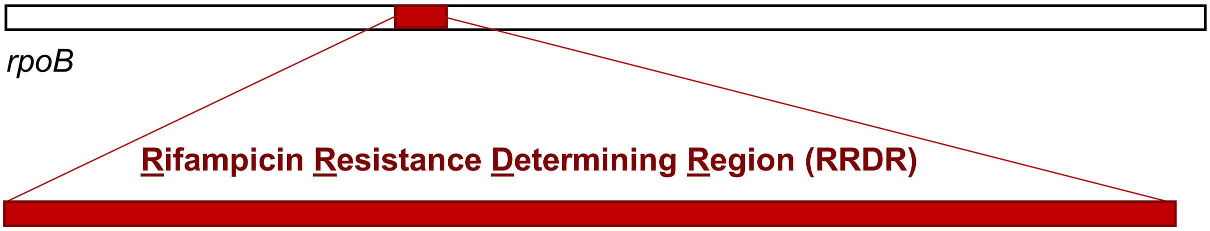

Gly Thr Ser Gln Leu Ser Gln Phe Met Asp Gln Asn Asn Pro Leu Ser Gly Leu Thr His Lys Arg Arg Leu Ser Ala Leu

WT GGCACCAGCCAGCTGTCGCAGTTCATGGACCAGAACAACCCGCTGTCGGGTCTGACC CACAAGCGTCGTCTTTCGGCGCTG

NLP1, NLP3 GGCACCAGCCAGCTGTCGCAGTTCATGGACCAGAACAACCCGCTGTCGGGTCTGACC CACAAGCGTCGTCTTT**T**GGCGCTG

NLP2 GGCACCAGCCAGCTGTCGCAGTTCATGGACCAGAACAACCCGCTGTCGGGTCTGACC C**G**CAAGCGTCGTCTTTCGGCGCTG

N1, N2, N3 GGCACCAGCCAGCTGTCGCAGTTCATGGACCAGAACAACCCGCTGTCGGGTCTGACC **T**ACAAGCGTCGTCTTTCGGCGCTG

S1, S2, S3 GGCACCAGCCAGCTGTCGCAGTTCATGGACCAGAACAACCCGCTGTCGGGTCTGACC **T**ACAAGCGTCGTCTTTCGGCGCTG

B

RRDR mutation analysis					
**Samples**	**Mutation (codon)**	**Nucleotide (Position)**	**Amino acid change**	**Amino acid position**	**Type of mutation**
**NLP-R1 & R3**	**TCG-TTG**	**1340**	**S-L**	**447**	**C-T**
**NLP-R2**	**CAC-CGC**	**1325**	**H-R**	**442**	**A-G**
**NCF-R1, R2 & R3**	**CAC-TAC**	**1324**	**H-Y**	**442**	**C-T**
**SCF-R1, R2 & R3**	**CAC-TAC**	**1324**	**H-Y**	**442**	**C-T**

Figure S6. RRDR mutation analysis in *Msm* SCF (S), NCF (N) and NLP RIF resisters. (A) Representative images of *rpoB* gene harboring RRDR segment along with wild type amino acid as well as nucleotide sequence, and nucleotide sequence of SCF, NCF and NLP resisters against 125 µg/ml RIF. **(B)** Table showing the positions of mutations found in RRDR sequence of SCF and NCF RIF resisters. Single letter underlined denotes the mutation in the sequence and triple letter underlined shows the mutated codon.

A

Resister generation frequency against RIF			
**S. No.**	**SCF**	**NCF**	**NLP S + N**
1	9.41×10^{-9}	6.15×10^{-9}	1.87×10^{-8}
2	8.89×10^{-9}	5.71×10^{-9}	1.60×10^{-8}
3	6.96×10^{-9}	8.18×10^{-9}	7.44×10^{-9}
4	2.29×10^{-8}	6.67×10^{-9}	1.38×10^{-8}
5	1.14×10^{-8}	1.20×10^{-8}	1.48×10^{-8}
6	7.27×10^{-9}	5.71×10^{-9}	1.28×10^{-8}
7	8.89×10^{-9}	8.00×10^{-9}	2.13×10^{-8}
8	1.71×10^{-8}	1.29×10^{-8}	2.23×10^{-8}
9	1.00×10^{-8}	1.63×10^{-8}	2.61×10^{-8}
10	8.00×10^{-9}	3.75×10^{-9}	9.33×10^{-9}
11	1.60×10^{-8}	7.69×10^{-9}	1.09×10^{-8}
12	1.60×10^{-8}	1.41×10^{-8}	5.71×10^{-9}
13	8.42×10^{-9}	6.49×10^{-9}	1.27×10^{-8}
14	2.50×10^{-8}	1.54×10^{-9}	1.68×10^{-8}
15	1.85×10^{-8}	1.04×10^{-8}	2.0×10^{-8}
16	4.00×10^{-8}	2.35×10^{-9}	1.33×10^{-8}
17	1.00×10^{-8}	1.43×10^{-8}	4.29×10^{-8}

B

Resister Generation Frequency against RIF					
**NLP (S+N)**			**MLP**		
**Total Cell No.**	**No. of resisters**	**Resister Generation frequency**	**Total Cell No.**	**No. of resisters**	**Resister Generation frequency**
2.00×10^{8}	9	**4.5×10^{-8}**	5.1×10^{9}	154	**3.01×10^{-8}**
5.50×10^{8}	11	**2.0×10^{-8}**	7.1×10^{9}	135	**1.90×10^{-8}**
2.88×10^{8}	5	**1.74×10^{-8}**	6.2×10^{9}	168	**2.71×10^{-8}**

C

Resister Generation Frequency against RIF					
**SCF**			**NCF**		
**Total Cell No.**	**No. of Resisters**	**Resister Generation frequency**	**Total Cell No.**	**No. of resisters**	**Resister Generation frequency**
1.75×10^{8}	4	**2.29×10^{-8}**	2.50×10^{8}	1	**0.4×10^{-8}**
5.50×10^{8}	6	**1.09×10^{-8}**	6.25×10^{8}	5	**0.8×10^{-8}**
1.88×10^{8}	3	**1.60×10^{-8}**	4.38×10^{8}	5	**1.14×10^{-8}**

Supplementary Figure S7. Resister generation frequency of *Msm* SCF, NCF and NLP cells against RIF. (A) Table showing the resister generation frequency of SCF, NCF and NLP cells against RIF (n = 17 independent samples in each case). **(B)** Comparison of the resister generation frequency of NLP and MLP cells against RIF. **(C)** Comparison of the resister generation frequency of SCF and NCF cells against RIF. These data pertains to **Figure 5 F, G**.

A

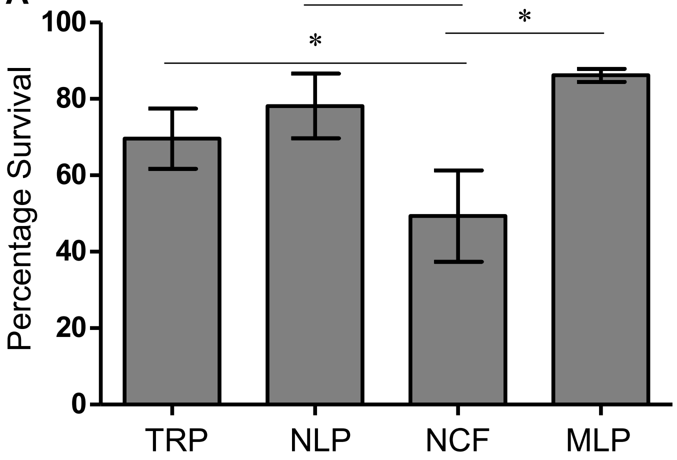

B

Unproportionate Mixtures	Average Proportion of Percoll fractions		
	SCF1 (%)	SCF2 (%)	NCF (%)
UNP1	6	9	85
UNP2	21	36	43
UNP3	29	59	12

C

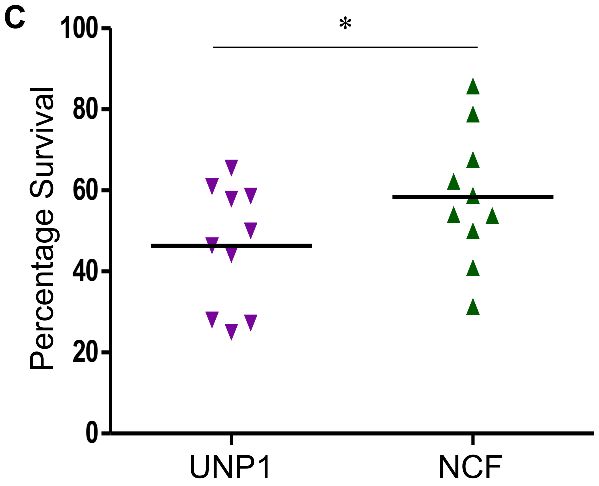

D

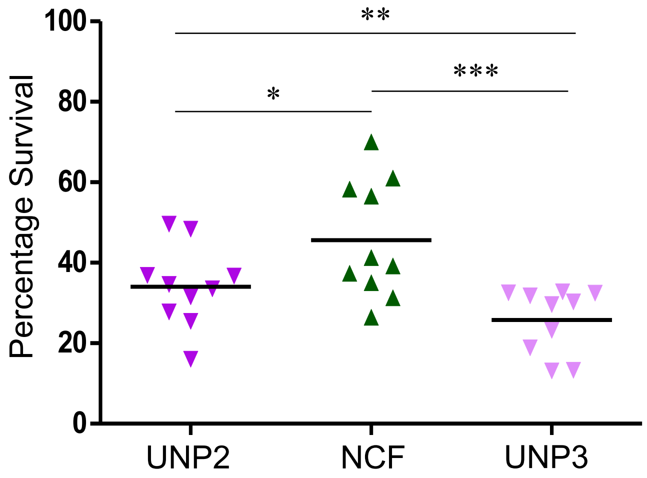

E

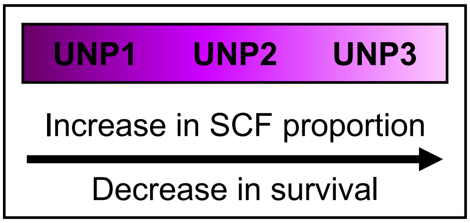

Supplementary Figure S8. Survival of NCF, TRP, NLP, MLP and un-natural proportionate (UNP) mixtures when exposed to 25 μg/ml RIF. (A) Exposure of NCF, TRP (Total Reconstituted Population), NLP (Natural-Like Proportion) and MLP (Mid-Log phase Population) to 25 μg/ml RIF. * indicates $p \leq 0.05$, statistical significance was calculated using paired *t*-test (n = 3). **(B)** Table representing the average proportion of SCF1, SCF2 and NCF in the different unnatural proportionate mixtures. **(C, D)** Percentage survival of UNPs in comparison to NCF on exposure to RIF for 4 hrs: **(C)** UNP1, **(D)** UNP2 and UNP3 (n = 10 independent samples in each case). **(E)** Correlation of the decrease in the percentage survival of the UNP mixtures with the increase in the SCF proportion.

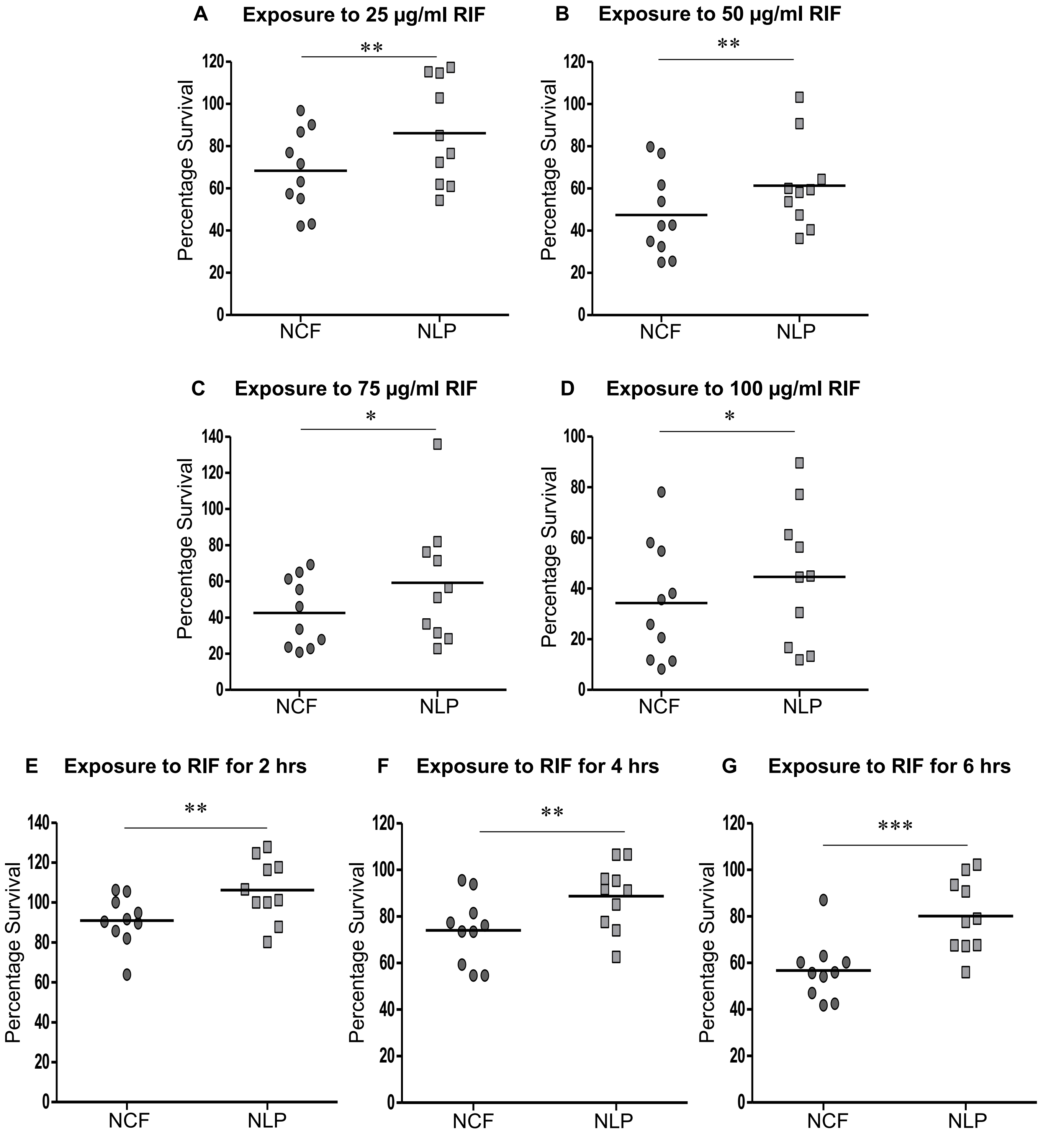

Supplementary Figure S9. Survival of NCF and NLP when exposed to different concentrations of RIF for different durations. (A-D) Exposure of NCF and NLP to RIF for 4 hrs at: **(A)** 25 µg/ml, **(B)** 50 µg/ml, **(C)** 75 µg/ml, and **(D)** 100 µg/ml. **(E-G)** Exposure of NCF and NLP to 25 µg/ml RIF for: **(E)** 2 hrs, **(F)** 4 hrs, and **(G)** 6 hrs. *, **, *** indicates p ≤ 0.05, p ≤ 0.01, p ≤ 0.001. Statistical significance was calculated using paired *t*-test (n = 10).

Supplementary Table 1. Bacterial strains and plasmids

**Bacterial strains or plasmid**	**Purpose**	**Reference**
**Bacterial strains**		
Mycobacterium smegmatis mc^2155	Experimental system	Snapper et al., 1990
Mycobacterium tuberculosis $H_{37}R_v$	Experimental system	Obtained from Central JALMA Institute for Leprosy and Other Mycobacterial Diseases, Agra, India
Escherichia coli JM109	Cloning host	Yanisch-Perron et al., 1985
**Plasmids**		
pAKMN2	Cloning vector	Roy et al., 2012
pMV762-Mrx1-roGFP2	Redox sensor	Bhaskar et al., 2014
pAKMN2-Mrx1-roGFP2	Redox sensor	Nair et al., 2019
pBS-KS	Sequencing vector	Alting-Mees & Short, 1989
pBS-KS-Mrx1-roGFP2	Sequencing vector	Nair et al., 2019
pBS-KS- P_{msmeg_6603}	Sequencing vector	This study
pAKMN2-$ugfp_m^{2+}$	Cloning vector	Sebastian, 2016
pAKMN2-P_{msmeg_6603}-$ugfp_m^{2+}$	Expression vector	This study

Supplementary Table 2. Mixtures of different proportions of *Msm* SCF1, SCF2, and NCF cells

Name of the Proportionate Mixture	SCF1:SCF2:NCF Average Proportion (± SD)	
	by cfu	by volume
NLP	2(±1) **:** 2(±1) **:** 96(±3)	1:1:5
UNP1	6(±4) **:** 9(±5) **:** 85(±9)	1:1:1
UNP2	21(±5) **:** 36(±9) **:** 43(±12)	1:1:2
UNP3	29(±6) **:** 59(±8) **:** 12(±5)	2:2:1
TRP	Equal volumes from all the percoll fractions	
MLP	**M**id-**l**og **p**hase culture (Unfractionated)	

NLP, **n**atural-**l**ike **p**roportion; UNP, **u**n-**n**atural **p**roportion; TRP, **t**otal **r**econstituted **p**opulation; MLP, **m**id-**l**og **p**hase population.

Supplementary Table 3. Oligonucleotide primers used for mutation analysis

**Name**	**Sequence**
Msm-RRDR-f	5'- GTCGTCTGCGCACCGTC -3'
Msm-RRDR-r	5'- CTCGATGAAGCCGAACGG -3'
Msm-QRDR-f	5'- ATGACTGATACGACGCTGCC -3'
Msm-QRDR-r	5'- AGAACCGTGGGCTCCTG -3'

Supplementary Table 4. Oligonucleotide primers used for Real Time PCR

Name	Sequence
Msm-nuoH-RT-f	5’-CGATCGGTGTCTACGGCATC-3’
Msm-nuoH-RT-r	5’-GACATCGTTCCCGCGTAGAG-3’
Msm-2059-RT-f	5’-TCTACGACAACCAGACACCG-3’
Msm-2059-RT-r	5’-GTGCGTTCGGATCGGTGAG-3’
Msm-sdhA-RT-f	5’-GTACCCGACGTGCCACTAC-3’
Msm-sdhA-RT-r	5’-TTGATGTCCAGCAGCGAGTT-3’
Msm-4263-RT-f	5’-ATCTTCTTCACCGGTGCGTT-3’
Msm-4263-RT-r	5’-CCATCGTGATACCGGAGAGC-3’
Msm-4262-RT-f	5’-TCGCAAAGCCCATATTCGGT-3’
Msm-4262-RT-r	5’-GATTTGCGCTCCCAGAATGC-3’
Msm-3233-RT-f	5’-CTCTGGACTTATCGCGGTGG-3’
Msm-3233-RT-r	5’-AAGAAACGAGTGAGGCGGTA-3’
Msm-6603-RT-f	5’-GTCGCGTACAAGCCCTATCC-3’
Msm-6603-RT-r	5’-GTCGTCGGTGTGGATGAAGT-3’
Msm-1645-RT-f	5’-TGGTCAACCCGATGTACCTG-3’
Msm-1645-RT-r	5’-CTCGCGGTAGGGGTATTCG-3’
Msm-2889-RT-f	5’-AAGCCGATGTACCTGTTCCG-3’
Msm-2889-RT-r	5’-CAGGATCAGCTTGGTGTTGC-3’
Msm-2969-RT-f	5’-CTGCCAACGGGTATCTGCT-3’
Msm-2969-RT-r	5’-CTCGAAGTCGTTGACCTTGG-3’
Msm-sodA -RT-f2	5’-CTGACCTTCCAGCTCTACGAC-3’
Msm-sodA -RT-r2	5’-TTGACGACGTTCCAGAAGGC-3’
Msm-sodB -RT-f	5’-TCGGCTCGTTCGACAACTTT-3’
Msm-sodB -RT-r	5’-CCAGTGGGACATTTGCTTGC-3’
Msm-sodC-RT-f	5’-CCACCACCGATGCGTTCA-3’
Msm-sodC-RT-r	5’-CATCGTCGTCTGATCCGGG-3’
Msm-furA-RT-f	5’-TACGAATCTCGCGTCGGC-3’

Msm-furA-RT-r	5’-ATGTGCCCCAGTAGATGACC-3’
Msm-katG-RT-f	5’-AACCCGGACGTCATCAATCC-3’
Msm-katG-RT-r	5’-ATGAACAGCGGTCCGTAGTG-3’
Msm-AhpC -RT-f	5’-TCGTGTACTTCTACCCGGCG-3’
Msm-AhpC -RT-r	5’-CCTCCTTGTCGCGGAACTTG-3’
mycgfp2-RT-f	5’-ATGTCGAAGGGCGAGGAGCTGTTCACCGGC-3’
mycgfp2-RT-r	5’-GAAGCACTGGACGCCGTAGGTCAGGGTGGTG-3’
Msm-16SrRNA-RT-f	5’-CTGAGATACGGCCCAGACTC-3’
Msm-16SrRNA-RT-r	5’-CATACCGTCACTTGCGCTTC-3’
Msm-mrx1-RT-F	5’-CTGAAGACCGCTCTCAAGGC-3’
Msm-mrx1-RT-R	5’-CTTGATGGTCGGGTTGGTCA-3’
Msm-gpx-RT-F	5’-CCAGGATCAGAACACCGACG-3’
Msm-gpx-RT-R	5’-TGTTCTTCTCGACACCGTTGAA-3’
Msm-tpx-RT-F	5’-AACCCCATCAACACCGTCGG-3’
Msm-tpx-RT-R	5’-CCGACGGGAAGATGTTCAGC-3’
Msm-egtA-RT-F	5’-TGATGCTGGTGAACTCTCCG-3’
Msm-egtA-RT-R	5’-GCGTCGTCAGGTGATAGTCC-3’

Supplementary Table 5. Oligonucleotide primers used for Cloning

**Name**	**Sequence**
Msm-6603-P1-f	5’-GCTCTAGACGAGTTCGCGGTACAGC-3’
Msm-6603-P1-r	5’- AGGATATCCCCGACCCCCTGAGTAG-3’

Supplementary References

Alting-Mees, M. A., and Short, J. M. (1989). pBluescript II: gene mapping vectors. *Nucleic. Acids Res.* **17**, 9494.

Ausubel, F., and Kingston, R. *Current Protocols in Molecular Biology*, (Eds), (Greene Publishing, New York, 1987).

Bhaskar, A., Chawla, M., Mehta, M., Parikh, P., Chandra, P., Bhave, D., et. al. (2014). Reengineering redox sensitive GFP to measure mycothiol redox potential of *Mycobacterium tuberculosis* during infection. *PLoS Pathog.* 10, e1003902. https://dx.doi.org/10.1371/journal.ppat.1003902

Farhana, A., Kumar, S., Rathore, S. S., Ghosh, P. C., Ehtesham, N. Z., Tyagi, A. K., et. al. (2008). Mechanistic insights into a novel exporter-importer system of *Mycobacterium tuberculosis* unravel its role in trafficking of iron. *PLoS One* 3, e2087. http://dx.doi.org/10.1371/journal.pone.0002087

Fisher, A. E., Maxwell, S. C., and Naughton, D. P. (2004). Superoxide and hydrogen peroxide suppression by metal ions and their EDTA complexes. *Biochem. Biophys. Res. Commun.* 316, 48-51. https://dx.doi.org/10.1016/j.bbrc.2004.02.013

Hirayama, T., Okuda, K., and Nagasawa, H. (2013). A highly selective turn-on fluorescent probe for iron (II) to visualize labile iron in living cells. *Chem. Sci.* 4, 1250-1256. http://dx.doi.org/10.1006/bbrc.1998.9729

Käser, M., Ruf, M. T., Hauser, J., Marsollier, L., and Pluschke, G. (2009). Optimized method for preparation of DNA from pathogenic and environmental mycobacteria. *Appl. Environ. Microbiol.* 75, 414-418. https://dx.doi.org/10.1128/AEM.01358-08.

Kennedy, M. C., Emptage, M. H., Dreyer, J. L., and Beinert, H. (1983). The role of iron in the activation-inactivation of aconitase. *J. Biol. Chem.* 258, 11098-11105.

Li, Y., and Trush, M. A. (1998). Diphenyleneiodonium, an NAD(P)H oxidase inhibitor, also potently inhibits mitochondrial reactive oxygen species production. *Biochem. Biophys. Res. Commun.* 253, 295-299. http://dx.doi.org/10.1006/bbrc.1998.9729

Mohanty, J. G., Jaffe, J. S., Schulman, E. S., and Raible, D. G. (1997). A highly sensitive fluorescent micro-assay of H_2O_2 release from activated human leukocytes using a dihydroxyphenoxazine derivative. *J. Immunol. Methods* 202, 133-141. http://dx.doi.org/10.1016/S0022-1759(96)00244-X

Morones-Ramirez, J. R., Winkler, J. A., Spina, C. S., and Collins, J. J. (2013). Silver enhances antibiotic activity against gram-negative bacteria. *Sci. Transl. Med.* 5, 190ra81. https://dx.doi.org/10.1126/scitranslmed.3006276

Motulsky, H. J. “Statistical principles” in *Prism 5 Statistics Guide* (GraphPad Software Inc., San Diego CA, www.graphpad.com,Version 5.0, 2007), pp 65.

Mukherjee, P., Sureka, K., Datta, P., Hossain, T., Barik, S., Das, K. P., et. al. (2009). Novel role of Wag31 in protection of mycobacteria under oxidative stress. *Mol. Microbiol.* 73, 103-119. https://dx.doi.org/10.1111/j.1365-2958.2009.06750.x

Nair, R. R., Sharan, D., Sebastian, J., Swaminath, S., and Ajitkumar, P. (2019). Heterogeneity in ROS levels in antibiotics-exposed mycobacterial subpopulations confer differential susceptibility. *Microbiology* (In Press)

Nazarewicz, R. R., Bikineyeva, A., and Dikalov, S. I. (2013). Rapid and specific measurements of superoxide using fluorescence spectroscopy. *J. Biomol. Screen.* 18, 498-503. https://dx.doi.org/10.1177/1087057112468765

Parker, N. B., Berger, E. M., Curtis, W. E., Muldrow, M. E., Linas, S. L., and Repine, J. E. (1985). Hydrogen peroxide causes dimethylthiourea consumption while hydroxyl radical causes dimethyl sulfoxide consumption *in vitro*. *Free Radic. Biol. Med.* 1, 415-419. http://dx.doi.org/10.1016/0748-5514(85)90155-2

Peshavariya, H. M., Dusting, G. J., and Selemidis, S. (2007). Analysis of dihydroethidium fluorescence for the detection of intracellular and extracellular superoxide produced by NADPH oxidase. *Free Radic. Res.* 41, 699-712. https://dx.doi.org/10.1080/10715760701297354

Reusch, V. M. Jr., and Burger, M. M. (1974). Distribution of marker enzymes between mesosomal and protoplast membranes. *J. Biol. Chem.* 249, 5337-5345.

Roberts, B., and Hirst, R. (1996). Identification and characterisation of a superoxide dismutase and catalase from *Mycobacterium ulcerans*. *J. Med. Microbiol.* 45, 383-387. http://dx.doi.org/10.1099/00222615-45-5-383

Roy, S., Narayana, Y., Balaji, K. N., and Ajitkumar, P. (2012). Highly fluorescent GFP_m^{2+}-based genome integration-proficient promoter probe vector to study *Mycobacterium tuberculosis* promoters in infected macrophages. *Microb. Biotechnol.* 5, 98-105. http://dx.doi.org/10.1111/j.1751-7915.2011.00305.x

Sebastian, J. (2016). *Response of Mycobacterium tuberculosis to Rifampicin – A Cellular, Molecular, and Ultrastructural Study*. Doctoral thesis, Indian Institute of Science, Bangalore, India.

Setsukinai, K., Urano, Y., Kakinuma, K., Majima, H. J., and Nagano, T. (2003). Development of novel fluorescence probes that can reliably detect reactive oxygen species and distinguish specific species. *J. Biol. Chem.* 278, 3170-3175. http://dx.doi.org/10.1074/jbc.M209264200

Snapper, S. B., Melton, R. E., Mustafa, S., Kieser, T., and Jacobs, W. R. Jr. (1990). Isolation and characterisation of efficient plasmid transformation mutants of *Mycobacterium smegmatis*. *Mol. Microbiol.* 4, 1911-1919. https://dx.doi.org/10.1111/j.1365-2958.1990.tb02040.x

Tadolini, B., and Cabrini, L. (1988). On the mechanism of $OH^•$ scavenger action. *Biochem. J.* 253, 931-932. http://dx.doi.org/10.1042/bj2530931

Tsugawa, H., Mori, H., Matsuzaki, J., Masaoka, T., Hirayama, T., Nagasawa, H., et. al. (2015). Nordihydroguaiaretic acid disrupts the antioxidant ability of *Helicobacter pylori* through the repression of SodB activity *in vitro*. *Biomed. Res. Int.* 2015, 734548. http://dx.doi.org/10.1155/2015/734548

Vijay, S., Nair, R. R., Sharan, D., Jakkala, K., Mukkayyan, N., Swaminath, S., et. al. (2017). Mycobacterial cultures contain cell size and density specific sub-populations of cells with significant differential susceptibility to antibiotics, oxidative and nitrite stress. *Front. Microbiol.* 8, 463. http://dx.doi.org/10.3389/fmicb.2017.00463

Wang, W., Chen, K., and Xu, C. (2006). DNA quantification using EvaGreen and a real-time PCR instrument. *Anal. Biochem.* 356, 303-305. https://dx.doi.org/10.1016/j.ab.2006.05.027

Wecker, E. (1959). The extraction of infectious virus nucleic acid with hot phenol. *Virology* 7, 241-243. https://dx.doi.org/10.1016/0042-6822(59)90191-6

Willems, E., Leyns, L., and Vandesompele, J. (2008). Standardization of real-time PCR gene expression data from independent biological replicates. *Anal. Biochem.* 379,127-129. https://dx.doi.org/10.1016/j.ab.2008.04.036

Xiao, R., and Kisaalita, W. S. (1997). Iron acquisition from transferrin and lactoferrin by *Pseudomonas aeruginosa* pyoverdine. *Microbiology* 143, 2509-2515. https://dx.doi.org/10.1099/00221287-143-7-2509

Yanisch-Perron, C., Vieira, J., and Messing, J. (1985). Improved M13 phage cloning vectors and host strains: nucleotide sequences of the M13mp18 and pUC19 vectors. *Gene* **33**, 103-119. https://doi.org/10.1016/0378-1119(85)90120-9

Yeware, A. M., Shurpali, K. D., Athalye, M. C., and Sarkar, D. (2017). Superoxide generation and its involvement in the growth of *Mycobacterium smegmatis*. *Front. Microbiol.* 8, 105. http://dx.doi.org/10.3389/fmicb.2017.00105

Zhou, M., Diwu, Z., Panchuk-Voloshina, N., and Haugland, R. P. (1997). A stable nonfluorescent derivative of resorufin for the fluorometric determination of trace hydrogen peroxide: applications in detecting the activity of phagocyte NADPH oxidase and other oxidases. *Anal. Biochem.* 253, 162-168. http://dx.doi.org/10.1006/abio.1997.2391
